# Cytotoxic Sesquiterpenoid Quinones and Quinols, and an 11-Membered Heterocycle, Kauamide, from the Hawaiian Marine Sponge *Dactylospongia elegans*

**DOI:** 10.3390/md17070423

**Published:** 2019-07-19

**Authors:** Ram P. Neupane, Stephen M. Parrish, Jayanti Bhandari Neupane, Wesley Y. Yoshida, M. L. Richard Yip, James Turkson, Mary Kay Harper, John D. Head, Philip G. Williams

**Affiliations:** 1 Department of Chemistry, University of Hawaii at Manoa, Honolulu, HI 96822, USA; 2 University of Hawaii Cancer Center, Honolulu, HI 96813, USA; 3 Department of Medicinal Chemistry, University of Utah, College of Pharmacy, Salt Lake City, UT 84112, USA

**Keywords:** sesquiterpenoid quinones, 11-membered heterocycle, *Dactylospongia elegans*, DFT computations, BACE1, human glioma, human pancreatic carcinoma

## Abstract

Several known sesquiterpenoid quinones and quinols (**1**–**9**), and kauamide (**10**), a new polyketide-peptide containing an 11-membered heterocycle, were isolated from the extracts of the Hawaiian marine sponge *Dactylospongia* *elegans*. The planar structure of **10** was determined from spectroscopic analyses, and its relative and absolute configurations were established from density functional theory (DFT) calculations of the GIAO NMR shielding tensors, and advanced Marfey’s analysis of the *N*-MeLeu residue, respectively. Compounds **1** and **3** showed moderate inhibition of β-secretase 1 (BACE1), whereas **1**–**9** exhibited moderate to potent inhibition of growth of human glioma (U251) cells. Compounds **1**–**2** and **4**–**7** were also active against human pancreatic carcinoma (Panc-1) cells.

## 1. Introduction

Since the discovery of unusual nucleosides from the sponge *Tethya crypta* by Bergmann and Feeney in the early 1950s [1], various classes of biologically-active secondary metabolites, including alkaloids, peptides, steroids and terpenes, have been isolated from marine sponges [2]. Sponge metabolites are of considerable pharmaceutical interest and they span a wide range of activities, such as anticancer, anti-inflammatory, antiviral, and antibacterial. These include three compounds that have been approved by the U.S. Food and Drug Administration (FDA) as clinical drugs [3]. 

**Figure 1:**
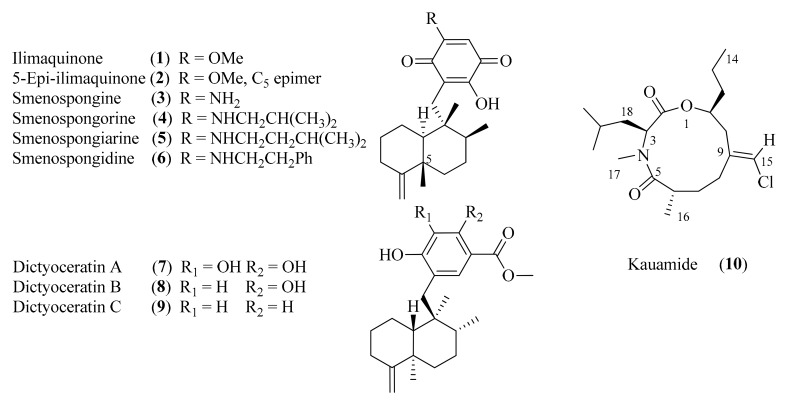
Structures of molecules isolated from *D. elegans*.

In our efforts to discover novel inhibitors of BACE1 (β-site of Amyloid precursor protein Cleaving Enzyme), an enzyme implicated in the pathogenesis of Alzheimer’s disease [4,5], we tested extracts from various marine organisms. In our investigations, the extract of a sample of *Dactylospongia elegans*, collected off the coast of Kauai, showed significant inhibition of BACE1 in vitro (66% and 73% inhibition by the 75% and 100% MeOH C8 fractions at 30 μg/mL, respectively). Bioactivity-guided separation resulted in the isolation of nine known sesquiterpenoid quinones and quinols (**1**–**9**), and kauamide (**10**), a new 11-membered heterocyclic compound (Figure 1).

Sponges of the genus *Dactylospongia* are well-known producers of sesquiterpenoid quinones and hydroquinones, including ilimaquinone [6,7,8], nakijiquinones [9,10,11], puupehenol [12] and others. Furthermore, these secondary metabolites, which possess either a drimane or a 4,9-friedo-rearranged skeleton, are known to exhibit various biological effects, such as cytotoxicity, antitumor, antiviral and anti-inflammatory activities [10,13,14,15,16]. However, little is known about inhibition of BACE1 by sesquiterpenoid quinones and hydroquinones or other secondary metabolites of marine origin.

Herein, we describe the structure elucidation of the new heterocyclic compound, and results from bioassays involving BACE1, human glioma (U251MG) and human pancreatic carcinoma (Panc-1) cells.

## 2. Results and Discussion

### 2.1. Isolation and Structural Characterization

In our quest to discover new inhibitors of BACE1 from marine organisms, it was found that an extract of *D. elegans* displayed activity against the enzyme in our in vitro assay system. Bioactivity-guided purification of the extract led to the isolation of ilimaquinone (**1**) [6,8], a sesquiterpene quinone containing a 4,9-friedo-rearranged drimane skeleton. Other sesquiterpene quinones and hydroquinones isolated from the extract included 5-*epi*-ilimaquinone (**2**) [17], smenospongine (**3**) [18], smenospongorine (**4**) [18], smenospongiarine (**5**) [18], smenospongidine (**6**) [18,19], dictyoceratin A (**7**), [18,20] dictyoceratin B (**8**) [20] and dictyoceratin C (**9**) [7,21]. The structures of these compounds were confirmed, except in one instance, by comparison of experimental spectroscopic data (Figures S2–S17) with the values reported in the literature. We discovered and confirmed by simple synthesis that the ^1^H and ^13^C NMR literature data for smenospongidine (**6**) contained obvious errors or omissions, which we have discussed in depth in a recent publication [22] (see the SI for details of the synthesis and characterization of the unreported major side product). 

In addition to **1**–**9**, which have previously been isolated from sponges of the genus *Dactylospongia*, a new chlorinated compound (**10**) with a rare 11-membered heterocyclic framework was also isolated. The chlorinated compound was present in an inactive fraction, but we nonetheless pursued the isolation and purification of this metabolite, which we have named kauamide. High resolution mass spectrometry of kauamide suggested a molecular formula of C_19_H_32_ClNO_3_, with four degrees of unsaturation. Two carbonyl units—an amide and an ester—were inferred from the ^13^C NMR chemical shifts at 177.4 ppm and 171.1 ppm as well as the infrared absorptions at 1734 cm^−1^ and 1653 cm^−1^. The appearance of two additional sp^2^ type signals in the ^13^C NMR spectrum at 138.9 ppm and 116.7 ppm indicated the presence of an olefin and implied that a ring fulfilled the degrees of unsaturation in the molecule. 

**Figure 2:**
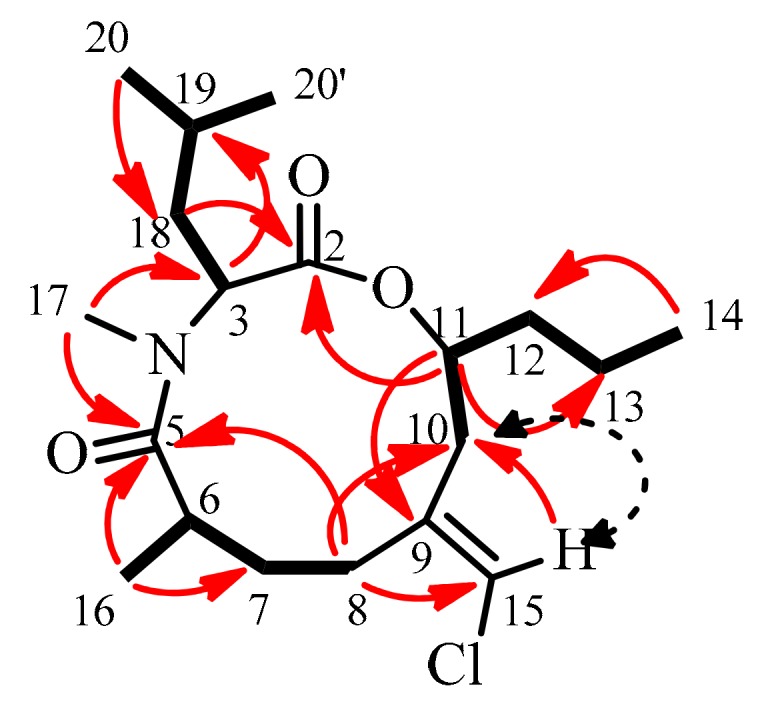
Depiction of COSY (bold lines), HMBC (red solid arrows) and NOESY (black dashed arrows) correlations in kauamide (**10**).

From the ^1^H, ^13^C and HSQC NMR data of kauamide (Table 1 and Figures S18–S24), five methyl groups, including one bonded to a nitrogen atom (*δ*_C_ 30.3, *δ*_H_ 2.84), six methylene units and four methine fragments were identified. Furthermore, the tri-substituted nature of the olefin was confirmed, as only one sp^2^ carbon was connected to a single hydrogen atom. The majority of the planar structure was put together based on the COSY and HMBC data (Figure 2), paving way for the placement of the chlorine atom on the terminal carbon of the exocyclic olefin. The *E*-olefin geometry was determined from the presence of NOESY correlation between H-15 and H-10 and the absence of such correlations between H-15 and H-8. 

With three stereogenic centers of kauamide separated from each other by quaternary carbons and heteroatoms in a non-rigid ring structure, we were unsuccessful in determining the relative stereochemical configuration in the molecule using empirical NMR methods. The absolute configuration of each stereogenic center could be assigned individually using irreversible (for practical purposes) chemical modifications of the compound—advanced Marfey’s method (C-3) [23,24,25], Mosher’s method (C-11) [26] and Kusumi’s method (C-6) [27], but the latter two require a considerable amount of sample for unambiguous determination with the aid of standard NMR equipment. We had isolated only 2 mg of kauamide, which was going to be insufficient to carry out these chemical modifications. Therefore, we turned to a protocol that relies on density functional theory-based computations of ^1^H and ^13^C NMR chemical shifts and the use of statistical tools to assign the experimental data to the correct isomer of a compound [28]. Similar approaches, sometimes in conjunction with synthesis, have been successfully employed toward assignment of stereochemical configurations of complex natural products, such as cernupalhine A [29], leiodermatolide [30] and gambierone [31], and revision of incorrectly assigned structures of nobilisitine A [32], mandelalide A [33], and hexacyclinol [34] to name a few. Recently, Grimblat and Sarotti have reviewed the role of GIAO NMR calculations on the structural assignment of complex molecules [35].

**Figure 3:**
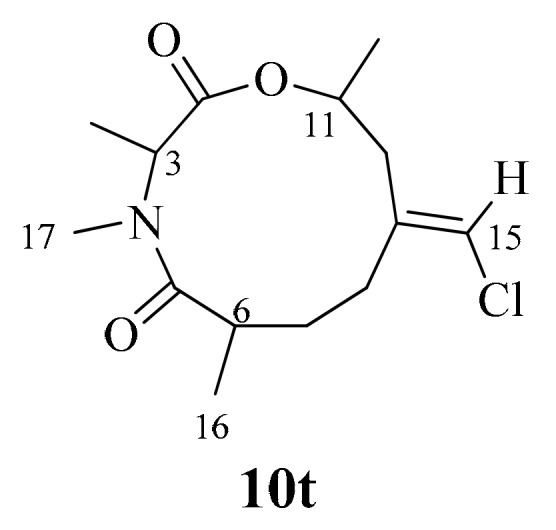
Structure of a truncated kauamide (**10t**) used in the computations of NMR shifts.

In our hands, the floppy nature of the two alkyl groups at C-3 and C-11 would amass a high computational cost, because each diastereomer of kauamide resulted in more than two hundred and fifty conformers within 5 kcal/mol of the lowest energy conformer. To simplify the computational operations, we truncated the alkyl groups at those positions to methyl groups, as shown in the structure of **10t** (Figure 3), which resulted in an approximately ten-fold reduction in the number of conformers. Conformers of **10t** within 5 kcal/mol of the lowest energy conformer were identified using the Monte Carlo multiple minimum (MCMM) method and the OPLS-2005 force field in MacroModel (Schrodinger Inc.). Each conformer within 5 kcal/mol of the lowest energy conformer was optimized in Gaussian09 [36] at the M06-2X/6-31+G level and the geometries of all conformers with similar energies were checked for redundancy (See Tables S3–56 for XYZ coordinates and Boltzmann distributions of conformers). NMR shielding tensors of all unique conformers within the energy window were computed using the gauge-independent atomic orbital (GIAO) method at the B3LYP/6-311+G level and ^1^H and ^13^C chemical shifts were obtained after applying appropriate scaling factors (^1^H: intercept = 31.9477, slope = −1.0767; ^13^C: intercept = 181.2412, slope = −1.0522).

An unambiguous match between the experimentally obtained ^1^H and ^13^C shifts of kauamide and the predicted values of the (3*S*,6*S*,11*S*)-diastereomer of **10t** (Table 2 and Table S2) was established from various statistical comparisons between empirical and computed data. The chemical shifts of this diastereomer had the best fit to the ^1^H and ^13^C data of kauamide by any statistical measure, including mean absolute error (MAE), DP4 [37] and DP4+ [38,39] probabilities (Table 3). The DP4 and DP4+ methods are widely used to assess the fit of GIAO NMR chemical shift calculations for various diastereomers against experimental data by assessing the likelihood that the deviations between the datasets are due to chance given an expected t distribution of errors, with the DP4+ method having improved how sp^2^ carbons and scaled NMR data are handled. From these studies and subsequent statistical analyses, we propose that kauamide bears the (3*S*,6*S*,11*S*) relative configuration. The absolute configuration, (3*S*,6*S*,11*S*) and as drawn on **10**, was established after hydrolysis of kauamide and subsequent confirmation of the L-configuration of leucine residue by advanced Marfey’s analysis.

Kauamide, which is a mixed polyketide-peptide natural product, has a structural framework, including the vinyl chloride motif, which suggests that it may have been produced by a marine cyanobacterium. The 11-membered ring, however, is very rare in this class of molecules and natural products in general. The closest structural similarities are observed in jamaicamides A-C [40], which were isolated from *Moorea producens* Engene (Oscillatoriaceae) [41], a cyanobacterium formerly identified as *Lyngbya majuscula* and in kanamienamide [42], which was isolated from the cyanobacterium *Moorea bouillonii* Engene (Oscillatoriaceae). These precedents strongly suggest that kauamide was produced by cyanobacteria cohabitating in the heavily fouled specimen of *D. elegans*. The polyketide fragment in kauamide has the same carbon framework and identical olefin geometry as in the C-1-C-10 segment of jamaicamides A-C, whereas the 11-membered ring including the leucine residue is present in kanamienamide. Kanamienamide, however, lacks the vinyl chloride motif and has opposite configuration at C-6, the carbon alpha to the amide carbonyl in the ring. The ^1^H and ^13^C NMR data for kanamienamide were acquired in benzene-*d*_6_, precluding a direct comparison with kauamide of the chemical shifts for protons and carbons in the 11-membered ring. Nevertheless, there is a reasonable match between the predicted ^1^H NMR chemical shifts of (3*S*,6*R*,11*S*)-diastereomer of **10t** and the experimental values reported for kanamienamide, which bears the same configuration at the specified stereogenic centers. This provides further support to the assignment of relative configuration of kauamide through prediction of NMR chemical shifts.

### 2.2. Biological Evaluation of the Isolated Compounds

Ilimaquinone (**1**) and smenospongine (**3**) displayed moderate inhibition of BACE1, whereas the other analogs showed weak or no activity (Table 4 and Table S1). The level of activity noted for the isolated compounds is inconsistent with the degree of BACE1 inhibition observed in the initial assay suggesting either degradation of the active compound(s) or these two hits were false positives in the initial assay.

Sesquiterpene quinones and hydroquinones are known to be cytotoxic to a variety of cancer cell lines, which was reaffirmed when we screened these compounds against human glioblastoma (U251MG) and human pancreatic carcinoma (Panc-1) cell lines. Compounds **1**–**9** all exhibited considerable cytotoxicity toward U251MG cells (Figure S1 and Table S1), with smenospongine (**3**) and dictyoceratin A (**7**) showing the most potent effects. Furthermore, **1**–**2** and **4**–**7** were significantly cytotoxic toward Panc-1 cells as well (Figure S2). Data for **4**, **5**, and **9** against PANC1 are consistent with a recent literature report [43]. Kauamide (**10**) showed no significant biological activity in any of the two of the assays (60% inhibition at 50 µM against U251; 15% inhibition at 83 μM against BACE1). It was not tested against PANC-1.

In conclusion, chemical investigations of an extract from the sponge *Dactylospongia elegans* led to the isolation of several known and a new natural product. The nine known sesquiterpene quinones and hydroquinones **1**–**9** isolated from the sponge *D. elegans* showed strong to moderate cytotoxicity against the human glioma (U251MG) cancer cell line. Additionally, six of these compounds exhibited moderate cytotoxicity against the human pancreatic carcinoma (Panc-1) cell line. Furthermore, ilimaquinone and smenospongine displayed moderate inhibition of BACE1, an enzyme implicated in the pathogenesis of the Alzheimer’s disease. The significance of these findings is that they represent new reports of biological activity of the known compounds against a previously untested target. A new 11-membered heterocycle, kauamide (**10**), was also isolated from the sponge extract. Literature reports of isolation of structurally similar metabolites from cyanobacteria of the genus *Moorea*, however, suggest that kauamide is in most likelihood biosynthesized by a cyanobacterium. Regardless, the discovery of kauamide illustrates the potential in the discovery of new natural products from the marine environment.

## 3. Materials and Methods 

### 3.1. General Experimental Procedures

Optical rotations were measured on a DIP-370 polarimeter (JASCO, Oklahoma, OK, USA) at 589 nm using a sample cell of path length 0.1 dm. UV spectra were obtained on a Cary 50 Bio UV-Visible spectrophotometer (Varian, Palo Alto, CA, USA). IR spectra of the compounds, as thin films on CaF_2_ discs, were recorded using an IRAffinity-1 Fourier Transform spectrophotometer (Shimadzu, Kyoto, Japan). NMR spectra were acquired on an Inova 500 MHz spectrometer (Varian, Palo Alto, CA, USA) operating at 500 (^1^H) or 125 (^13^C) MHz using the residual solvent signals as internal references (CD_3_OD δ_H_ 3.30, δ_C_ 49.0; DMSO-*d*_6_ δ_H_ 2.50, δ_C_ 39.5; CDCl_3_ δ_H_ 7.26, δ_C_ 77.0). Samples were placed in 3-mm Shigemi NMR tubes as necessary. HRMS data were obtained from 6210 LC/TOF and 6545 LC/QTOF instruments (Agilent, Santa Clara, CA, USA) using electrospray ionization in positive and negative modes. ESIMS data were also obtained using a 6410 QQQ instrument (Agilent, Santa Clara, CA, USA). Samples were purified using a combination of chromatographic techniques, including high performance liquid chromatography. Purity of the compounds was assessed from their UV absorptions at appropriate wavelengths, or from their ^1^H NMR spectra. Percent yields of natural products were based on the amount of freeze-dried biological material.

### 3.2. Chemicals and Reagents

Assays were performed according to manufacturer’s instructions. The BACE1 assay kit was purchased from DiscoveRx (Fremont, CA, USA). ACS grade solvents (Fisher Scientific, Waltham, MA, USA) were distilled and filtered before use. HP-20 resin, flash column stationary phases, and HPLC columns were purchased from Supelco (St. Louis, MO, USA), Sorbent Technologies (Atlanta, GA, USA), and Phenomenex (Torrance, CA, USA), respectively.

### 3.3. Biological Material

A specimen of the sponge *Dactylospongia elegans* (Sample ID: *10-Kauai-08*, Figure S25) was collected from a depth of 40 feet at the Sheraton Caverns, Kauai by SCUBA diving on June 09, 2010. The sponge was fouled green in its natural habitat and exuded red solution in the collection bag. Recollection of the same sponge (Sample ID: *11-Kauai-80*) from the same location on May 29, 2011 was confirmed upon comparison of collection details, sample description and pictures, and LC-MS profiles of their crude extracts. The biological material was freeze-dried and stored in a freezer prior to extraction. A voucher sample of *10-Kauai-08* was preserved in aqueous alcohol and identified by Mary Kay Harper (University of Utah, Salt Lake City, UT, USA).

### 3.4. Extraction and Isolation of Metabolites from Dactylospongia Elegans

The freeze-dried sponge sample (40 g) was extracted three times in a 1:1 mixture of methanol and dichloromethane to yield 6.82 g of crude extract, which was then partitioned with hexanes, dichloromethane, butanol and aqueous methanol. 

The fraction obtained from the partition with hexanes (1.32 g) was subjected to solid phase extraction on C8 support, in which the extract was eluted sequentially with an increasing concentration of methanol in water, followed by isopropanol. The fraction obtained from the elution with methanol (0.45 g) was divided into six subfractions by flash chromatography (Silica St. Louis, MO, USA), 250 × 50 mm, eluted in the following order: 0:10, 1:9, 2:8, 3:7, 10:0 mixtures of ethyl acetate to hexane, and isopropanol.) The second subfraction (52 mg) was purified by RP-HPLC (Phenomenex Luna C18 (Torrance, CA, USA), 150 × 4.6 mm, 5 μm particle size, 100 Å; eluted with MeCN/H_2_O at 0.7 mL/min using a gradient of 70% to 100% MeCN over 20 min followed by a wash with MeCN for 10 min) to afford kauamide (**10**, *t*_R_ 16.0 min, 2.0 mg, 0.005% yield, >95% purity by ^1^H NMR). The third subfraction (110 mg) was purified by RP-HPLC (Phenomenex Luna C18 (Torrance, CA, USA), 250 × 10 mm, 5 μm particle size, 100 Å; eluted with MeCN/H_2_O at 2.5 mL/min using the following gradient: 70% to 90% MeCN over 15 min, held at that composition for 10 min, then increased to 100% MeCN over 5 min and washed with MeCN for an additional 15 min) to afford dictyoceratin C (**9**, *t*_R_ 26.7 min, 3.5 mg, 0.009% yield, >90% purity by ^1^H NMR), dictyoceratin B (**8**, *t*_R_ 29.3 min, 4.0 mg, 0.01% yield, >99% purity at 272 nm), smenospongorine (**4**, *t*_R_ 38.0 min, 3.5 mg, 0.009% yield, >99% purity at 313 nm) and smenospongiarine (**5**, *t*_R_ 41.3 min, 5.5 mg, 0.014% yield, 99% purity at 313 nm). The fifth subfraction (27 mg) obtained from the flash chromatographic separation was subjected to an identical purification scheme to afford dictyoceratin A (**7**, *t*_R_ 24.1 min, 4.5 mg, 0.011% yield, >99% purity at 260 nm).

The fraction obtained from partition with dichloromethane (0.98 g) was also subjected to solid phase extraction on C8 support, in which the extract was eluted sequentially with an increasing concentration of methanol in water, followed by isopropanol. The fraction obtained from the elution with 75% methanol (0.28 g) was purified by RP-HPLC (Phenomenex Luna C18 (Torrance, CA, USA), 150 × 4.6 mm, 5 μm particle size, 100 Å; eluted with MeCN/H_2_O at 2.5 mL/min using a gradient of 90% to 100% MeCN over 25 min followed by a wash with MeCN for 10 min) to afford 5-*epi*-ilimaquinone (**2**, *t*_R_ 20.1 min, 10.0 mg, 0.025% yield, 78% purity by ^1^H NMR), ilimaquinone (**1**, *t*_R_ 21.7 min, 98.0 mg, 0.245% yield, >99% purity at 310 nm), smenospongine (**3**, *t*_R_ 22.5 min, 3.5 mg, 0.009% yield, 95% purity at 310 nm) and smenospongidine (**6**, *t*_R_ 29.8 min, 6.5 mg, 0.016% yield, >99% purity at 310 nm).

### 3.5. Kauamide (***10***)

White, amorphous solid; ${\left[\alpha \right]}_{D}^{22}$ + 20 (*c* 0.1, CHCl_3_); UVs (CH_3_OH) *λ*_max_ (log ε) 201 (4.08) nm; IR (CaF_2_ disc) *ν*_max_ 2958, 2927, 2870, 1734, 1653 and 1641 cm^−1^; ^1^H NMR (CDCl_3_, 500 MHz) and ^13^C NMR (CDCl_3_, 125 MHz) see Table 1; HRESIMS *m*/*z* 358.2140 [M + H]^+^ (calcd. for C_19_H_33_ClNO_3_, 358.2143).

### 3.6. Hydrolysis of Kauamide (***10***) and Advanced Marfey’s Analysis

A solution of 200 μg (0.6 μmol, 1 equiv.) of kauamide (**10**) in 0.5 mL 6N HCl in a capped reaction vial was stirred for 24 h at 110 °C. Solvent was removed by streaming N_2_ over the reaction mixture and the residue was analyzed by ESIMS in negative mode to confirm the presence of signals at *m*/*z* 247 and 249. To the dried hydrolysate in the vial were added a solution of 500 μg (1.6 μmol, 2.7 equiv.) 1-fluoro-2,4-dinitrophenyl-5-l-leucinamide (L-FDLA) in acetone (160 μL), H_2_O (100 μL) and 1N solution of NaHCO_3_ (50 μL). The contents in the vial were warmed to 40 °C for 1 h, cooled to room temperature, and analyzed by LC-MS (EclipsePlus C18 column (Agilent, Santa Clara, CA, USA), 2.1 × 50 mm, 1.8 µm particle size; eluted with acetonitrile and water (+0.1% formic acid in each) at 0.4 mL/min flow using the following gradient: 20–50% acetonitrile (0–10 min), 50–100% acetonitrile (10–12 min), 100% acetonitrile (12–16 min); detection at 340 nm). The LC-MS data was compared to the data obtained from similar protocols on the standard amino acids DL-*N*-methylleucine and L-*N*-methylleucine, which were obtained from the amino acid standards collection in the Williams laboratory. The retention times for the LL-adduct and the DL-adduct were 9.2 min and 10.3 min, respectively. 

### 3.7. NMR Shift Computations

Conformers within 5 kcal/mol of the lowest energy conformer were searched using the Monte Carlo multiple minimum (MCMM) method and the OPLS-2005 force field in MacroModel [44] (Version 10, Schrodinger Inc., New York, NY, USA). Each conformer within 5 kcal/mol of the lowest energy conformer was optimized in Gaussian09 (Version C.01, Wallingford, CT, USA) [36] at the M06-2X [45]/6-31+G level and the geometries of all conformers with similar energies were checked for redundancy. NMR shielding tensors of all unique conformers within the energy window were computed using the gauge-independent atomic orbital (GIAO) method at the B3LYP [46,47,48,49]/6-311+G [50,51] level and ^1^H and ^13^C chemical shifts were obtained after applying appropriate scaling factors (^1^H: intercept = 31.9477, slope = −1.0767; ^13^C: intercept = 181.2412, slope = −1.0522). Statistical comparisons of the computed shifts with the experimental data were carried out using the applet provided by Goodman [37] and the spreadsheet provided by Sarotti [38].

### 3.8. BACE1 Assay

The HitHunter BACE1 enzyme fragment complementation chemiluminescence assay was purchased from DiscoveRx (Fremont, CA, USA). The proteolytic cleavage of amyloid precursor protein was assayed as described by Naqvi [52]. Test compounds were solubilized in DMSO at the desired concentration, serially diluted as required to determine the IC_50_ value, and incubated with the enzyme in triplicates for 16 h in 96-well plates. A DMSO control (1.5 μL) and an inhibitor standard (*β*-secretase inhibitor IV from Calbiochem (San Diego, CA, USA) used as a positive control) were also tested in triplicates. An initial test concentration of 30 μg/mL was used for all compounds, with subsequent dose response curves determined for any compound that showed greater than 50% inhibition at that initial test concentration. The chemiluminescence signal was read using a Fluostar Optima spectrophotometer (Cary, NC, USA). Data were analyzed using GraphPad Prism (Version 5, San Diego, CA, USA). BACE1 activity was calculated as a percent of the positive control using a nonlinear regression analysis function that corresponded to a best one-fit model. Test concentrations and percent inhibition for the inactives are as follows: **4** (17% inhibition at 75 μM), **5** (5% inhibition at 73 μM), **6** (0% inhibition at 67 μM), **7** (44% inhibition at 80 μM), **8** (17% inhibition at 77 μM), **9** (0% inhibition at 84 μM), **10** (15% inhibition at 83 μM).

### 3.9. Cytotoxicity Assays

Human glioblastoma (U251MG) cell lines were maintained in RPMI 1640 Medium (Gibco, Dublin, Ireland) supplemented with 10% premium fetal bovine serum (Atlanta biological, Atlanta, GA, USA) and 100 U/mL penicillin and 100 µg/mL streptomycin (Gibco, Dublin, Ireland). A day prior to treatment, cancer cells were seeded at 4000 cells per well into a 96-well tissue culture plate. Twenty hours post seeding, the serially diluted compounds were added to the cells for the cytotoxicity assay, and co-incubated with 5% CO_2_ at 37 °C for 72 h. Then the medium with compounds were replaced with 1 × dye binding solution prepared according to the manufacturer’s instruction (CyQuant NF Cell Proliferation Assay Kit, Invitrogen, Carlsbad, CA, USA) and incubated with 5% CO_2_ at 37 °C for 60 min. After that, cell viability data were collected with a Multimode Plate Reader (PerkinElmer, Waltham, MA, USA) according to the manufacturer’s instruction. CC_50_ curves were generated using GraphPad Prism (Version 5, San Diego, CA, USA).

Human pancreatic carcinoma (Panc-1) cell lines were maintained in DMEM media supplemented with 10% premium fetal bovine serum and 50 U/mL penicillin and 50 µg/mL streptomycin. One day before treatment, cancer cells were seeded at 5000 cells per well into a 96-well tissue culture plate. Twenty-four hours post seeding, the serially diluted compounds were added to the cells for the cytotoxicity assay, and incubated at 37 °C with 5% CO_2_ for 72 h. Then 40 µL MTS dye (CellTiter aqueous One Solution Cell Proliferation Assay, Promega, Madison, WI, USA) was added to each well and incubated with 5% CO_2_ at 37 °C for 90 min. Cell viability data were collected with a Modulus Microplate Reader (Promega, Madison, WI, USA) and CC_50_ curves were generated using GraphPad Prism (version 5, San Diego, CA, USA).

## Figures and Tables

**Table 1:** ^1^H and ^13^C NMR Spectroscopic Data of Kauamide (**10**) in CDCl_3_ (500 and 125 MHz).

Position	*δ*_C_, type	*δ*_H_ (*J* in Hz)	COSY	HMBC ^a^
2	171.1, C			
3	55.7, CH	5.49, dd (11.5, 4.9)	15	2, 5, 16, 18
5	177.4, C			
6	36.0, CH	3.11, m		
7	26.5, CH_2_	1.68, m		
		2.07, m	6, 8	5
8	26.1, CH_2_	2.50, m		
		1.86, t (12.0)		9, 10
9	138.9, C			
10	40.2, CH_2_	2.36, dd (14.5, 4.2)	11	9, 20
		2.28, dd (14.5, 2.8)		
11	73.5, CH	4.98, m		2, 10, 13
12	34.6, CH_2_	1.52, ddd (15.6, 13.5, 6.9)	11, 13	
		1.43, m		
13	19.0, CH_2_	1.26, m		
14	13.8, CH_3_	0.92, t (7.3)	13	12
15	116.7, CH	5.83, s		8, 10
16	14.3, CH_3_	1.14, d (6.7)	6	5
17	30.3, CH_3_	2.84, s		3, 5
18	35.7, CH_2_	1.79, m (14.8, 10.1, 4.9)		
		1.68, m		2
19	24.7, CH	1.58, m	18, 20, 20’	
20/20’	23.3, CH_3_	0.97, d (6.7)		18
20’/20	21.1, CH_3_	0.95, d (6.7)		18

^a^ HMBC correlations, optimized for 7 Hz, are from proton(s) stated to the indicated carbon.

**Table 2:** ^1^H and ^13^C NMR chemical shifts of kauamide (**10**, experimental) and all four diastereomers of **10t** (predicted by DFT computations).

		Computed						Computed			
H	Experimental	*SRR*	*SSR*	*SRS*	*SSS*	C	Experimental	*SRR*	*SSR*	*SRS*	*SSS*
3	5.49	3.42	4.20	4.38	**5.36**	2	171.1	170.4	170.7	172.0	**171.4**
6	3.11	2.78	2.15	2.72	**3.07**	3	55.7	60.1	57.0	56.6	**52.9**
7a	2.07	1.74	1.88	1.91	**2.12**	5	177.4	174.8	174.8	173.1	**174.3**
7b	1.68	1.54	1.50	1.54	**1.67**	6	36.0	38.1	37.0	37.5	**37.1**
8a	1.86	2.12	1.82	1.81	**1.98**	7	26.5	32.1	31.6	29.8	**26.2**
8b	2.50	2.68	2.77	2.73	**2.52**	8	26.1	26.7	28.7	29.3	**26.9**
10a	2.36	1.98	2.10	2.19	**2.43**	9	138.9	145.1	142.0	145.2	**144.1**
10b	2.28	2.79	2.59	2.39	**2.18**	11	73.5	68.0	69.4	74.4	**69.9**
11	4.98	5.13	5.31	4.60	**4.82**	10	40.2	39.1	39.3	42.4	**42.2**
15	5.83	5.73	6.04	5.94	**5.86**	15	116.7	116.5	121.2	119.5	**119.3**
16	1.14	1.01	1.14	1.13	**1.09**	16	14.3	15.4	15.7	15.6	**12.7**
17	2.84	3.05	3.15	2.80	**2.74**	17	30.3	34.6	30.8	27.4	**28.8**

Key: *SRR* = (3*S*,6*R*,11*R*)-**10t**; *SSR* = (3*S*,6*S*,11*R*)-**10t**; *SRS* = (3*S*,6*R*,11*S*)-**10t**; *SSS* = (3*S*,6*S*,11*S*)-**10t**.

**Table 3:** The (3*S*,6*S*,11*S*) relative configuration of kauamide (**10**) was established by statistical analyses of the computed and experimental ^1^H and ^13^C NMR shifts of all possible diastereomers of **10t**.

Isomer	MAE, ppm (^1^H/^13^C)	DP4 Probability	DP4+ Probability
(3*S*,6*R*,11*R*)-**10t**	0.40/2.9	0.00	0.00
(3*S*,6*S*,11*R*)-**10t**	0.36/2.3	0.00	0.00
(3*S*,6*R*,11*S*)-**10t**	0.24/2.5	0.00	0.00
(3*S*,6*S*,11*S*)-**10t**	**0.07/2.1**	**1.00**	**1.00**

**Table 4:** Biological activities of metabolites isolated from *D. elegans*.

Compound	IC_50_ (BACE1) ^a^	CC_50_ (U251MG)	CC_50_ (Panc-1)
**1**	65 µM	19.3 µM	20.4 µM
**2**	--- ^b^	19.4 µM	16.2 µM
**3**	78 µM	2.4 µM	--- ^b^
**4**		19.4 µM	22.6 µM
**5**		4.5 µM	15.1 µM
**6**		4.0 µM	12.6 µM
**7**		2.8 µM	21.7 µM
**8**		8.4 µM	54.6 µM
**9**		4.1 µM	88.9 µM

^a^ IC_50_ values were not determined for **4**–**9** since they showed no significant BACE1 inhibition at 30 µg/mL ^b^ Not tested.

## Supplementary Materials

The following are available online at https://www.mdpi.com/1660-3397/17/7/423/s1. Figures S1–S25, Tables S1–S56: copies of all NMR data, NMR data for known compounds, picture of the biological sample, and XZY coordinates for conformers and Boltzmann distributions.
