# Supplementary material for: Cytotoxic Sesquiterpenoid Quinones and Quinols, and an 11-Membered Heterocycle, Kauamide, from the Hawaiian Marine Sponge Dactylospongia elegans
Source: Mar Drugs. 2019 Jul 19;17(7):423. doi: 10.3390/md17070423 (PMC6669564; doi:10.3390/md17070423)

Cytotoxic Sesquiterpenoid Quinones and Quinols, and an 11-Membered Heterocycle, Kauamide, from the Hawaiian Marine Sponge *Dactylospongia elegans*

Ram P. Neupane[†], Stephen M. Parrish[†], Jayanti Bhandari Neupane [†],Wesley Y. Yoshida[†], M.L. Richard Yip[‡], James D. Turkson[‡], Mary Kay Harper [§], John D. Head[†], Philip G. Williams[†‡*]

Affiliation

† Department of Chemistry, University of Hawaii at Manoa, 2545 McCarthy Mall, Honolulu, HI 96822

‡ University of Hawaii Cancer Center, 701 Ilalo Street, Honolulu, HI 96813

§ Department of Medicinal Chemistry, University of Utah, Salt Lake City, UT 84112

Corresponding Author

Prof. Dr. Philip Williams, Department of Chemistry, University of Hawaii at Manoa, Honolulu, Hawaii, USA, 96822. E-mail: philipwi@hawaii.edu Phone: 808 956 5720 Fax: 808 956 5908

TABLE OF CONTENTS

Page

Details on known compounds 5
Figure S1. Biological Evaluation of Compounds 1-10 in U251MG and BACE1 Assays 10
Table S1. Biological evaluation data 10
Figure S2. Cytotoxicity Curves of Selected Compounds in Panc-1 Assay 11
Table S2. Summary of Experimental and Computed ^{1}H and ^{13}C NMR shifts of all possible diastereomers of 10 12
Figure S3. ^{1}H NMR Spectrum (500 MHz) of 1 in $CDCl_3$ 13
Figure S4. ^{13}C NMR Spectrum (125 MHz) of 1 in $CDCl_3$ 14
Figure S5. ^{1}H NMR Spectrum (500 MHz) of 2 in $CDCl_3$ 15
Figure S6. ^{1}H NMR Spectrum (500 MHz) of 3 in $CDCl_3$ 16
Figure S7. ^{1}H NMR Spectrum (500 MHz) of 4 in $CDCl_3$ 17
Figure S8. ^{13}C NMR Spectrum (125 MHz) of 4 in $CDCl_3$ 18
Figure S9. ^{1}H NMR Spectrum (500 MHz) of 5 in $CDCl_3$ 19
Figure S10. ^{13}C NMR Spectrum (125 MHz) of 5 in $CDCl_3$ 20
Figure S11. ^{1}H NMR Spectrum (500 MHz) of 6 in $CDCl_3$ 21
Figure S12. ^{13}C NMR Spectrum (125 MHz) of 6 in $CDCl_3$ 22
Figure S13. ^{1}H NMR Spectrum (500 MHz) of 7 in $CDCl_3$ 23
Figure S14. ^{13}C NMR Spectrum (125 MHz) of 7 in $CDCl_3$ 24
Figure S15. ^{1}H NMR Spectrum (500 MHz) of 8 in $CDCl_3$ 25
Figure S16. ^{13}C NMR Spectrum (125 MHz) of 8 in $CDCl_3$ 26
Figure S17. ^{1}H NMR Spectrum (500 MHz) of 9 in $CDCl_3$ 27
Figure S18. ^{1}H NMR Spectrum (500 MHz) of 10 in $CDCl_3$ 28
Figure S19. ^{13}C NMR Spectrum (125 MHz) of 10 in $CDCl_3$ 29
Figure S20. gHSQC Spectrum (500 MHz) of 10 in $CDCl_3$ 30
Figure S21. gCOSY NMR Spectrum (500 MHz) of 10 in $CDCl_3$ 31
Figure S22. gHMBC NMR Spectrum (500 MHz) of 10 in $CDCl_3$ 32
Figure S23. ^{1}H NMR Spectrum (500 MHz) of 11 in $CDCl_3$ 33
Figure S24. ^{13}C NMR Spectrum (125 MHz) of 11 in $CDCl_3$ 34
Figure S25. Photograph of the Biological Specimen 35
Table S3. Boltzmann Distribution of Conformers of (3*S*,6*S*,11*S*)-10t 36
Table S4. Cartesian Coordinates For The Optimized Conformer 18 Of Compound 3*S*,6*S*,11*S*-10t 37
Table S5. Cartesian Coordinates For The Optimized Conformer 16 Of Compound 3*S*,6*S*,11*S*-10t 38
Table S6. Cartesian Coordinates For The Optimized Conformer 4 Of Compound 3*S*,6*S*,11*S*-10t 39
Table S7. Cartesian Coordinates For The Optimized Conformer 2 Of Compound 3*S*,6*S*,11*S*-10t 40
Table S8. Cartesian Coordinates For The Optimized Conformer 20 Of Compound 3*S*,6*S*,11*S*-10t 41
Table S9. Cartesian Coordinates For The Optimized Conformer 19 Of Compound 3*S*,6*S*,11*S*-10t 42
Table S10. Cartesian Coordinates For The Optimized Conformer 5 Of Compound 3*S*,6*S*,11*S*-10t 43
Table S11. Boltzmann Distribution of Conformers of (3R,6*S*,11*S*)-10t 44

Table S12. Cartesian Coordinates For The Optimized Conformer 1 Of Compound 3*R*,6*S*,11*S*-10t ... 45
Table S13. Cartesian Coordinates For The Optimized Conformer 4 Of Compound 3*R*,6*S*,11*S*-10t ... 46
Table S14. Cartesian Coordinates For The Optimized Conformer 8 Of Compound 3*R*,6*S*,11*S*-10t ... 47
Table S15. Cartesian Coordinates For The Optimized Conformer 14 Of Compound 3*R*,6*S*,11*S*-10t ... 48
Table S16. Cartesian Coordinates For The Optimized Conformer 5 Of Compound 3*R*,6*S*,11*S*-10t ... 49
Table S17. Cartesian Coordinates For The Optimized Conformer 2 Of Compound 3*R*,6*S*,11*S*-10t ... 50
Table S18. Cartesian Coordinates For The Optimized Conformer 3 Of Compound 3*R*,6*S*,11*S*-10t ... 51
Table S19. Cartesian Coordinates For The Optimized Conformer 7 Of Compound 3*R*,6*S*,11*S*-10t ... 52
Table S20. Cartesian Coordinates For The Optimized Conformer 15 Of Compound 3*R*,6*S*,11*S*-10t ... 53
Table S21. Cartesian Coordinates For The Optimized Conformer 6 Of Compound 3*R*,6*S*,11*S*-10t ... 54
Table S22. Cartesian Coordinates For The Optimized Conformer 13 Of Compound 3*R*,6*S*,11*S*-10t ... 55
Table S23. Cartesian Coordinates For The Optimized Conformer 9 Of Compound 3*R*,6*S*,11*S*-10t ... 56
Table S24. Cartesian Coordinates For The Optimized Conformer 10 Of Compound 3*R*,6*S*,11*S*-10t ... 57
Table S25. Boltzmann Distribution of Conformers of (3R,6*S*,11*R*)-10t ... 58
Table S26. Cartesian Coordinates For The Optimized Conformer 10 Of Compound 3*R*,6*S*,11*R*-10t ... 59
Table S27. Cartesian Coordinates For The Optimized Conformer 21 Of Compound 3*R*,6*S*,11*R*-10t ... 60
Table S28. Cartesian Coordinates For The Optimized Conformer 3 Of Compound 3*R*,6*S*,11*R*-10t ... 61
Table S29. Cartesian Coordinates For The Optimized Conformer 13 Of Compound 3*R*,6*S*,11*R*-10t ... 62
Table S30. Cartesian Coordinates For The Optimized Conformer 5 Of Compound 3*R*,6*S*,11*R*-10t ... 63
Table S31. Cartesian Coordinates For The Optimized Conformer 16 Of Compound 3*R*,6*S*,11*R*-10t ... 64
Table S32. Cartesian Coordinates For The Optimized Conformer 12 Of Compound 3*R*,6*S*,11*R*-10t ... 65
Table S33. Cartesian Coordinates For The Optimized Conformer 8 Of Compound 3*R*,6*S*,11*R*-10t ... 66
Table S34. Cartesian Coordinates For The Optimized Conformer 4 Of Compound 3*R*,6*S*,11*R*-10t ... 67
Table S35. Cartesian Coordinates For The Optimized Conformer 18 Of Compound 3*R*,6*S*,11*R*-10t ... 68
Table S36. Cartesian Coordinates For The Optimized Conformer 9 Of Compound 3*R*,6*S*,11*R*-10t ... 69
Table S37. Cartesian Coordinates For The Optimized Conformer 19 Of Compound 3*R*,6*S*,11*R*-10t ... 70
Table S38. Cartesian Coordinates For The Optimized Conformer 15 Of Compound 3*R*,6*S*,11*R*-10t ... 71
Table S39. Cartesian Coordinates For The Optimized Conformer 11 Of Compound 3*R*,6*S*,11*R*-10t ... 72
Table S40. Cartesian Coordinates For The Optimized Conformer 7 Of Compound 3*R*,6*S*,11*R*-10t ... 73
Table S41. Boltzmann Distribution of Conformers of (3*R*,6*R*,11*S*)-10t ... 74
Table S42. Cartesian Coordinates For The Optimized Conformer 1 Of Compound 3*R*,6*R*,11*S*-10t ... 75
Table S43. Cartesian Coordinates For The Optimized Conformer 3 Of Compound 3*R*,6*R*,11*S*-10t ... 76
Table S44. Cartesian Coordinates For The Optimized Conformer 13 Of Compound 3*R*,6*R*,11*S*-10t ... 77
Table S45. Cartesian Coordinates For The Optimized Conformer 17 Of Compound 3*R*,6*R*,11*S*-10t ... 78
Table S46. Cartesian Coordinates For The Optimized Conformer 10 Of Compound 3*R*,6*R*,11*S*-10t ... 79
Table S47. Cartesian Coordinates For The Optimized Conformer 19 Of Compound 3*R*,6*R*,11*S*-10t ... 80
Table S48. Cartesian Coordinates For The Optimized Conformer 7 Of Compound 3*R*,6*R*,11*S*-10t ... 81
Table S49. Cartesian Coordinates For The Optimized Conformer 14 Of Compound 3*R*,6*R*,11*S*-10t ... 82
Table S50. Cartesian Coordinates For The Optimized Conformer 5 Of Compound 3*R*,6*R*,11*S*-10t ... 83

Table S51. Cartesian Coordinates For The Optimized Conformer 9 Of Compound 3*R*,6*R*,11*S*-10t 84
Table S52. Cartesian Coordinates For The Optimized Conformer 16 Of Compound 3*R*,6*R*,11*S*-10t 85
Table S53. Cartesian Coordinates For The Optimized Conformer 2 Of Compound 3*R*,6*R*,11*S*-10t 86
Table S54. Cartesian Coordinates For The Optimized Conformer 6 Of Compound 3*R*,6*R*,11*S*-10t 87
Table S55. Cartesian Coordinates For The Optimized Conformer 8 Of Compound 3*R*,6*R*,11*S*-10t 88
Table S56. Cartesian Coordinates For The Optimized Conformer 15 Of Compound 3*R*,6*R*,11*S*-10t 89

Details on known compounds

Ilimaquinone (1): yellow, crystalline solid; $[\alpha]^{22}_{D}$ -25 (*c* 1.12, $CHCl_3$); UVs (CH_3OH) λ_{max} (log ε) 286 (4.21), 214 (4.06) nm; ^{1}H NMR ($CDCl_3$, 500 MHz) *δ* 5.84 (1H, s), 4.44 (1H, brs), 4.42 (1H, brs), 3.86 (3H, s), 2.53 (1H, d, *J* = 13.7 Hz), 2.46 (1H, d, *J* = 13.7 Hz), 2.31 (1H, tdt, *J* = 13.8, 5.5, 1.8 Hz), 2.11-2.04 (2H, m), 1.86 (1H, m), 1.52-1.28 (5H, m), 1.20-1.10 (2H, m), 1.03 (3H, s), 0.97 (3H, d, *J* = 6.4 Hz), 0.84 (3H, s), 0.75 (1H, dd, *J* = 11.9, 2.2 Hz) ppm; ^{13}C NMR ($CDCl_3$, 125 MHz) *δ* 182.3, 182.0, 161.7, 160.5, 153.3, 117.3, 102.5, 102.0, 56.9, 50.1, 43.3, 40.4, 38.0, 36.6, 32.9, 32.3, 28.6, 27.9, 23.1, 20.5, 17.8, 17.4 ppm; HRESIMS *m/z* 359.2206 $[M+H]^+$ (calcd for $C_{22}H_{31}O_4$, 359.2217); HREIMS *m/z* 358.2128 (calcd for $C_{22}H_{30}O_4$, 358.2144). All spectroscopic data were consistent with literature reports [1-3].

5-*epi*-Ilimaquinone (2): yellow, amorphous solid; ^{1}H NMR ($CDCl_3$, 500 MHz) *δ* 5.87 (1H, s), 4.70 (1H, brs), 4.67 (1H, brs), 3.88 (3H, s), 2.59 (1H, d, *J* = 13.7 Hz), 2.49 (1H, d, *J* = 13.7 Hz), 2.46-2.40 (1H, m), 2.17-2.08 (2H, m), 2.03-1.99 (1H, m), 1.90-1.72 (2H, m), 1.22-1.06 (4H, m), 1.05 (3H, s), 0.94 (3H, d, *J* = 6.3 Hz), 0.88 (3H, s) ppm; HRESIMS *m/z* 359.2211 $[M+H]^+$ (calcd for $C_{22}H_{30}O_4$, 359.2217). ^{1}H NMR data were consistent with literature reports [3].

Smenospongine (3): red, amorphous solid; ^{1}H NMR ($CDCl_3$, 500 MHz) *δ* 5.64 (1H, s), 4.46 (1H, brs), 4.45 (1H, brs), 2.51 (1H, d, *J* = 13.9 Hz), 2.42 (1H, d, *J* = 13.9 Hz), 2.34 (1H, td, *J* = 13.8, 5.5 Hz), 2.13-2.06 (2H, m), 1.86 (1H, m), 1.54-1.35 (5H, m), 1.21-1.15 (2H, m), 1.06 (3H, s), 0.98 (3H, d, *J* = 6.4 Hz), 0.88 (3H, s), 0.79 (1H, dd, *J* = 11.9, 2.2 Hz) ppm; HRESIMS *m/z* 344.2210 $[M+H]^+$ (calcd for $C_{21}H_{30}NO_3$, 344.2226). ^{1}H NMR data were consistent with literature reports [2, 3].

Smenospongorine (4): red, amorphous solid; ^{1}H NMR ($CDCl_3$, 500 MHz) δ 6.52 (1H, brs), 5.36 (1H, s), 4.44 (1H, brs), 4.43 (1H, brs), 2.98 (2H, t, J = 6.5 Hz), 2.48 (1H, d, J = 13.8 Hz), 2.40 (1H, d, J = 13.8 Hz), 2.40-2.28 (2H, m), 2.12-1.82 (7H, m), 1.46-1.32 (2H, m), 1.04 (3H, s), 0.99 (3H, d, J = 6.7 Hz), 0.98 (3H, d, J = 6.7 Hz), 0.97 (3H, d, J = 6.4 Hz), 0.83 (3H, s), 0.78 (1H, dd, J = 11.8, 2.0 Hz) ppm; ^{13}C NMR ($CDCl_3$, 125 MHz) δ 182.8, 178.0, 160.5, 157.3, 150.3, 113.5, 102.5, 91.5, 49.8, 42.9, 41.2, 40.4, 37.8, 36.8, 36.6, 33.0, 32.4, 28.6, 27.9, 25.9, 23.2, 22.3, 20.5, 17.9, 17.3 ppm; HRESIMS m/z 400.2851 $[M+H]^+$ (calcd for $C_{25}H_{38}NO_3$, 400.2846). All data were consistent with literature reports [2].

Smenospongiarine (5): red, amorphous solid; ^{1}H NMR ($CDCl_3$, 500 MHz) δ 6.42 (1H, brs), 5.38 (1H, s), 4.46 (1H, brs), 4.45 (1H, brs), 3.18 (2H, td, J = 6.7, 6.5 Hz), 2.49 (1H, d, J = 13.9 Hz), 2.41 (1H, d, J = 14.0 Hz), 2.34 (1H, td, J = 13.8, 5.5 Hz), 2.13-2.06 (3H, m), 1.87 (1H, m), 1.78-1.12 (9H, m), 1.06 (3H, s), 0.99-0.93 (9H, overlapping d, d, d), 0.84 (3H, s), 0.79 (1H, dd, J = 11.8, 2.0 Hz) ppm; ^{13}C NMR ($CDCl_3$, 125 MHz) δ 182.8, 178.0, 160.5, 157.2, 150.3, 113.4, 102.5, 91.4, 49.8, 42.9, 41.2, 40.4, 37.8, 36.8, 36.6, 33.0, 32.4, 28.6, 27.9, 25.9, 23.2, 22.4, 22.3, 20.5, 17.9, 17.4 ppm; HRESIMS m/z 414.2999 $[M+H]^+$ (calcd for $C_{26}H_{40}NO_3$, 414.3003). All spectroscopic data were consistent with literature reports [2, 3].

Smenospongidine (6): red, amorphous solid; $[\alpha]^{22}_{D}$ +125 (c 0.1, $CHCl_3$); UVs (CH_3OH) λ_{max} (log ε) 488 (2.91), 323 (4.04), 248 (3.65) nm; IR (CaF_2 disc) ν_{max} 3269, 2922, 1642, 1589 and 1581 cm^{-1}; ^{1}H NMR ($CDCl_3$, 500 MHz) δ 8.35 (1H, brs), 7.33 (2H, t, J = 7.3 Hz), 7.26 (1H, m), 7.18 (2H, d, J = 7.0 Hz), 6.47 (1H, brs), 5.40 (1H, s), 4.45 (1H, brs), 4.44 (1H, brs), 3.42 (2H, q, J = 6.8 Hz), 2.95 (2H, t, J = 7.0 Hz), 2.49 (1H, d, J = 14.0 Hz), 2.39 (1H, d, J = 14.0 Hz), 2.32 (1H, m), 2.08 (1H, m), 2.06 (1H, m), 1.83 (1H, m), 1.51 (1H, m), 1.43 (1H, m), 1.38 (2H, m), 1.35 (1H, m), 1.18 (1H, m), 1.11 (1H, m), 1.06 (3H, s), 0.97 (3H, d, J = 6.5 Hz), 0.84 (3H, s), 0.77 (1H, dd, J = 11.9, 2.0 Hz) ppm; ^{13}C NMR ($CDCl_3$, 125 MHz) δ 182.7, 178.2, 160.5, 157.0, 150.0, 137.4, 128.9, 128.6, 127.0, 113.6, 102.5, 91.8, 49.8, 44.0, 42.9, 40.4, 37.8, 36.6, 34.2, 33.0, 32.4, 28.6, 27.9, 23.2, 20.5, 17.9, 17.3 ppm; HRESIMS m/z 448.2846 $[M+H]^+$ (calcd for $C_{29}H_{38}NO_3$, 448.2846).

Dictyoceratin A (7): white, amorphous solid; ^{1}H NMR ($CDCl_3$, 500 MHz) δ 7.50 (1H, d, J = 1.9 Hz), 7.39 (1H, d, J = 1.9 Hz), 6.56 (1H, brs), 5.98 (1H, brs), 4.41 (1H, brs), 4.36 (1H, brs), 3.86 (3H, s), 2.68 (1H, d, J = 14.3 Hz), 2.65 (1H, d, J = 14.4 Hz), 2.34 (1H, td, J = 13.8, 5.3 Hz), 2.09 (2H, m), 1.92

(1H, m), 1.57 (1H, qd, *J* = 13.2, 3.3 Hz), 1.50-1.19 (6H, m), 1.06 (3H, s), 1.03 (3H, d, *J* = 6.4 Hz), 0.95 (1H, dd, *J* = 12.1, 1.7 Hz), 0.87 (3H, s) ppm; ^{13}C NMR ($CDCl_3$, 125 MHz) *δ* 167.6, 160.1, 148.7, 142.3, 127.3, 125.1, 120.3, 113.9, 102.7, 52.0, 48.0, 42.1, 40.2, 36.9, 36.5, 36.3, 33.0, 27.8, 27.7, 23.1, 20.6, 17.6, 17.6 ppm; HRESIMS *m*/*z* 373.2375 $[M+H]^+$ (calcd for $C_{23}H_{33}O_4$, 373.2373). All spectroscopic data were consistent with literature reports [4].

Dictyoceratin B (8): white, amorphous solid; ^{1}H NMR ($CDCl_3$, 500 MHz) *δ* 10.71 (1H, brs), 7.14 (1H, s), 5.87 (1H, brs), 5.42 (1H, brs), 4.42 (1H, dd, *J* = 1.8, 1.8 Hz), 4.38 (1H, brs), 3.90 (3H, s), 2.60 (1H, d, *J* = 14.3 Hz), 2.57 (1H, d, *J* = 14.4 Hz), 2.34 (1H, td, *J* = 13.9, 5.4 Hz), 2.08 (2H, m), 1.90 (1H, m), 1.59-1.50 (1H, m), 1.47 (1H, dt, *J* = 12.4, 3.2 Hz), 1.42-1.20 (5H, m), 1.06 (3H, s), 1.00 (3H, d, *J* = 6.4 Hz), 0.94 (1H, dd, *J* = 11.9, 2.1 Hz), 0.84 (3H, s) ppm; ^{13}C NMR ($CDCl_3$, 125 MHz) *δ* 170.6, 160.2, 148.9, 147.0, 130.3, 124.6, 117.1, 104.4, 102.7, 52.0, 47.8, 41.8, 40.1, 36.6, 36.2, 36.2, 33.1, 27.8, 27.7, 23.0, 20.6, 17.6, 17.5 ppm; HRESIMS *m*/*z* 411.2142 $[M+Na]^+$ (calcd for $C_{23}H_{32}O_5Na$, 411.2142). All spectroscopic data were consistent with literature reports [4].

Dictyoceratin C (9): white, amorphous solid; ^{1}H NMR ($CDCl_3$, 500 MHz) *δ* 7.76 (2H, m), 6.73 (1H, d, *J* = 9.0 Hz), 5.22 (1H, brs), 4.41 (1H, dd, *J* = 1.8, 1.8 Hz), 4.36 (1H, brs), 3.86 (3H, s), 2.67 (1H, d, *J* = 14.5 Hz), 2.62 (1H, d, *J* = 14.3 Hz), 2.34 (1H, td, *J* = 13.6, 5.4 Hz), 2.08 (2H, m), 1.93 (1H, m), 1.59-1.17 (7H, m), 1.06 (3H, s), 1.03 (3H, d, *J* = 6.5 Hz), 0.95 (1H, dd, *J* = 12.0, 2.0 Hz), 0.88 (3H, s) ppm; HRESIMS *m*/*z* 357.2423 $[M+H]^+$ (calcd for $C_{23}H_{33}O_3$, 357.2424). ^{1}H NMR data was consistent with literature reports [5].

In replicating the one-step synthesis of smenospongidine from ilimaquinone and phenethylamine which was required to confirm the structure given the conflicting and missing NMR data in the literature [6], we also discovered smenospongidinimine (**11)**, a previously unreported side product . Smenospongidinimine, which was produced in a 3:5 ratio with smenospongidine because of the presence of excess phenethylamine, displays symmetry in the quinone ring of the molecule because of the equilibrium between two rapidly interconverting tautomers (See below). Compound **11** had an CC_{50} value of 19.3 μM against U251MG, the only cell line it was tested against.

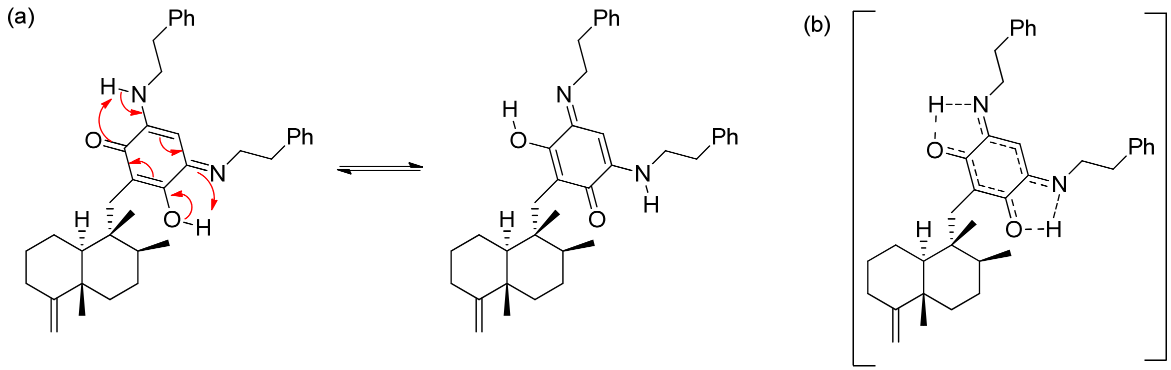

(a) The two tautomeric forms and (b) the resonance hybrid of smenospongidinimine (**11**)

Synthesis of smenospongidine (6) from ilimaquinone (1). This protocol was adapted from Ling *et al* [6]. To a solution of 13 mg (36 μmol, 1 equiv.) of ilimaquinone (**1**) in 6.5 mL methanol in a round bottomed flask was added a solution of 1.3 mL methanol containing 7 μL (54 μmol, 1.5 equiv.) of phenethylamine, followed by 6 mg $NaHCO_3$. The flask was stirred for 10 h at 40 °C. After cooling the purple-colored reaction mixture to room temperature, a few drops of 1N HCl were added to slightly acidify the solution, which turned red. The solvent was removed *in vacuo* and the crude mixture was dissolved in 4 mL *tert*-butyl methyl ether and extracted with 4 mL of water. The organic layer was separated and after removal of solvent *in vacuo*, 16.3 mg of a crude red solid was obtained. Purification by HPLC (Silica, 250 x 10 mm, 3.0 mL/min flow, elution with ethyl acetate/hexane using the following gradient: 15% – 30% ethyl acetate over 15 min, held at that composition for 10 min, followed by a wash with ethyl acetate for an additional 10 min) afforded synthetic smenospongidine (**6**, t_R 11.7 min, 8.6 mg, 52.8% yield, >95% purity by ^{1}H NMR) and smenospongidinimine (**11**, t_R 31.0 min, 5.3 mg, 26.7% yield, >95% purity by ^{1}H NMR).

Smenospongidinimine (11): blue, amorphous solid; $[\alpha]^{22}_D$ +126 (*c* 0.1, $CHCl_3$); UVs (CH_3OH) λ_{max} (log ε) 354 (4.33), 254 (3.91) nm; IR (CaF_2 disc) ν_{max} 3185, 2923, 2857, 1635 and 1591 cm^{-1}; ^{1}H NMR ($CDCl_3$, 500 MHz) δ 8.40 (2H, brs), 7.32 (4H, t, J = 7.7 Hz), 7.25 (2H, m), 7.18 (4H, d, J = 7.9

Hz), 4.79 (1H, s), 4.44 (1H, brs), 4.43 (1H, brs), 3.51 (4H, t, *J* = 7.3 Hz), 2.95 (4H, t, *J* = 7.3 Hz), 2.45 (1H, d, *J* = 13.8 Hz), 2.40 (1H, d, *J* = 13.7 Hz), 2.32 (2H, m), 2.05 (1H, m), 1.83 (1H, m), 1.50 (1H, m), 1.42-1.10 (6H, m), 1.04 (3H, s), 1.03 (3H, d, *J* = 6.7 Hz), 0.88 (1H, dd, *J* = 11.8, 2.2 Hz), 0.80 (3H, s) ppm; ^{13}C NMR ($CDCl_3$, 125 MHz) δ 171.2, 161.8, 156.6, 137.2, 129.0, 128.6, 127.1, 109.1, 101.6, 80.1, 49.7, 44.6, 42.6, 40.5, 37.8, 37.0, 34.8, 33.4, 33.2, 29.0, 28.1, 23.4, 20.7, 18.1, 17.4 ppm; HRESIMS *m/z* 551.3635 $[M+H]^+$ (calcd for $C_{37}H_{47}N_2O_2$, 551.3632).

1. Luibrand, R. T.; Erdman, T. R.; Vollmer, J. J.; Scheuer, P. J.; Finer, J.; Clardy, J., Ilimaquinone, a sesquiterpenoid quinone from a marine sponge. *Tetrahedron* **1979**, 35, 609-612.
2. Kondracki, M.-L.; Guyot, M., Biologically active quinone and hydroquinone sesquiterpenoids from the sponge *Smenospongia* sp. *Tetrahedron* **1989**, 45, 1995-2004.
3. Rodríguez, J.; Quiñoá, E.; Riguera, R.; Peters, B. M.; Abrell, L. M.; Crews, P., The structures and stereochemistry of cytotoxic sesquiterpene quinones from *Dactylospongia elegans*. *Tetrahedron* **1992**, 48, 6667-6680.
4. Nakamura, H.; Deng, S.; Kobayashi, J. i.; Ohizumi, Y.; Hirata, Y., Dictyoceratin-a and -b, novel antimicrobial terpenoids from the okinawan marine sponge *Hipposponqia* sp. *Tetrahedron* **1986**, 42, 4197-4201.
5. Kwak, J. H.; Schmitz, F. J.; Kelly, M., Sesquiterpene quinols/quinones from the micronesian sponge *Petrosaspongia metachromia*. *J. Nat. Prod.* **2000**, 63, 1153-1156.
6. Ling, T.; Poupon, E.; Rueden, E. J.; Kim, S. H.; Theodorakis, E. A., Unified synthesis of quinone sesquiterpenes based on a radical decarboxylation and quinone addition reaction. *J. Am. Chem. Soc.* **2002**, 124, 12261-12267.

Figure S1. Biological Evaluation of Compounds 1-10 in U251MG and BACE1 Assays

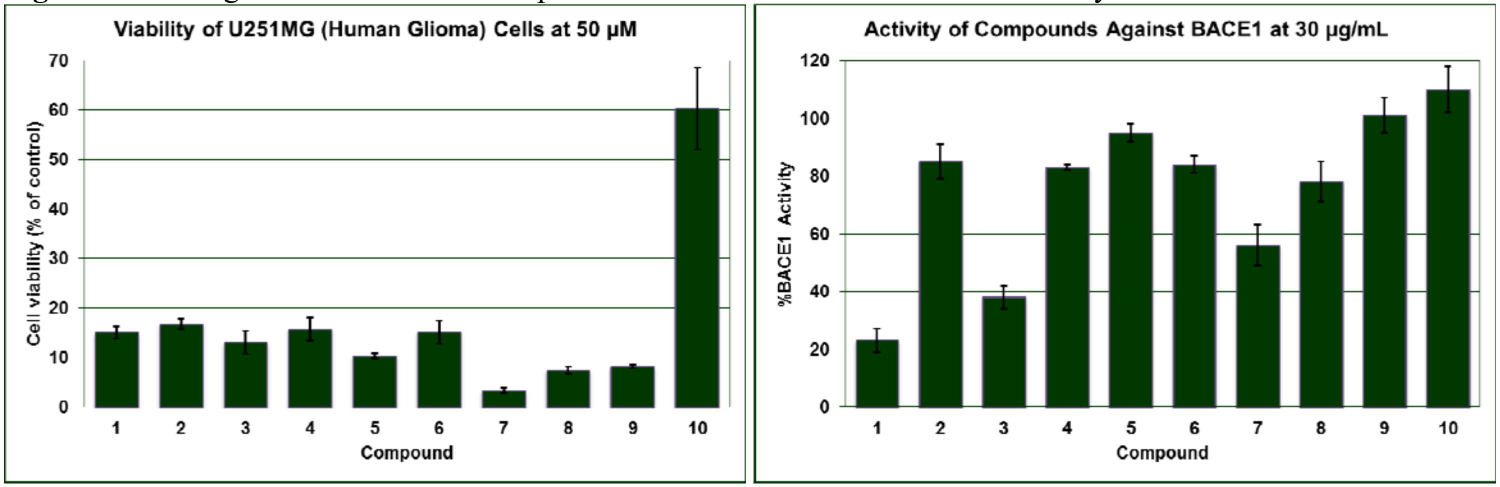

Table S1. Biological evaluation data

Compound	*% BACE-1 activity (std. dev.) at 30 μg/mL*	*% viability of U251MG cells (std. dev.) at 50 μM*
1	23 (4)	15 (1)
2	85 (6)	17 (1)
3	38 (4)	13 (2)
4	83 (1)	16 (2)
5	95 (3)	10 (1)
6	84 (3)	15 (2)
7	56 (7)	3 (1)
8	78 (7)	7 (1)
9	101 (6)	8 (0)
10	110 (8)	60 (8)

Figure S2. Cytotoxicity Curves of Selected Compounds in Panc-1 Assay

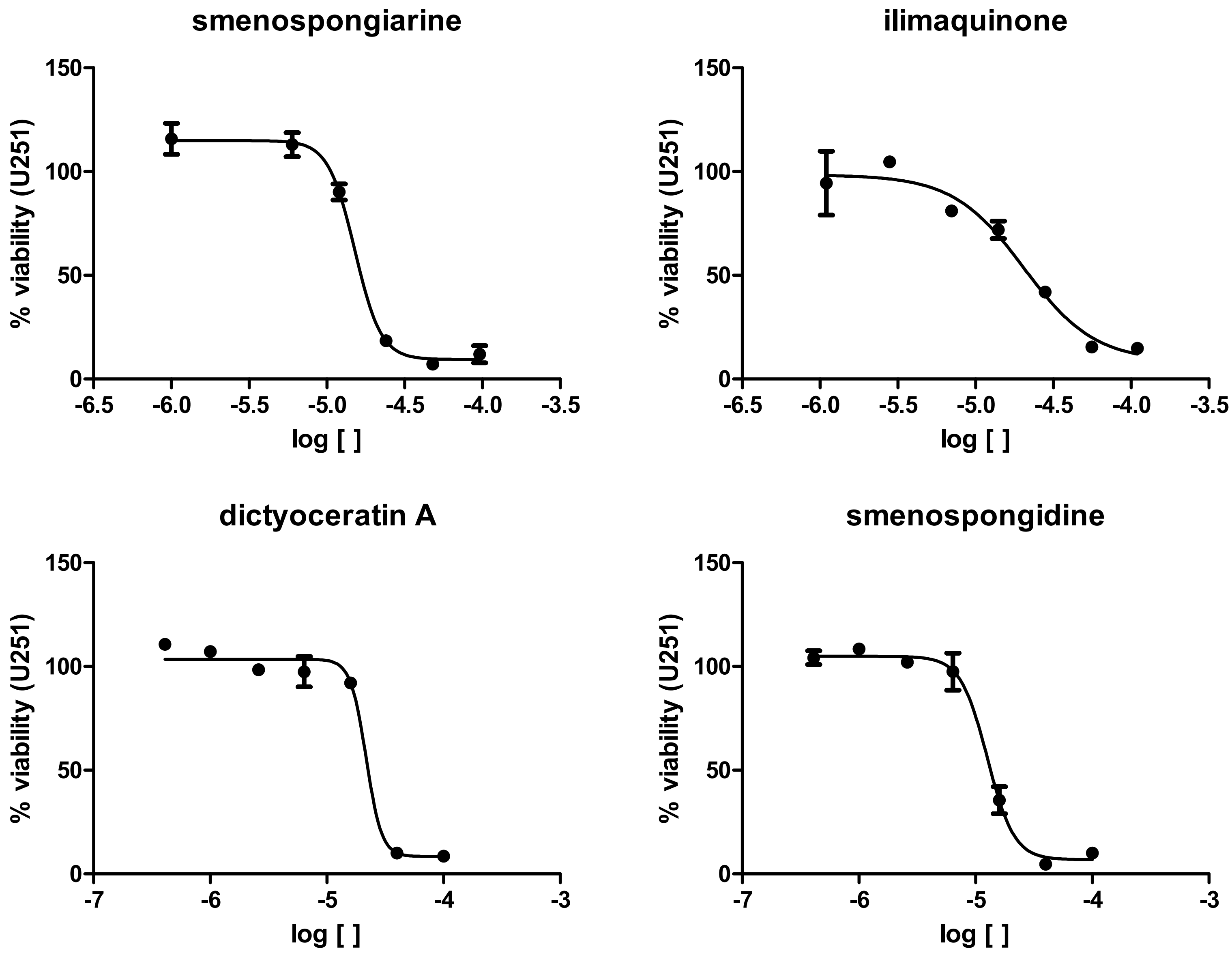

Table S2. Summary of Experimental and Computed ^{1}H and ^{13}C NMR shifts of all possible diastereomers of 10

		Computed								
**Proton**	**Experimental**	**SRR**	**SSR**	**SRS**	**SSS**		**|δSRR-δExp|**	**|δSSR-δExp|**	**|δSRS-δExp|**	**|δSSS-δExp|**
15	5.83	5.73	6.04	5.94	5.86		0.10	0.21	0.11	0.03
11	4.98	5.13	5.31	4.60	4.82		0.15	0.33	0.38	0.16
10a	2.36	1.98	2.10	2.19	2.43		0.38	0.26	0.17	0.07
10b	2.28	2.79	2.59	2.39	2.18		0.51	0.31	0.11	0.10
8a	1.86	2.12	1.82	1.81	1.98		0.26	0.04	0.05	0.12
8b	2.50	2.68	2.77	2.73	2.52		0.18	0.27	0.23	0.02
7a	2.07	1.74	1.88	1.91	2.12		0.33	0.19	0.16	0.05
7b	1.68	1.54	1.50	1.54	1.67		0.14	0.18	0.14	0.01
6	3.11	2.78	2.15	2.72	3.07		0.33	0.96	0.39	0.04
3	5.49	3.42	4.20	4.38	5.36		2.07	1.29	1.11	0.13
16	1.14	1.01	1.14	1.13	1.09		0.13	0.00	0.01	0.05
17	2.84	3.05	3.15	2.80	2.74		0.21	0.31	0.04	0.10
						**ABS[Δδ]=**	4.78	4.34	2.90	0.89
						**MAE=**	0.40	0.36	0.24	0.07

**Carbon**	**Experimental**	**SRR**	**SSR**	**SRS**	**SSS**		**|δSRR-δExp|**	**|δSSR-δExp|**	**|δSRS-δExp|**	**|δSSS-δExp|**
9	138.9	145.1	142.0	145.2	144.1		6.2	3.1	6.3	5.2
8	26.1	26.7	28.7	29.3	26.9		0.6	2.6	3.2	0.8
7	26.5	32.1	31.6	29.8	26.2		5.6	5.1	3.3	0.3
6	36.0	38.1	37.0	37.5	37.1		2.1	1.0	1.5	1.1
5	177.4	174.8	174.8	173.1	174.3		2.6	2.6	4.3	3.1
3	55.7	60.1	57.0	56.6	52.9		4.4	1.3	0.9	2.8
2	171.1	170.4	170.7	172.0	171.4		0.7	0.4	0.9	0.3
11	73.5	68.0	69.4	74.4	69.9		5.5	4.1	0.9	3.6
10	40.2	39.1	39.3	42.4	42.2		1.1	0.9	2.2	2.0
15	116.7	116.5	121.2	119.5	119.3		0.2	4.5	2.8	2.6
16	14.3	15.4	15.7	15.6	12.7		1.1	1.4	1.3	1.6
17	30.3	34.6	30.8	27.4	28.8		4.3	0.5	2.9	1.5
						**ABS[Δδ]=**	34.3	27.5	30.4	24.9
						**MAE=**	2.9	2.3	2.5	2.1

Figure S3. ^{1}H NMR Spectrum (500 MHz) of **1** in $CDCl_3$

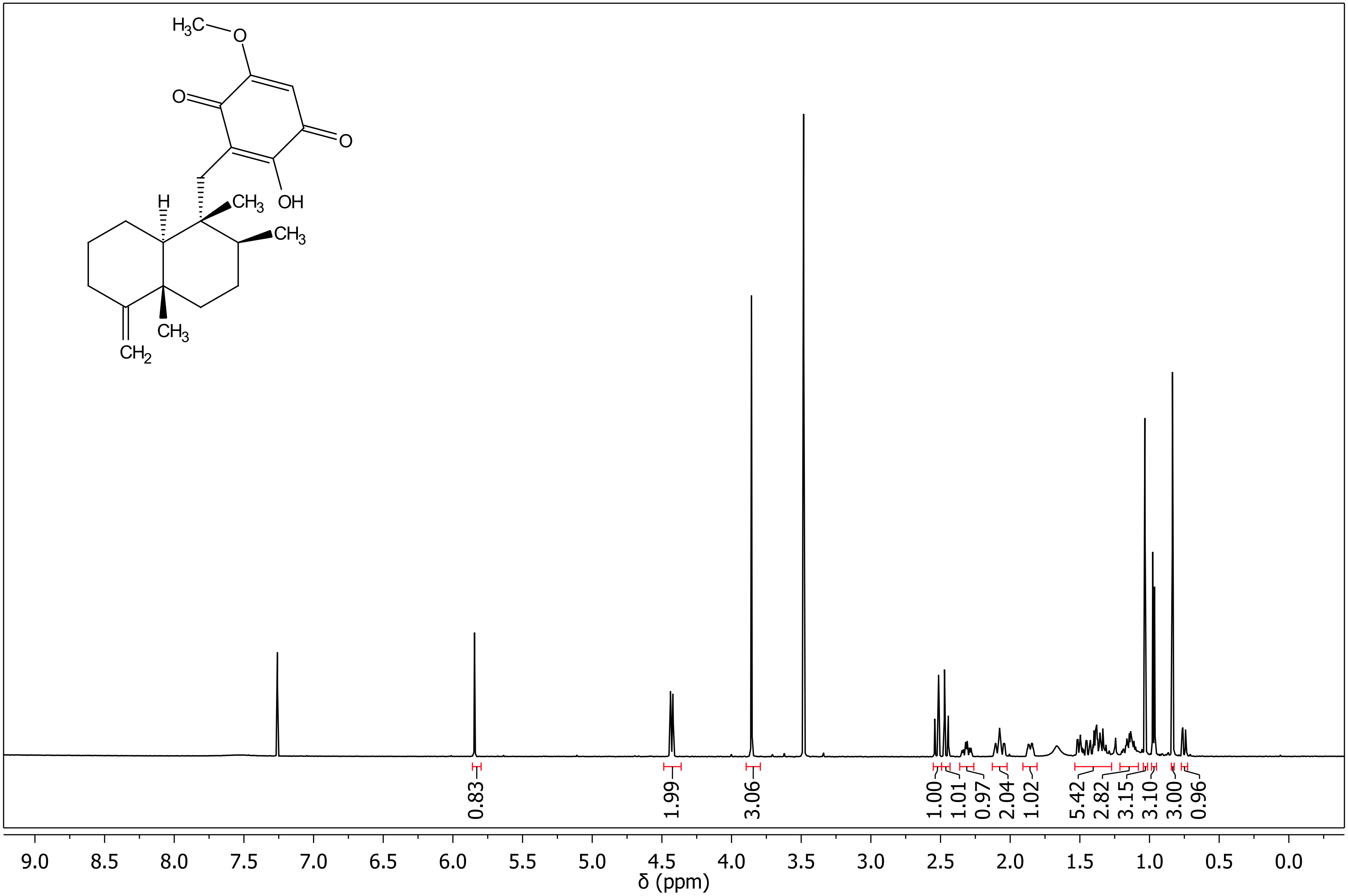

Figure S4. ^{13}C NMR Spectrum (125 MHz) of **1** in $CDCl_3$

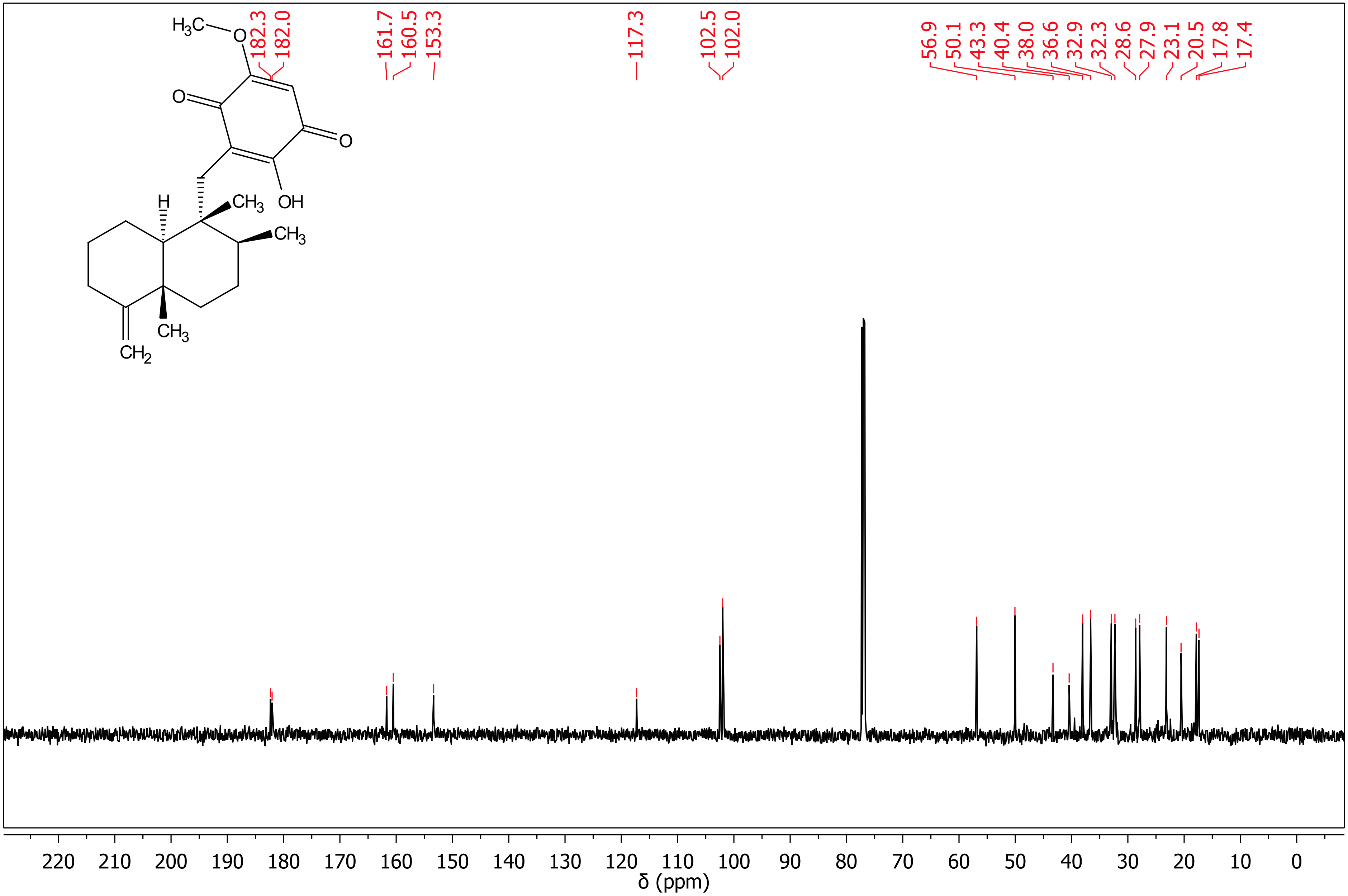

Figure S5. ^{1}H NMR Spectrum (500 MHz) of **2** in $CDCl_3$

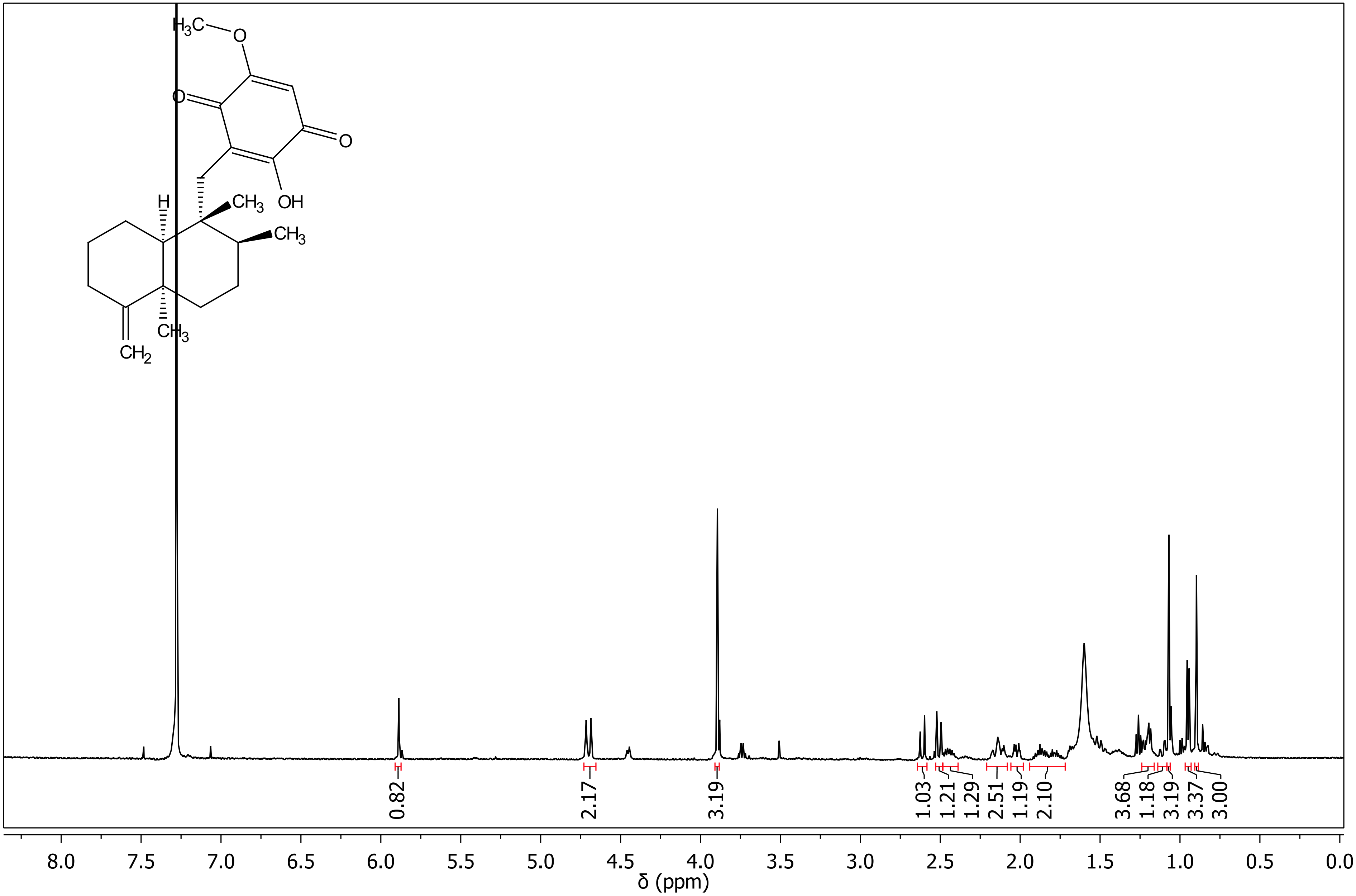

Figure S6. ^{1}H NMR Spectrum (500 MHz) of **3** in $CDCl_3$

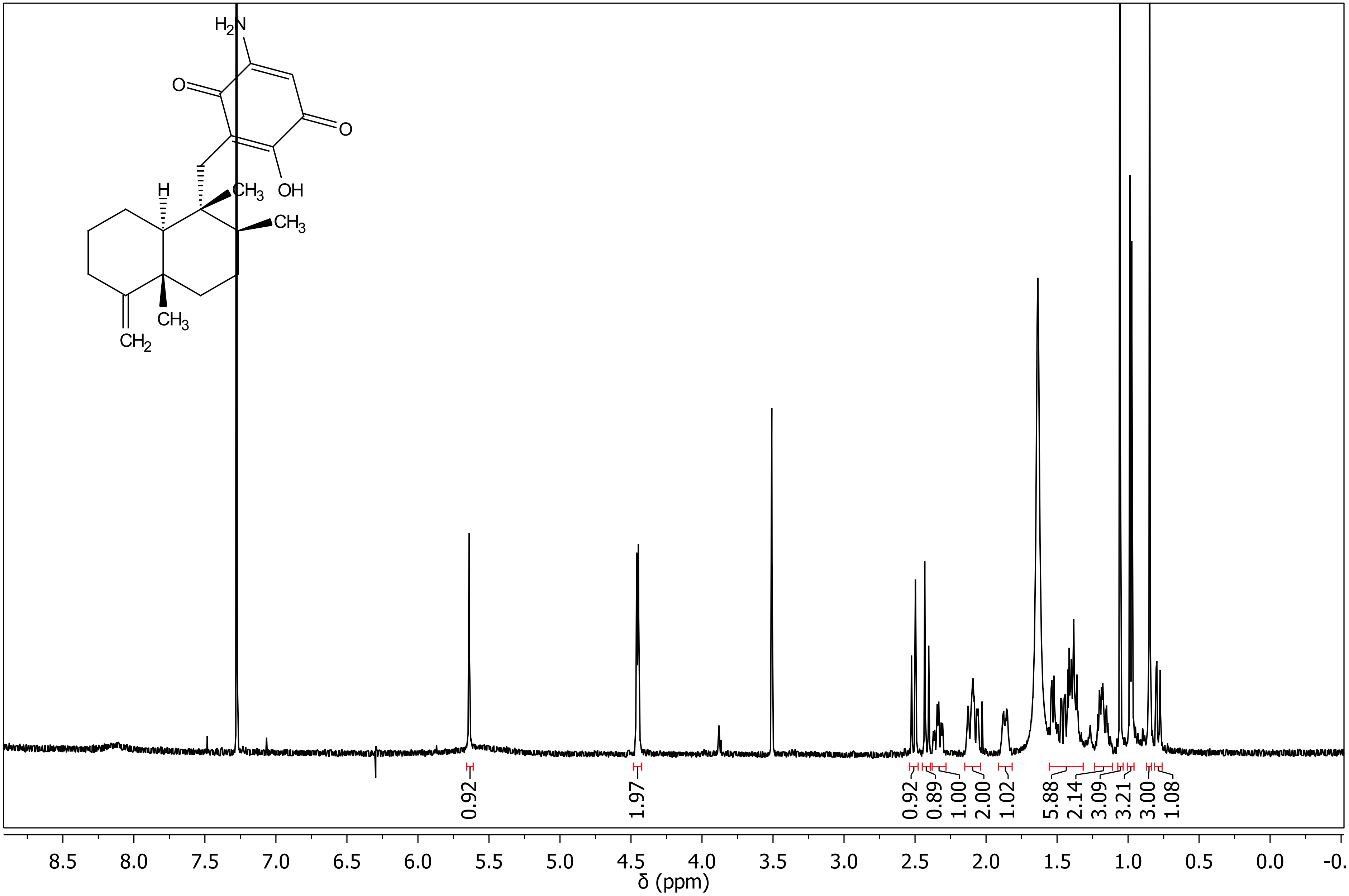

Figure S7. ^{1}H NMR Spectrum (500 MHz) of **4** in $CDCl_3$

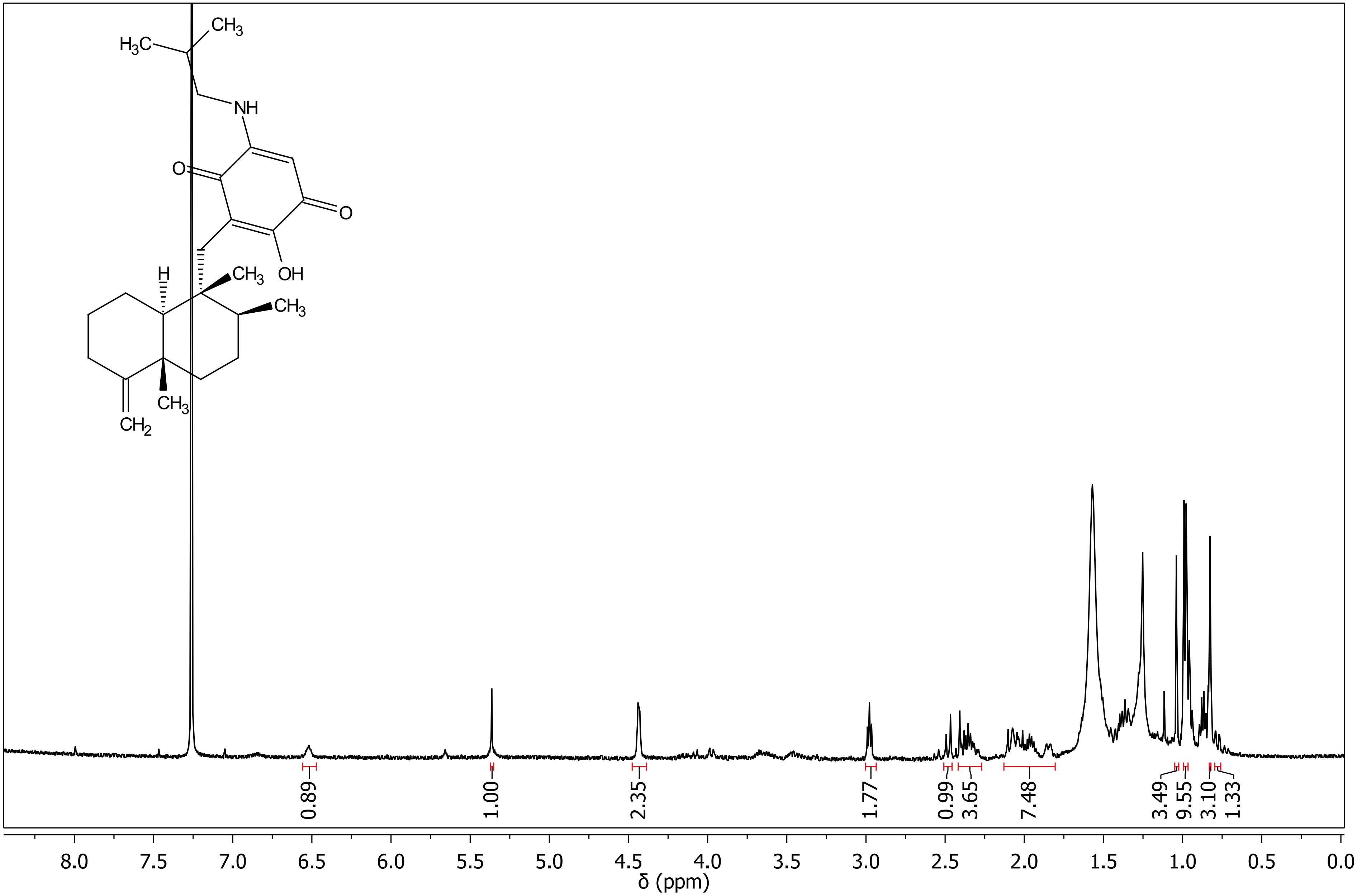

Figure S8. ^{13}C NMR Spectrum (125 MHz) of **4** in $CDCl_3$

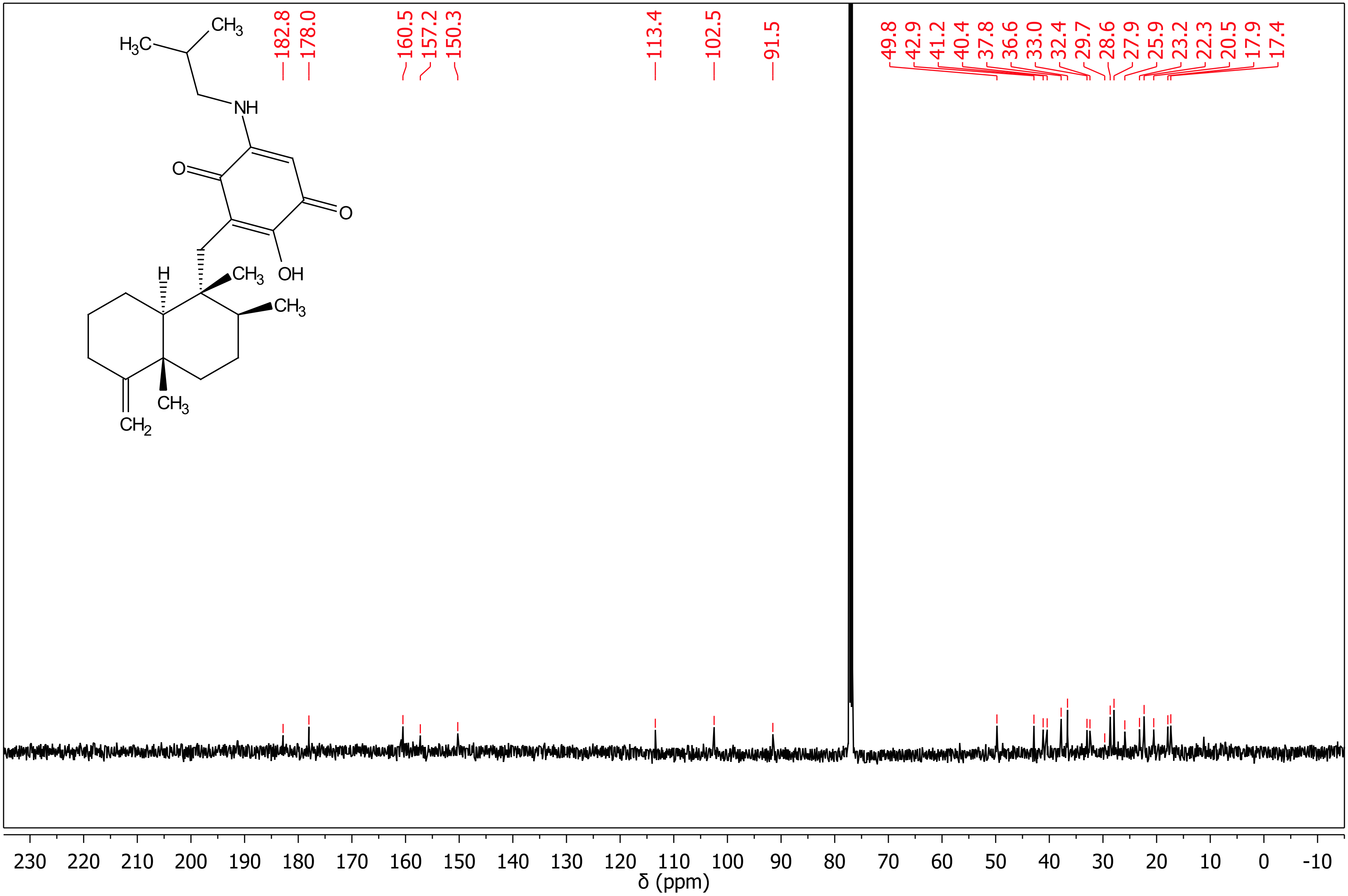

Figure S9. ^{1}H NMR Spectrum (500 MHz) of **5** in $CDCl_3$

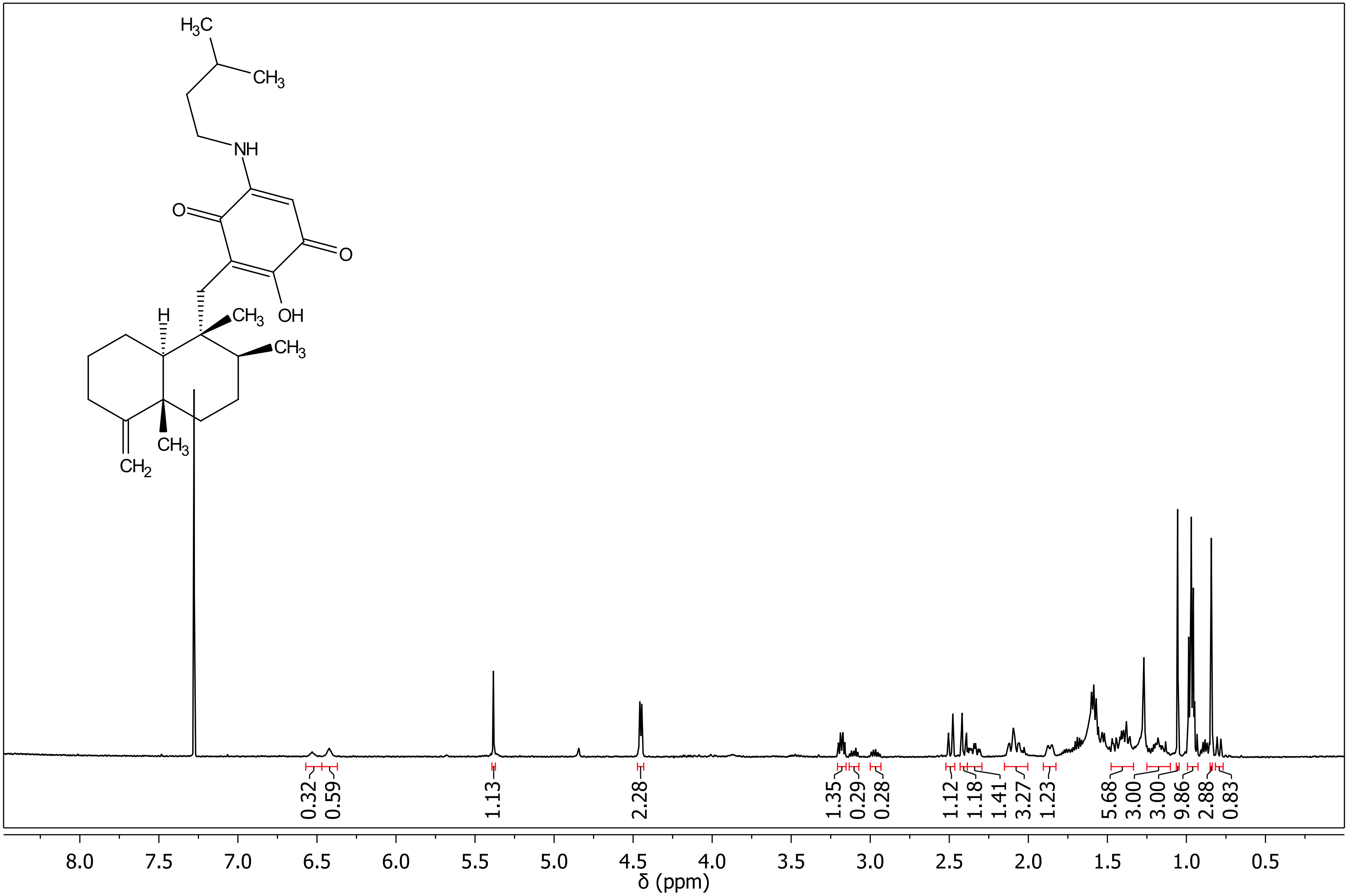

Figure S10. ^{13}C NMR Spectrum (125 MHz) of **5** in C*D*Cl$_3$

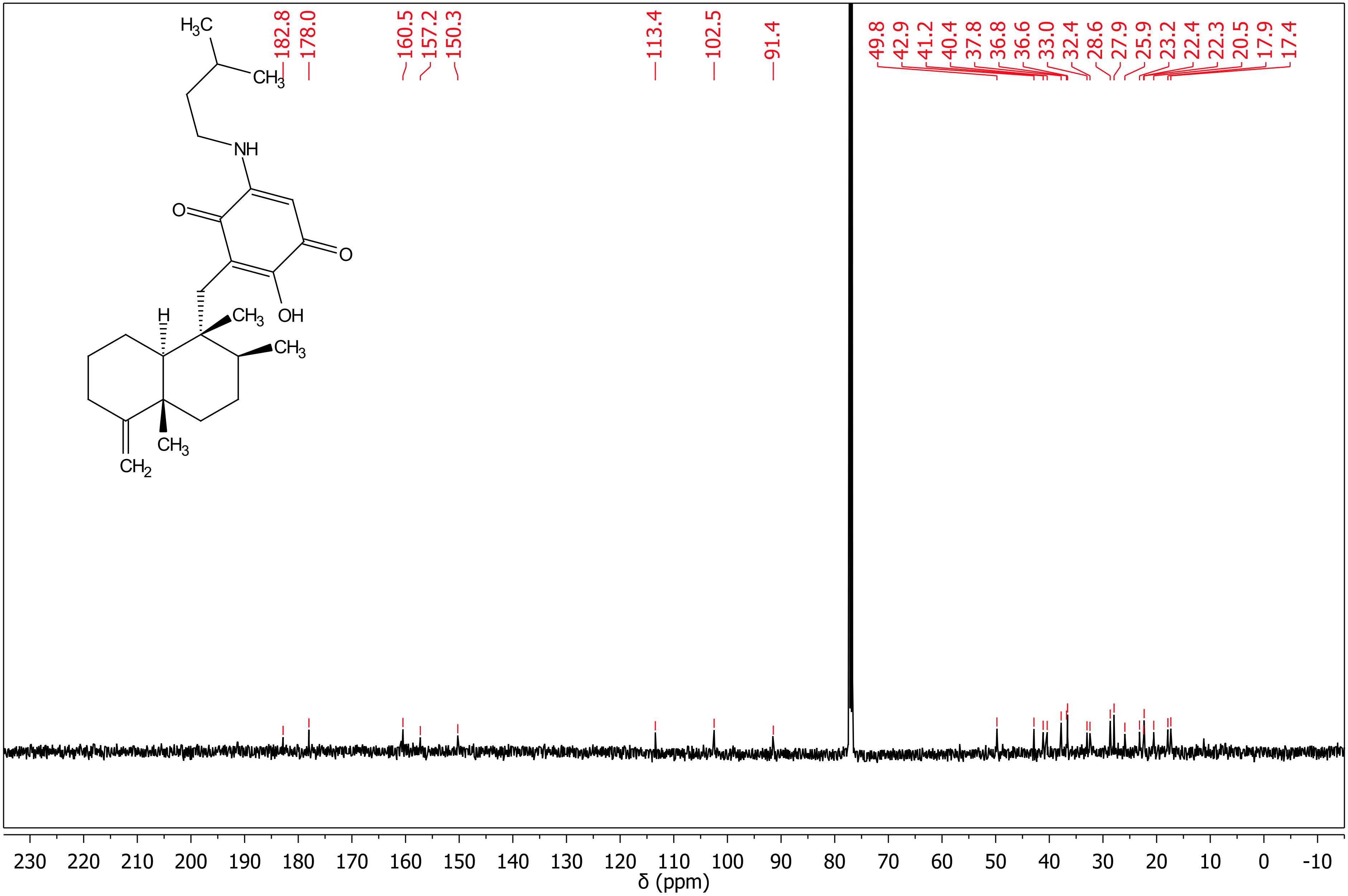

Figure S11. ^{1}H NMR Spectrum (500 MHz) of **6** in $CDCl_3$

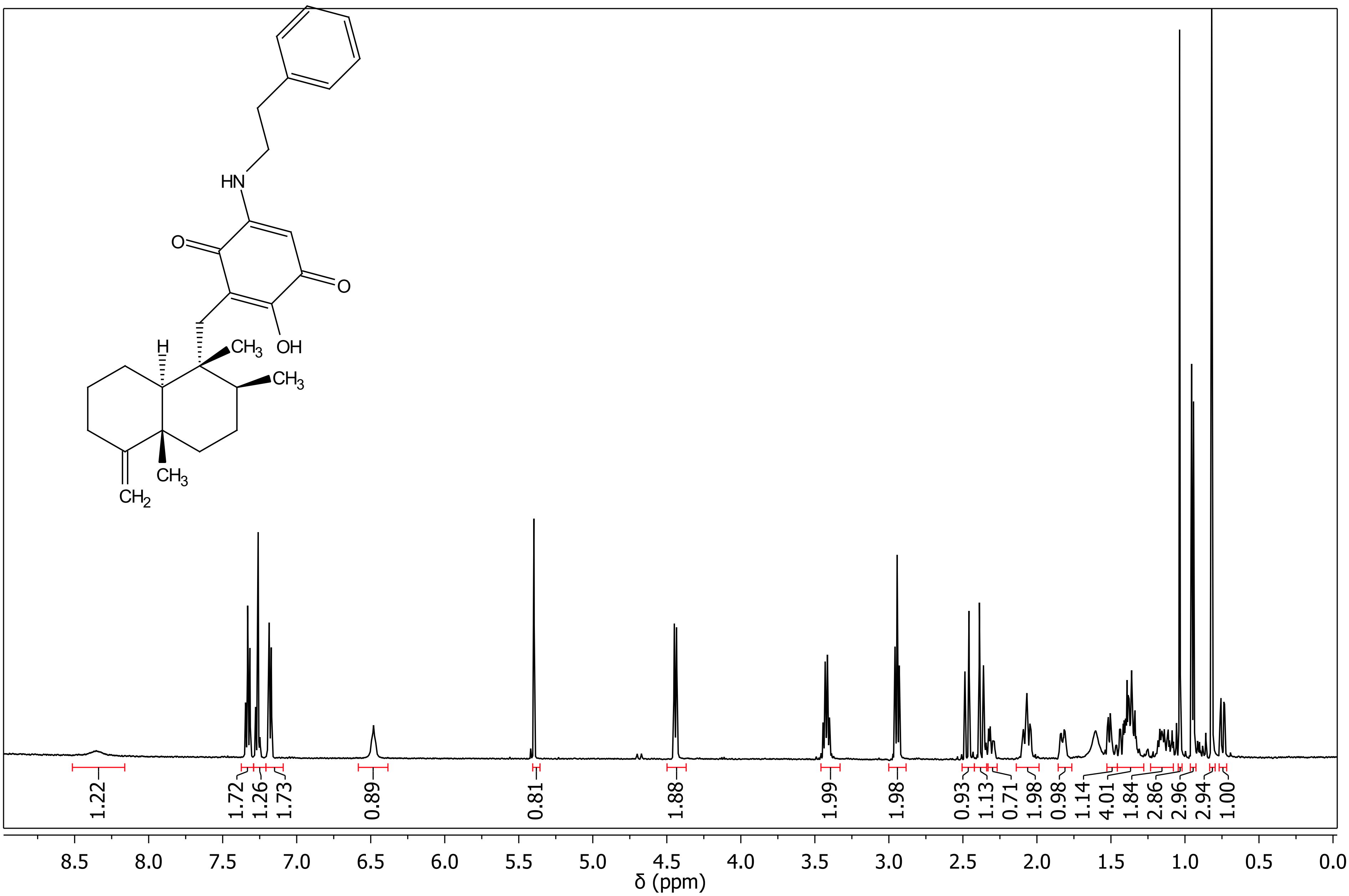

Figure S12. ^{13}C NMR Spectrum (125 MHz) of **6** in $CDCl_3$

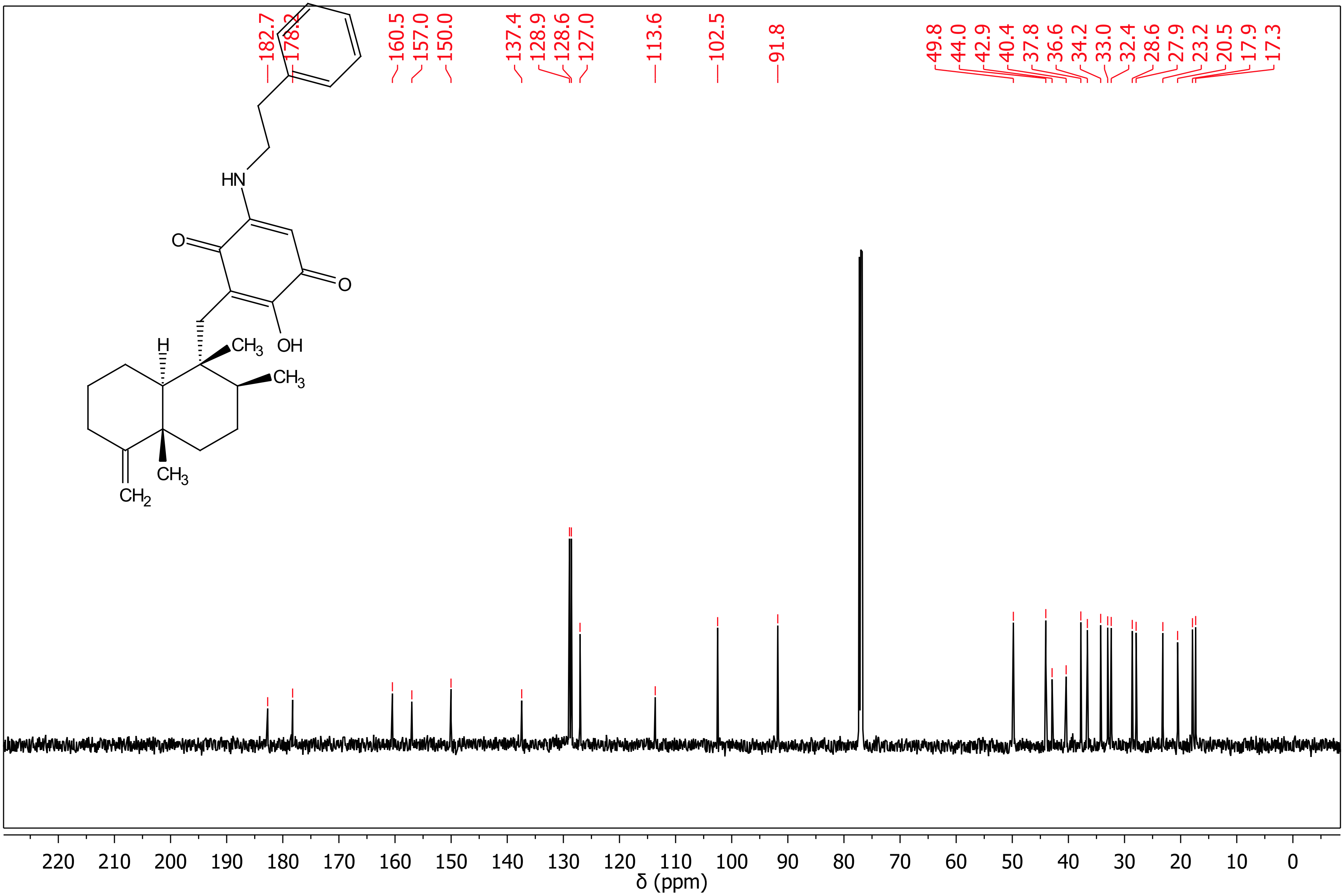

Figure S13. ^{1}H NMR Spectrum (500 MHz) of **7** in $CDCl_3$

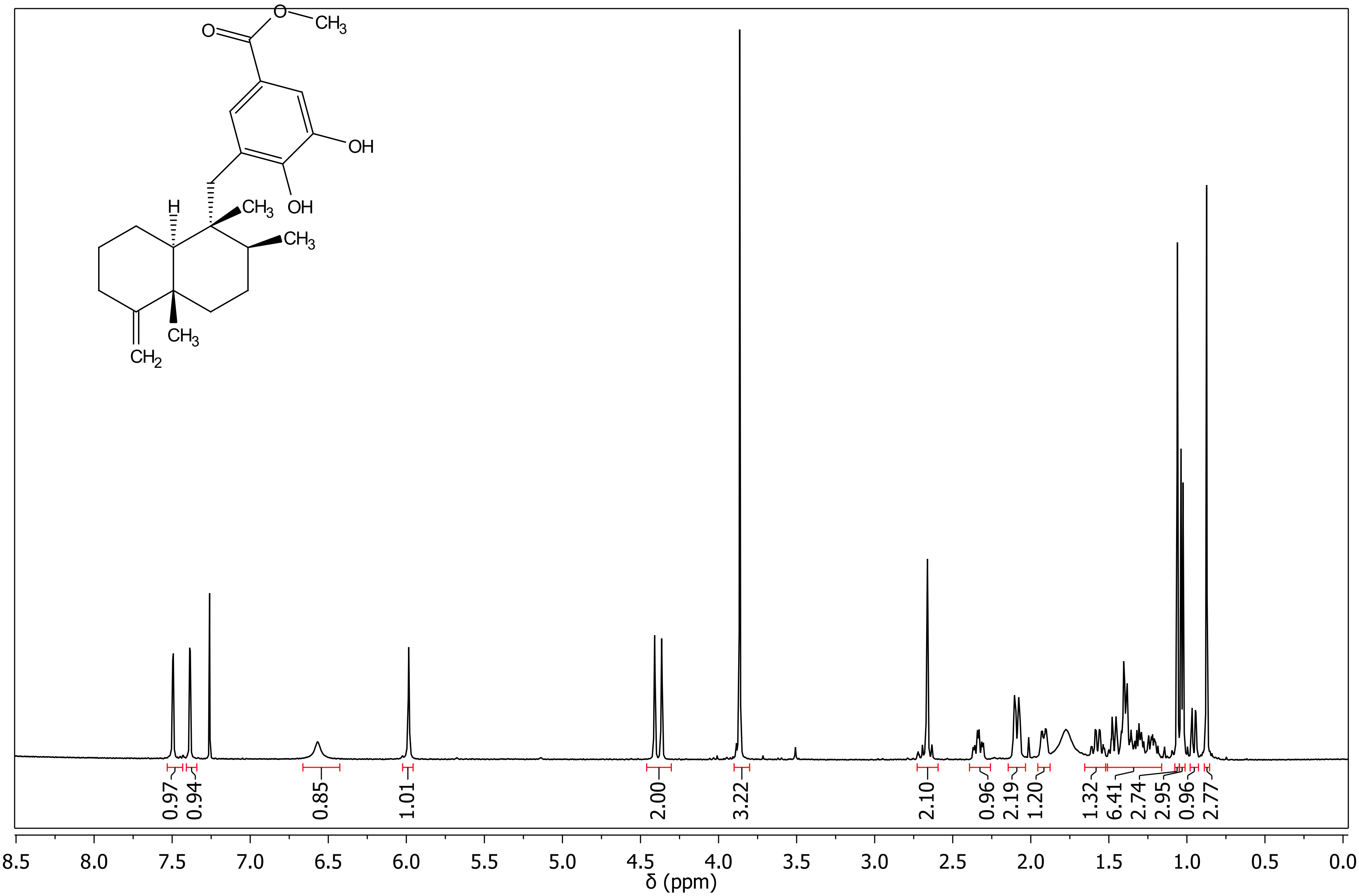

Figure S14. ^{13}C NMR Spectrum (125 MHz) of **7** in $CDCl_3$

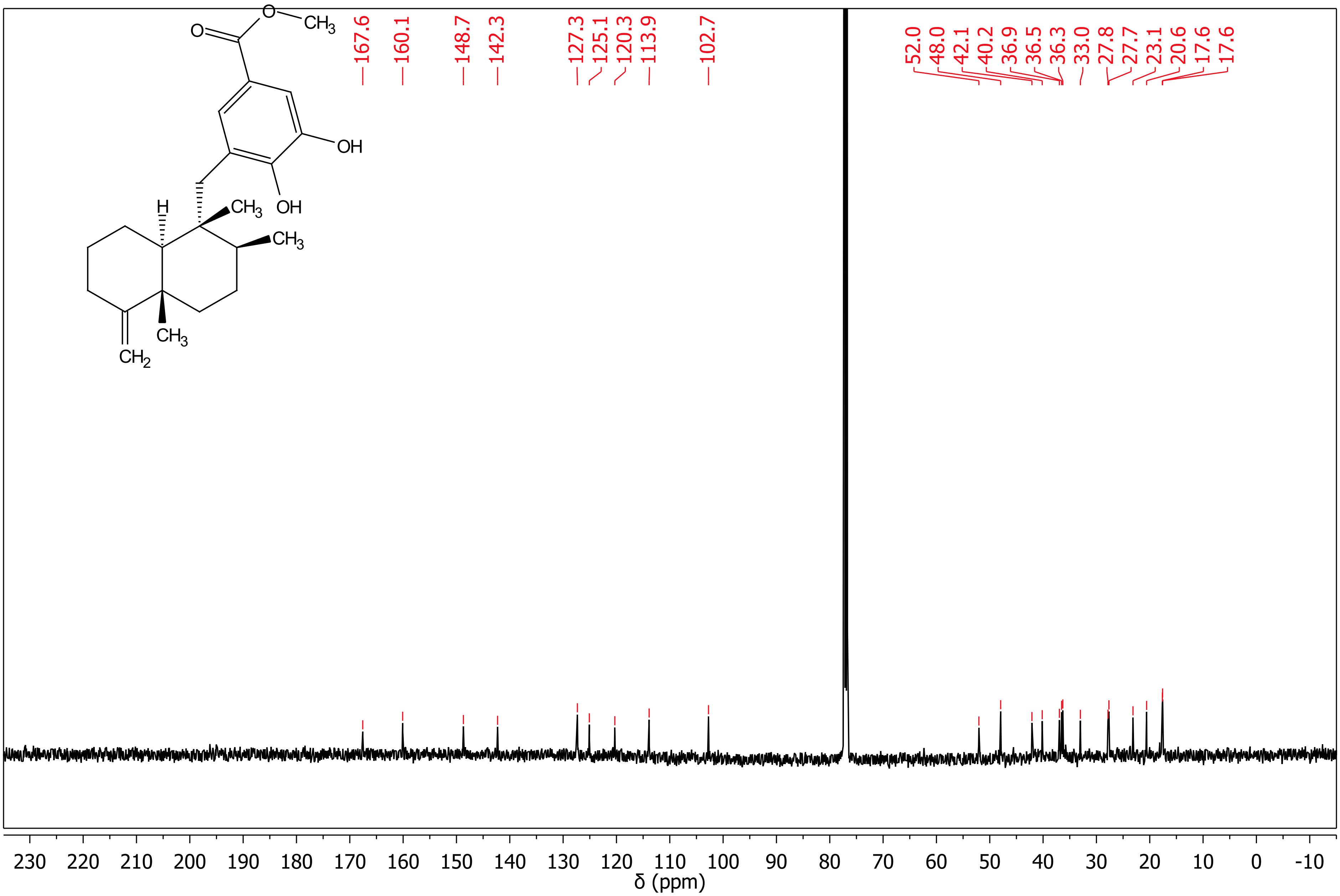

Figure S15. ^{1}H NMR Spectrum (500 MHz) of **8** in $CDCl_3$

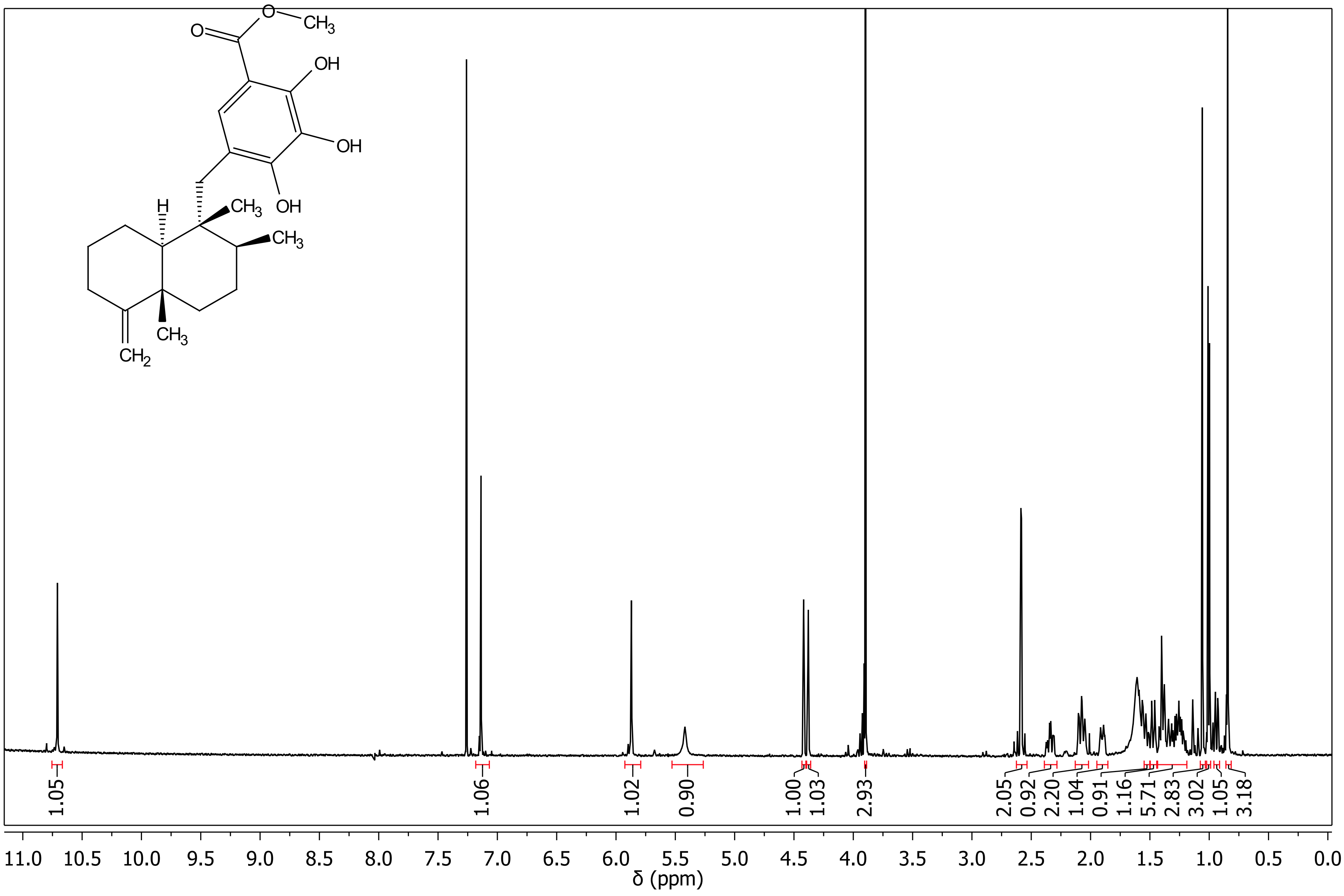

Figure S16. ^{13}C NMR Spectrum (125 MHz) of **8** in $CDCl_3$

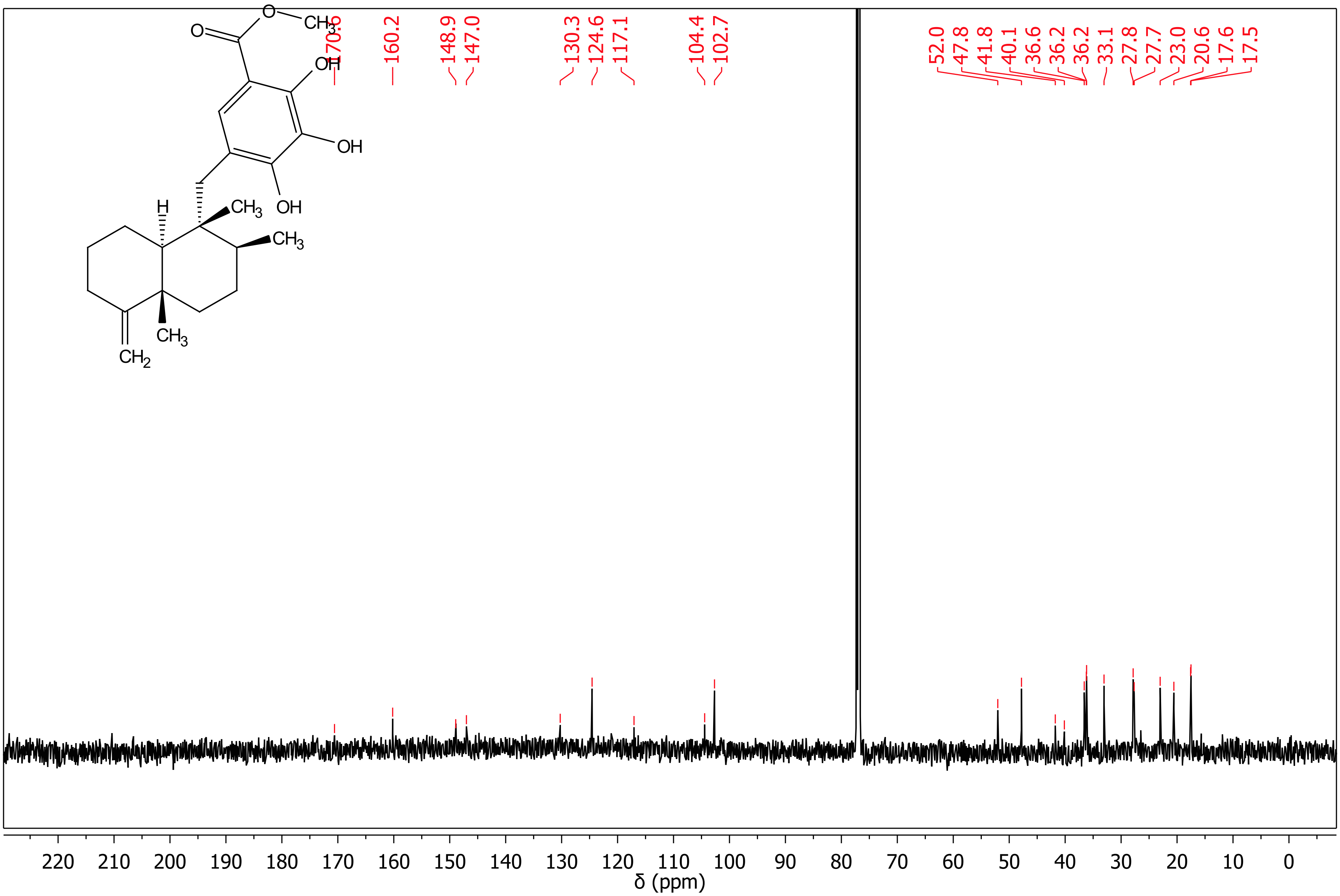

Figure S17. ^{1}H NMR Spectrum (500 MHz) of **9** in $CDCl_3$

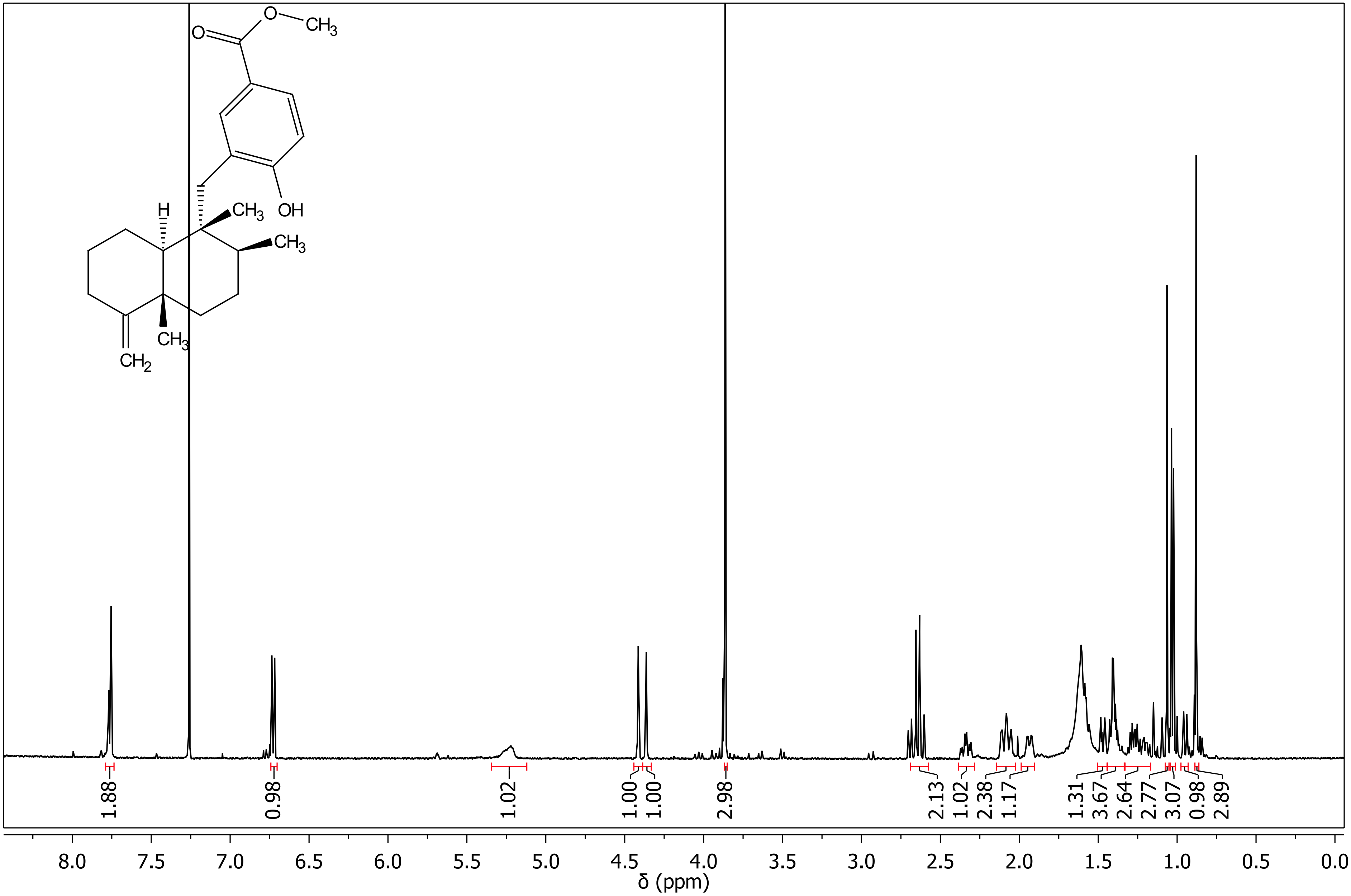

Figure S18. ^{1}H NMR Spectrum (500 MHz) of **10** in $CDCl_3$

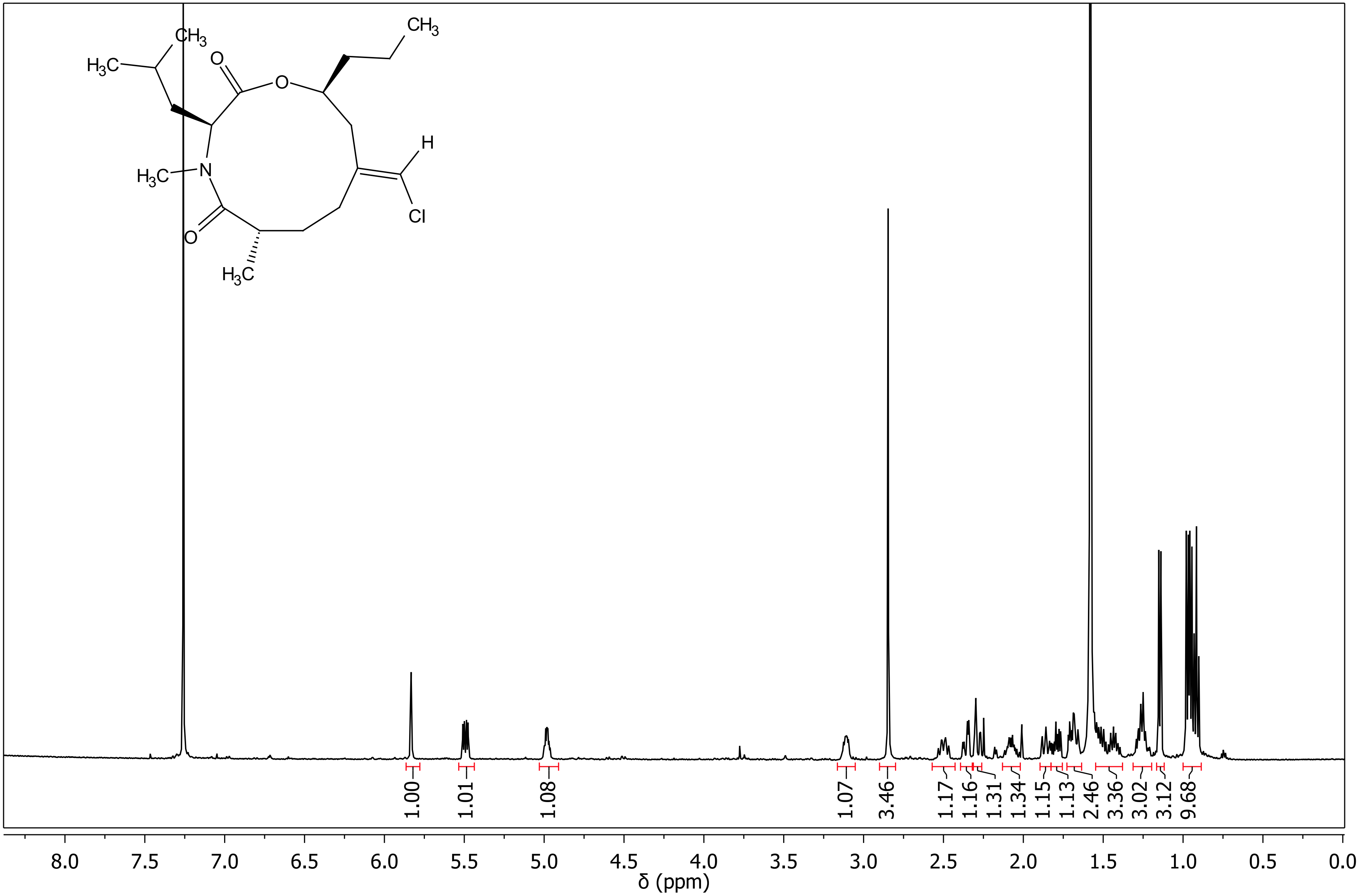

Figure S19. ^{13}C NMR Spectrum (125 MHz) of **10** in $CDCl_3$

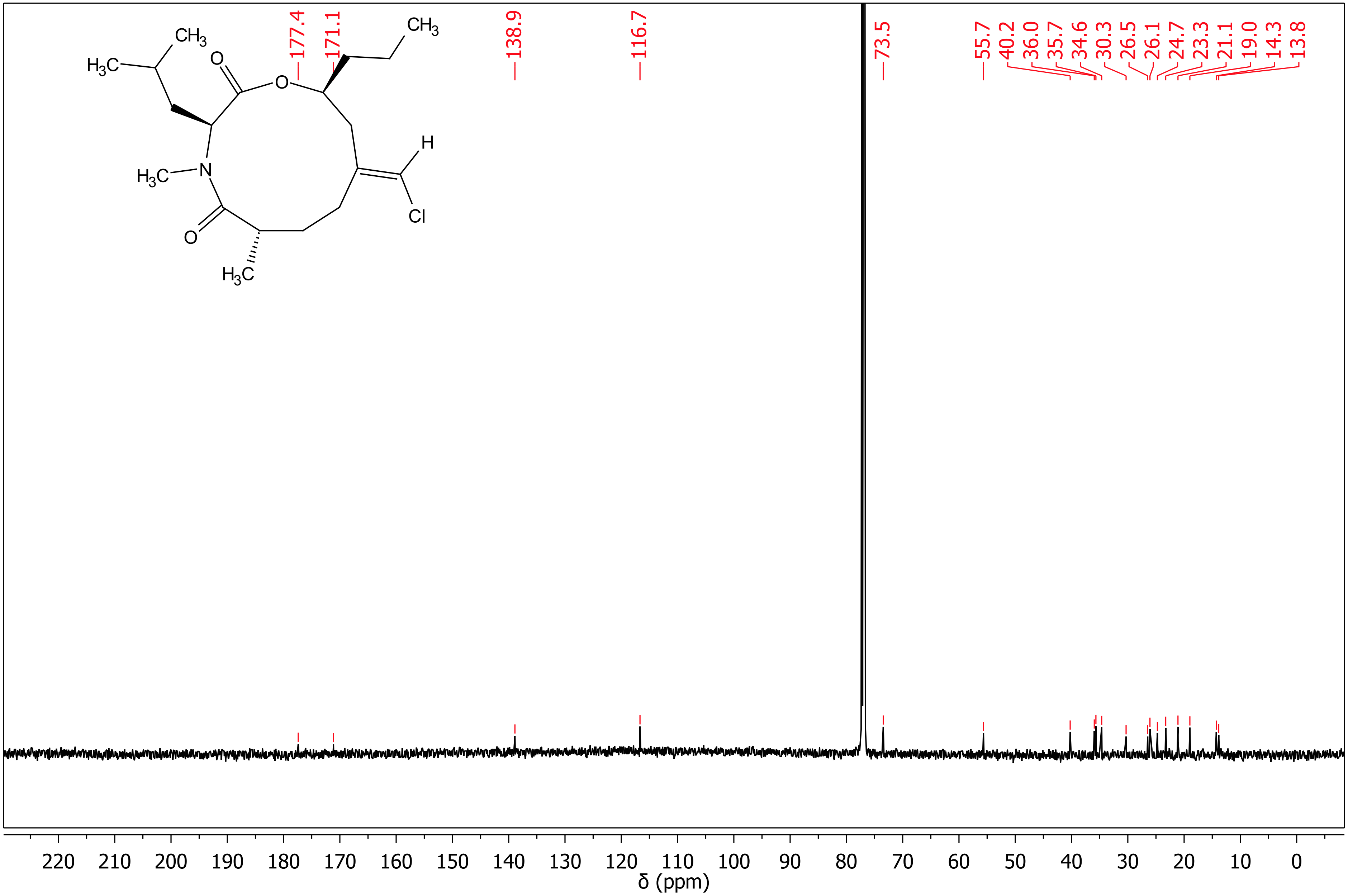

Figure S20. gHSQC Spectrum (500 MHz) of **10** in $CDCl_3$

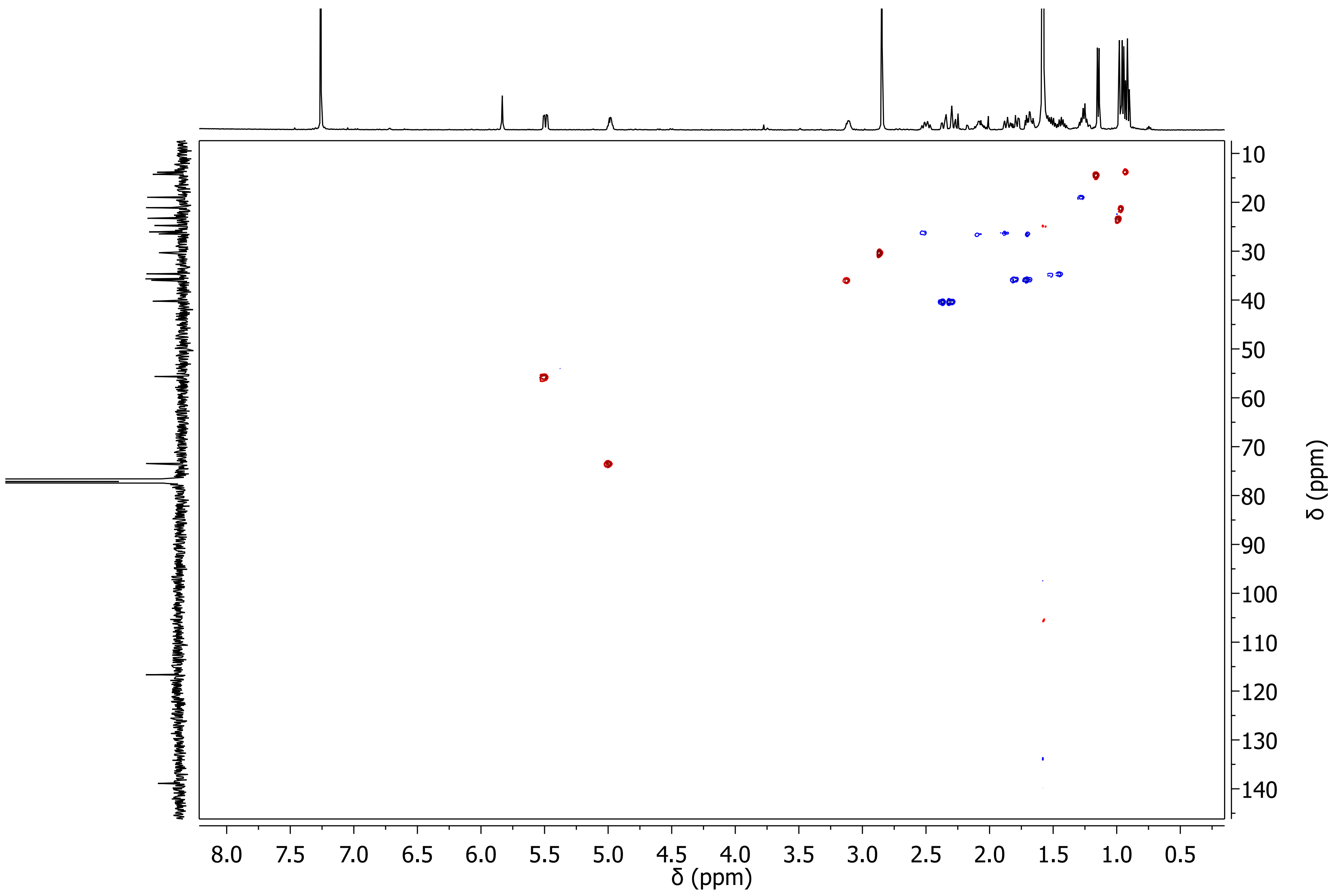

Figure S21. gCOSY NMR Spectrum (500 MHz) of **10** in $CDCl_3$

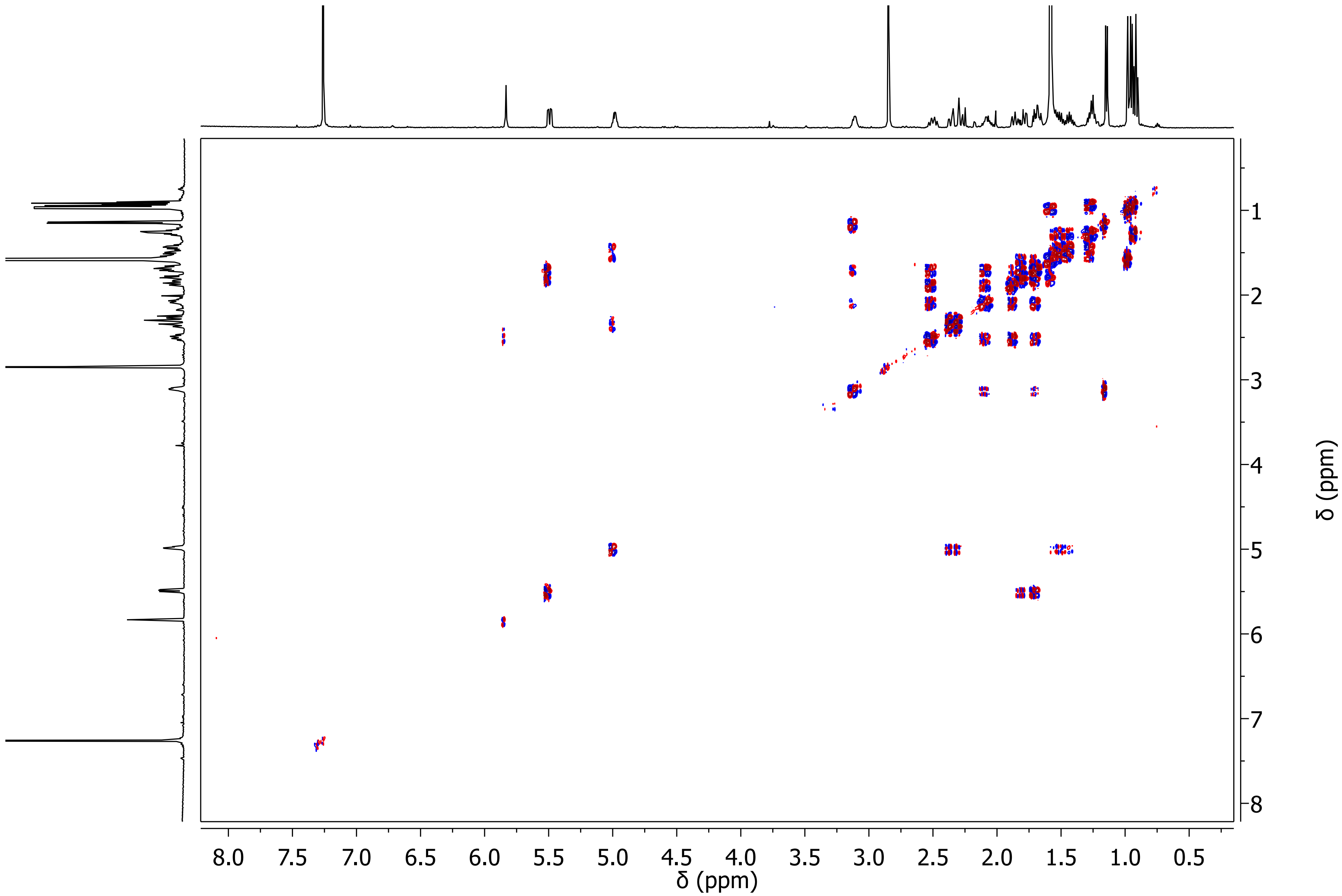

Figure S22. gHMBC NMR Spectrum (500 MHz) of **10** in $CDCl_3$

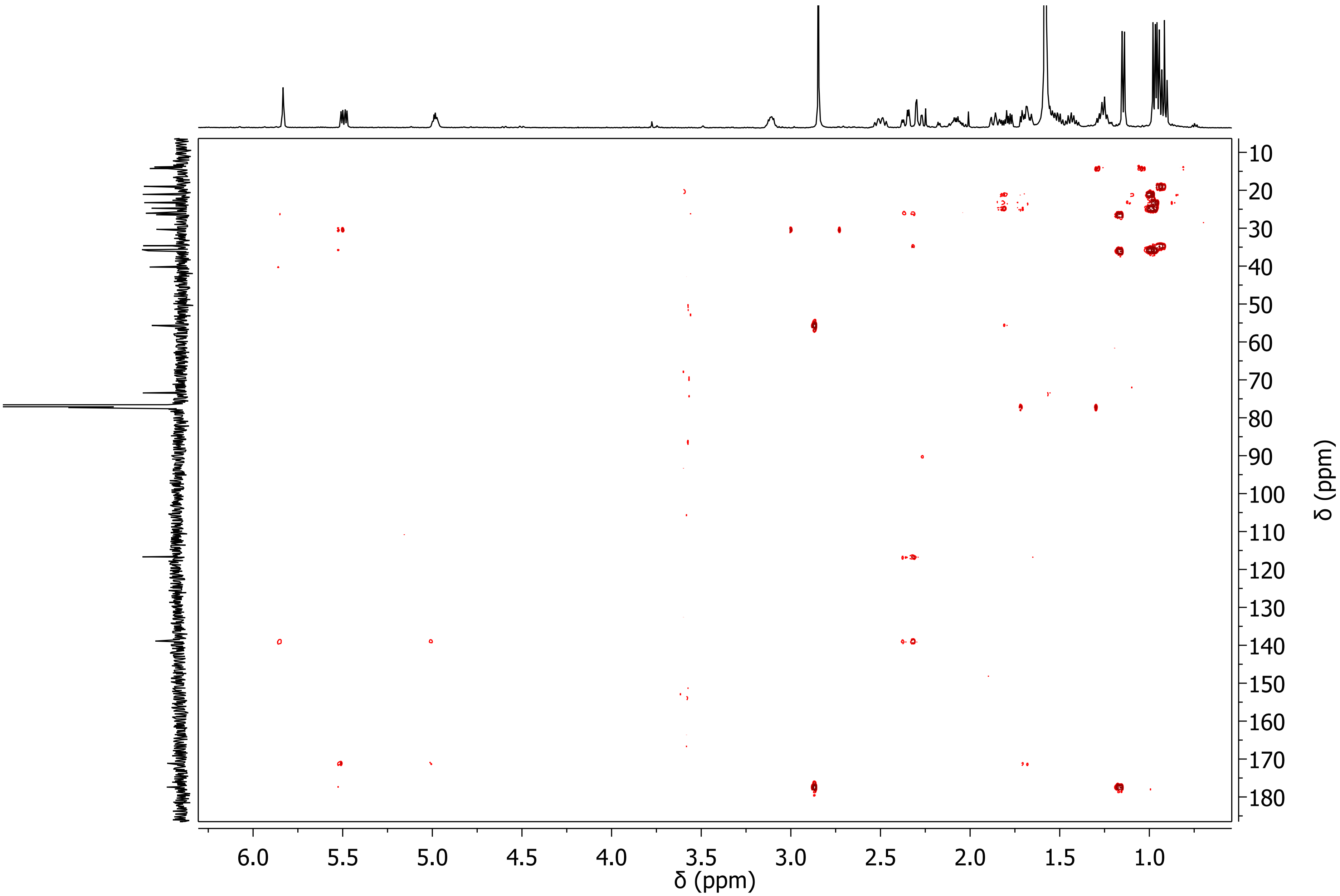

Figure S23. ^{1}H NMR Spectrum (500 MHz) of **11** in $CDCl_3$

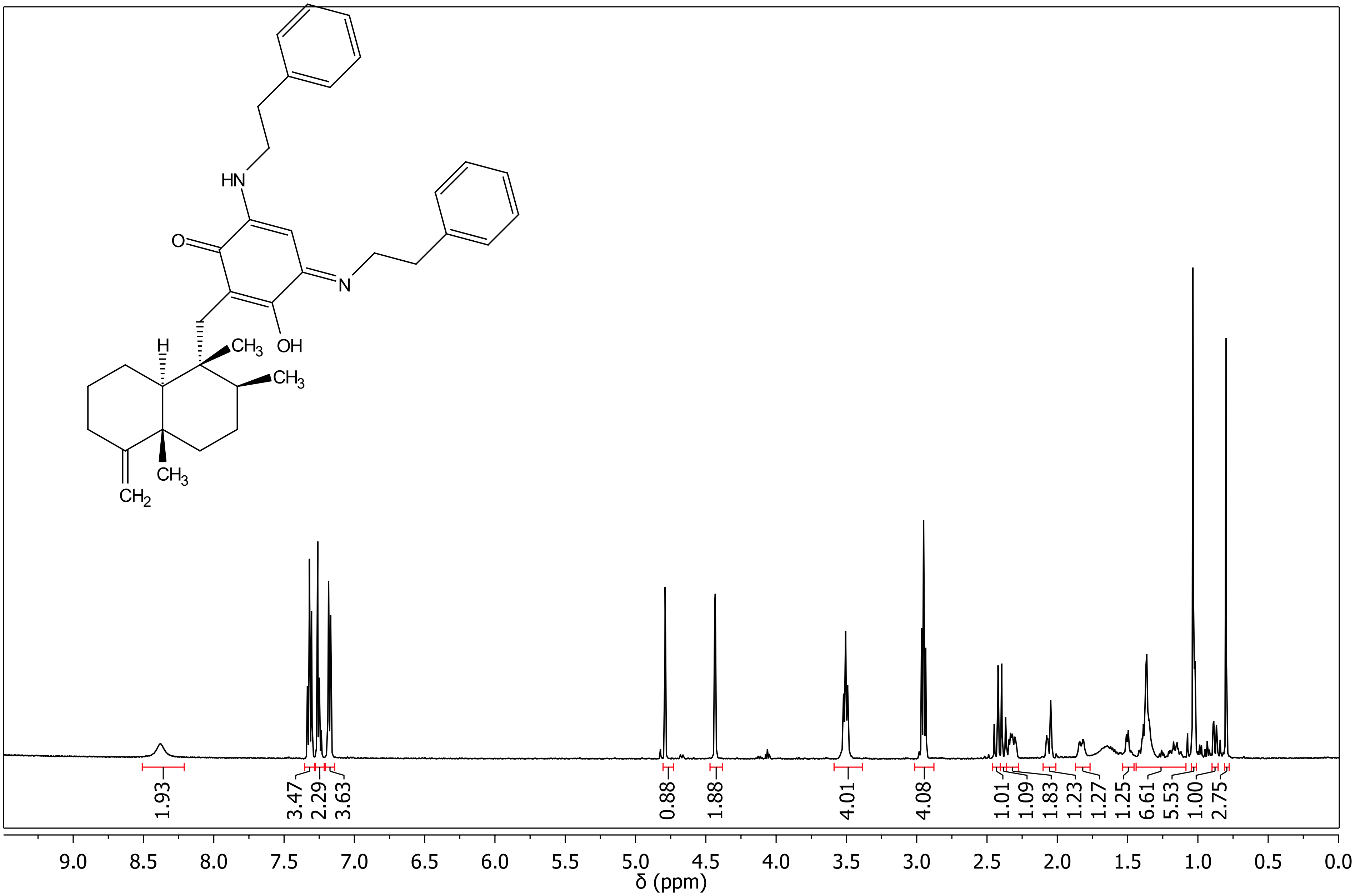

Figure S24. ^{13}C NMR Spectrum (125 MHz) of **11** in $CDCl_3$

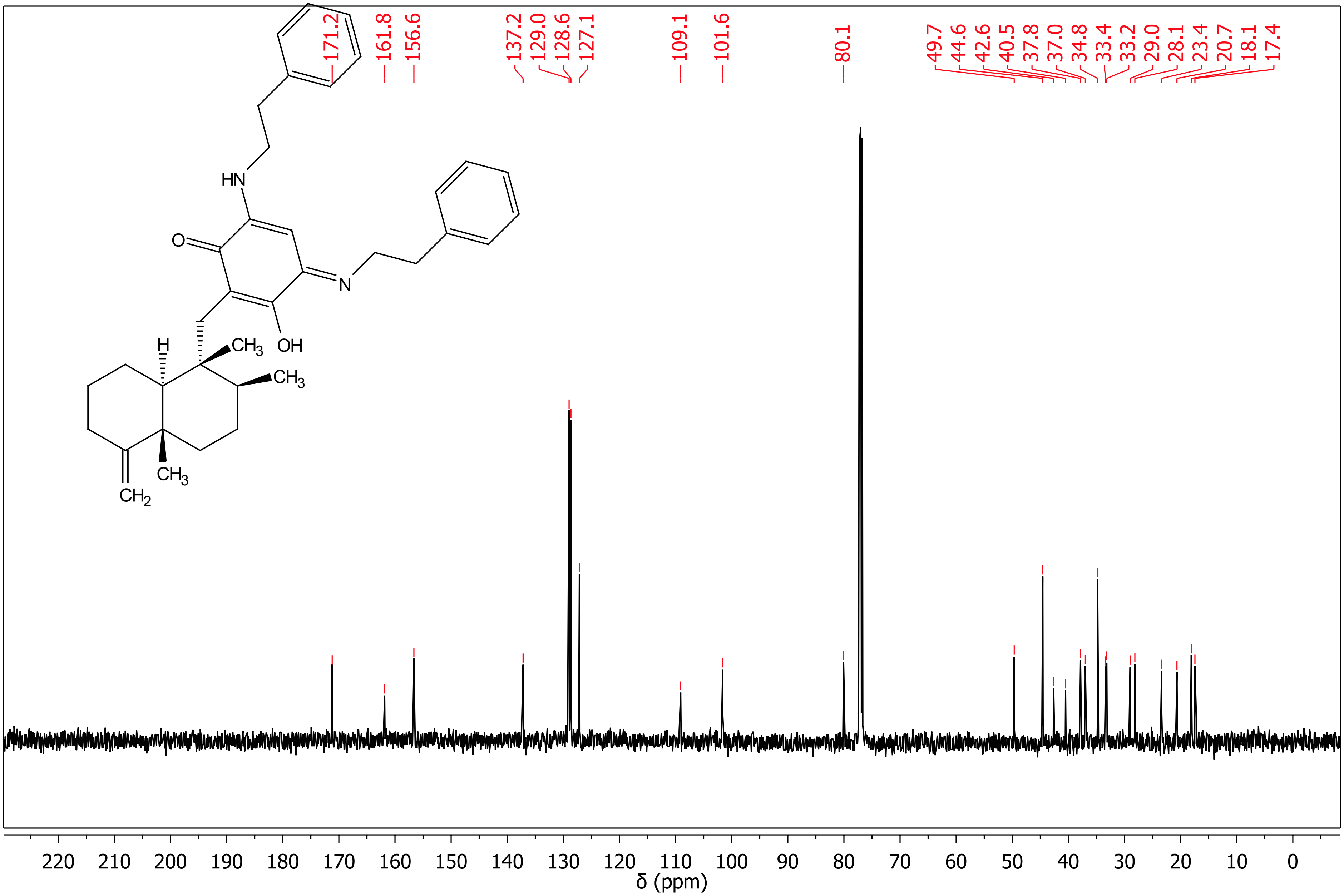

Figure S25. Photograph of the Biological Specimen

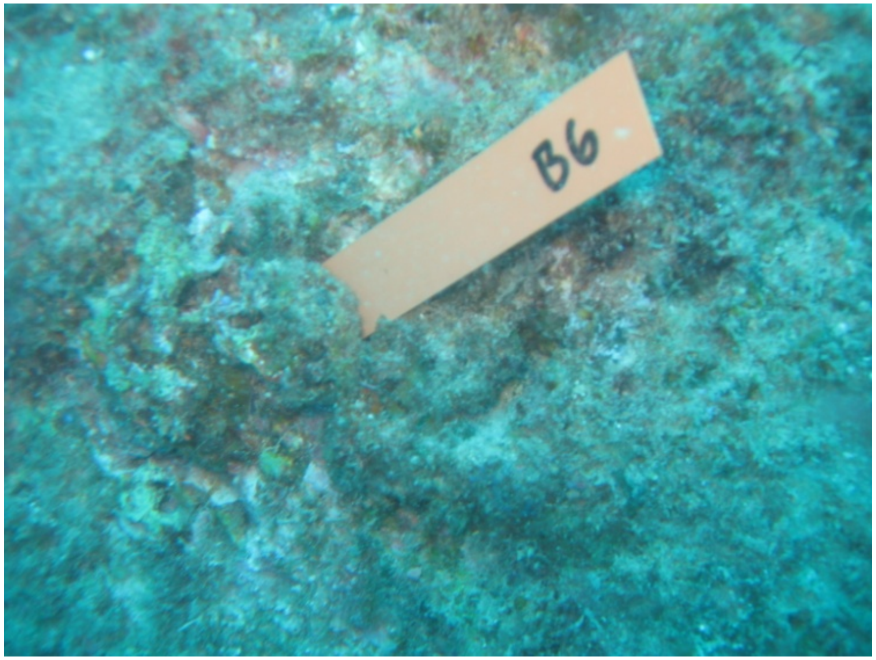

Table S3. Boltzmann Distribution of Conformers of (3*S*,6*S*,11*S*)-**10t**

Conformer	Energy (kcal/mol)	Relative Energy (kcal/mol)	Boltzmann Factor	Equil Mole Fraction	Imaginary Frequency
18	-807441.2574	0.00	1.000	0.454	0
16	-807441.2543	0.00	0.995	0.452	0
4	-807439.9014	1.36	0.101	0.046	0
2	-807439.8506	1.41	0.093	0.042	0
20	-807438.1105	3.15	0.005	0.002	0
19	-807438.0402	3.22	0.004	0.002	0
5	-807437.5269	3.73	0.002	0.001	0
17	-807437.2019	4.06	0.001	0.000	0
15	-807437.1303	4.13	0.001	0.000	0
9	-807436.8002	4.46	0.001	0.000	0
14	-807436.7563	4.50	0.000	0.000	0
12	-807436.3007	4.96	0.000	0.000	0

XYZ coordinates listed in following tables for any conformer with an equilibrium mole fraction greater than or equal to 0.001

Table S4. Cartesian Coordinates For The Optimized Conformer 18 Of Compound 3*S*,6*S*,11*S*-**10t**

#	Atomic	Coordinates (Angstrom)		
		X	Y	Z
1	C	-2.09728	-0.78312	-0.62876
2	C	-1.58618	0.633513	-0.76711
3	C	-1.06482	1.229865	0.556568
4	C	-0.05688	2.38545	0.345564
5	C	1.189215	1.789289	-0.3037
6	N	2.04221	1.039249	0.473594
7	C	2.885086	0.07041	-0.22061
8	C	2.155137	-1.27735	-0.29858
9	O	0.832762	-1.1083	-0.41637
10	C	-0.00738	-2.28492	-0.4442
11	C	-1.2741	-1.91053	-1.21763
12	C	-3.25899	-1.08944	-0.05006
13	H	-3.64744	-2.09778	0.042691
14	Cl	-4.34138	0.107177	0.621223
15	C	-0.26668	-2.74077	0.981607
16	H	0.526018	-3.06372	-0.99809
17	H	-0.98126	-1.63451	-2.2378
18	H	-1.88166	-2.81939	-1.28824
19	H	-0.7841	0.641533	-1.51135
20	H	-2.39343	1.2664	-1.15391
21	H	-1.90596	1.604748	1.148753
22	H	-0.5935	0.435193	1.143747
23	C	-0.63405	3.506924	-0.51353
24	H	0.192014	2.802412	1.32629
25	O	1.380711	1.910677	-1.51079
26	C	1.857183	0.774658	1.892188
27	H	2.976905	0.425344	-1.25152
28	C	4.273296	-0.09268	0.385994
29	O	2.704062	-2.35531	-0.2746
30	H	0.070859	4.338257	-0.59496
31	H	-0.8483	3.156624	-1.52501
32	H	-1.55926	3.87848	-0.06271
33	H	1.440042	-0.22969	2.056639
34	H	1.190052	1.502171	2.345991
35	H	2.81914	0.840578	2.405514
36	H	4.2415	-0.57017	1.368341
37	H	4.881253	-0.72343	-0.26426
38	H	4.748719	0.886726	0.476642
39	H	-0.91271	-3.6237	0.979216
40	H	-0.7661	-1.95277	1.553302
41	H	0.67352	-3.00448	1.472489

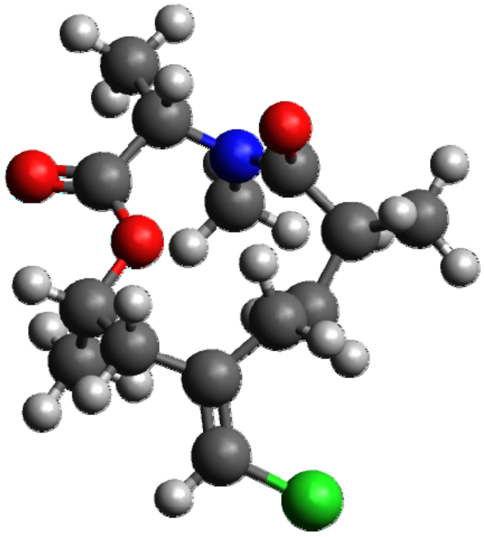

Table S5. Cartesian Coordinates For The Optimized Conformer 16 Of Compound 3*S*,6*S*,11*S*-**10t**

#	Atomic	Coordinates (Angstrom)		
		X	Y	Z
1	C	-2.09728	-0.78312	-0.62876
2	C	-1.58618	0.633513	-0.76711
3	C	-1.06482	1.229865	0.556568
4	C	-0.05688	2.38545	0.345564
5	C	1.189215	1.789289	-0.3037
6	N	2.04221	1.039249	0.473594
7	C	2.885086	0.07041	-0.22061
8	C	2.155137	-1.27735	-0.29858
9	O	0.832762	-1.1083	-0.41637
10	C	-0.00738	-2.28492	-0.4442
11	C	-1.2741	-1.91053	-1.21763
12	C	-3.25899	-1.08944	-0.05006
13	H	-3.64744	-2.09778	0.042691
14	Cl	-4.34138	0.107177	0.621223
15	C	-0.26668	-2.74077	0.981607
16	H	0.526018	-3.06372	-0.99809
17	H	-0.98126	-1.63451	-2.2378
18	H	-1.88166	-2.81939	-1.28824
19	H	-0.7841	0.641533	-1.51135
20	H	-2.39343	1.2664	-1.15391
21	H	-1.90596	1.604748	1.148753
22	H	-0.5935	0.435193	1.143747
23	C	-0.63405	3.506924	-0.51353
24	H	0.192014	2.802412	1.32629
25	O	1.380711	1.910677	-1.51079
26	C	1.857183	0.774658	1.892188
27	H	2.976905	0.425344	-1.25152
28	C	4.273296	-0.09268	0.385994
29	O	2.704062	-2.35531	-0.2746
30	H	0.070859	4.338257	-0.59496
31	H	-0.8483	3.156624	-1.52501
32	H	-1.55926	3.87848	-0.06271
33	H	1.440042	-0.22969	2.056639
34	H	1.190052	1.502171	2.345991
35	H	2.81914	0.840578	2.405514
36	H	4.2415	-0.57017	1.368341
37	H	4.881253	-0.72343	-0.26426
38	H	4.748719	0.886726	0.476642
39	H	-0.91271	-3.6237	0.979216
40	H	-0.7661	-1.95277	1.553302
41	H	0.67352	-3.00448	1.472489

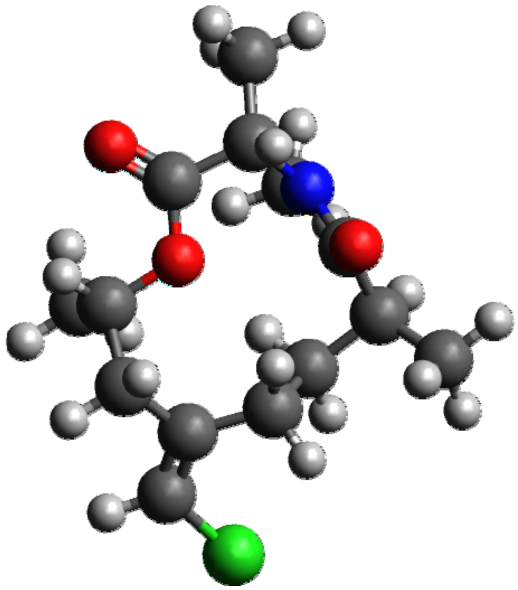

Table S6. Cartesian Coordinates For The Optimized Conformer 4 Of Compound 3*S*,6*S*,11*S*-**10t**

#	Atomic	Coordinates (Angstrom)		
		X	Y	Z
1	C	2.126474	-0.79331	-0.24078
2	C	1.545329	0.511533	0.235634
3	C	0.931444	1.339713	-0.90834
4	C	-0.01064	2.466335	-0.4164
5	C	-1.15977	1.787985	0.321451
6	N	-2.16402	1.179884	-0.39084
7	C	-2.90197	0.175848	0.377092
8	C	-1.98059	-0.98693	0.762811
9	O	-0.97013	-1.12943	-0.10536
10	C	-0.0156	-2.19882	0.107781
11	C	1.190467	-1.86182	-0.77212
12	C	3.431156	-1.07341	-0.24094
13	H	3.842055	-2.02146	-0.57008
14	Cl	4.678673	0.036939	0.273457
15	C	-0.64386	-3.52453	-0.29342
16	H	0.250787	-2.20126	1.171352
17	H	1.758216	-2.78349	-0.93542
18	H	0.795868	-1.56196	-1.75303
19	H	0.776099	0.293323	0.985226
20	H	2.317735	1.094812	0.741222
21	H	1.737368	1.79165	-1.49865
22	H	0.377346	0.677186	-1.58136
23	C	0.703581	3.462607	0.493454
24	H	-0.38066	3.005241	-1.29378
25	O	-1.1318	1.683968	1.547346
26	C	-2.19878	1.092528	-1.84437
27	H	-3.20816	0.627423	1.322416
28	C	-4.13689	-0.37124	-0.33709
29	O	-2.17356	-1.71089	1.711575
30	H	0.038041	4.284606	0.769706
31	H	1.036612	2.983119	1.415629
32	H	1.572125	3.879312	-0.02522
33	H	-1.77435	0.144796	-2.19745
34	H	-1.64087	1.91516	-2.28479
35	H	-3.2298	1.172669	-2.19344
36	H	-3.88242	-0.91685	-1.24962
37	H	-4.65218	-1.05607	0.339571
38	H	-4.8207	0.443204	-0.58844
39	H	0.059556	-4.34019	-0.10634
40	H	-0.88898	-3.51432	-1.36044
41	H	-1.5512	-3.71214	0.282944

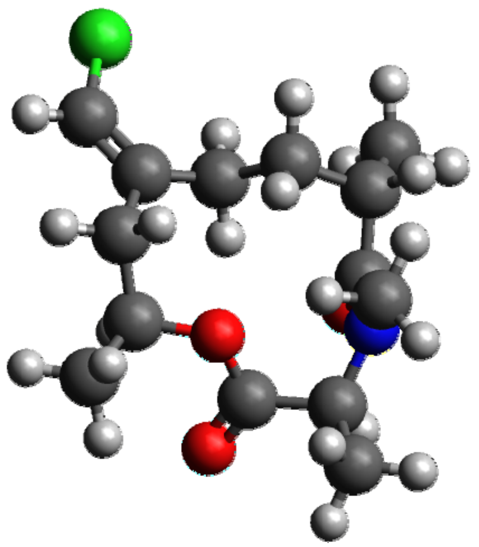

Table S7. Cartesian Coordinates For The Optimized Conformer 2 Of Compound 3*S*,6*S*,11*S*-**10t**

#	Atomic	Coordinates (Angstrom)		
		X	Y	Z
1	C	-2.07315	-0.48912	-0.29961
2	C	-1.43687	0.603364	0.521022
3	C	-1.0973	1.851339	-0.32967
4	C	0.255767	2.479681	0.044946
5	C	1.327997	1.461361	-0.33877
6	N	2.135484	0.863636	0.590522
7	C	2.934359	-0.23857	0.042244
8	C	2.024693	-1.32548	-0.53676
9	O	0.876512	-1.41977	0.147254
10	C	-0.18979	-2.24288	-0.38289
11	C	-1.16672	-1.3442	-1.1603
12	C	-3.38545	-0.72155	-0.3407
13	H	-3.84361	-1.49498	-0.94733
14	Cl	-4.56705	0.195154	0.565782
15	C	-0.81662	-2.96971	0.790902
16	H	0.258144	-2.9532	-1.08253
17	H	-0.56961	-0.7012	-1.82161
18	H	-1.77431	-1.99326	-1.80025
19	H	-2.0907	0.884613	1.350408
20	H	-0.519	0.199663	0.959465
21	H	-1.05356	1.590613	-1.39302
22	H	-1.88826	2.600359	-0.21741
23	C	0.496358	3.779226	-0.72827
24	H	0.25607	2.69757	1.114897
25	O	1.436492	1.146948	-1.52631
26	C	1.935053	0.93327	2.032694
27	H	3.492625	0.143848	-0.81548
28	C	3.911776	-0.86167	1.036515
29	O	2.338195	-2.03714	-1.4617
30	H	1.462829	4.223229	-0.47263
31	H	0.481733	3.582082	-1.80324
32	H	-0.28727	4.50445	-0.49254
33	H	1.327921	0.092621	2.390913
34	H	1.446386	1.864956	2.303255
35	H	2.899971	0.91423	2.540371
36	H	3.402185	-1.32322	1.886165
37	H	4.481308	-1.63498	0.516244
38	H	4.615705	-0.11199	1.405629
39	H	-1.6687	-3.56269	0.446124
40	H	-1.17905	-2.25897	1.539567
41	H	-0.08969	-3.63742	1.25896

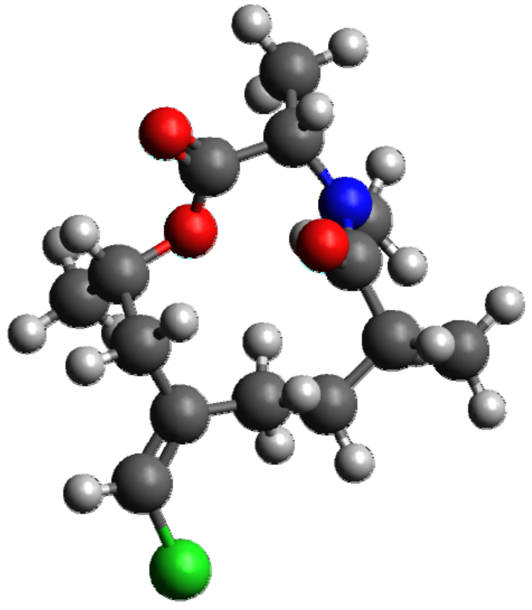

Table S8. Cartesian Coordinates For The Optimized Conformer 20 Of Compound 3*S*,6*S*,11*S*-**10t**

#	Atomic	Coordinates (Angstrom)		
		X	Y	Z
1	C	2.116047	0.175134	-0.48364
2	C	1.768573	-0.69901	-1.66545
3	C	0.254105	-0.89501	-1.89927
4	C	-0.4887	-1.40793	-0.64887
5	C	-1.93529	-0.93826	-0.69878
6	N	-2.42002	-0.12674	0.290677
7	C	-1.64368	0.477963	1.370147
8	C	-0.44958	1.334811	0.931989
9	O	-0.40782	1.53534	-0.38139
10	C	0.692598	2.263716	-0.98577
11	C	2.06755	1.682576	-0.6185
12	C	2.483325	-0.30022	0.708677
13	H	2.715991	0.331734	1.558393
14	Cl	2.615534	-1.99858	1.096044
15	C	0.590661	3.749522	-0.68316
16	H	0.50473	2.096798	-2.05072
17	H	2.753949	1.993615	-1.41749
18	H	2.4159	2.141948	0.309903
19	H	2.202255	-0.25586	-2.57031
20	H	2.241126	-1.67733	-1.53337
21	H	-0.19345	0.041402	-2.23857
22	H	0.118058	-1.61252	-2.71575
23	C	-0.46392	-2.93808	-0.56301
24	H	0.012249	-1.00681	0.231998
25	O	-2.65218	-1.30685	-1.62933
26	C	-3.81178	0.304816	0.20742
27	H	-2.31264	1.222453	1.820755
28	C	-1.2585	-0.49515	2.489832
29	O	0.32867	1.804188	1.732606
30	H	-0.88223	-3.29149	0.385147
31	H	-1.05017	-3.36293	-1.38206
32	H	0.564589	-3.30264	-0.63433
33	H	-4.34047	-0.33937	-0.49089
34	H	-3.87607	1.341877	-0.14261
35	H	-4.27599	0.232328	1.19513
36	H	-0.56961	-1.26883	2.142133
37	H	-0.78129	0.050641	3.305506
38	H	-2.16342	-0.97979	2.863931
39	H	1.35831	4.285765	-1.24856
40	H	0.743937	3.937782	0.381569
41	H	-0.38763	4.133395	-0.98217

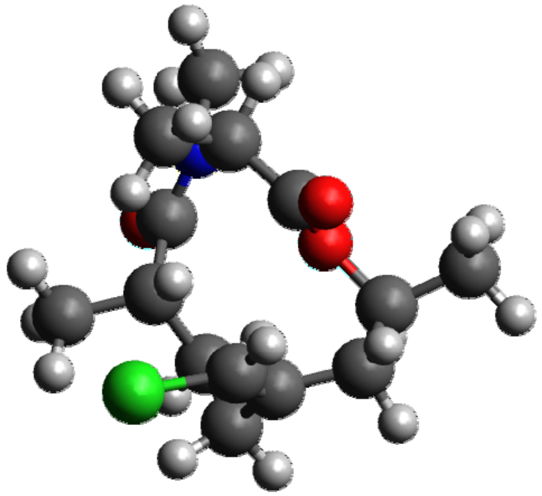

Table S9. Cartesian Coordinates For The Optimized Conformer 19 Of Compound 3*S*,6*S*,11*S*-**10t**

#	Atomic	Coordinates (Angstrom)		
		X	Y	Z
1	C	2.116047	0.175134	-0.48364
2	C	1.768573	-0.69901	-1.66545
3	C	0.254105	-0.89501	-1.89927
4	C	-0.4887	-1.40793	-0.64887
5	C	-1.93529	-0.93826	-0.69878
6	N	-2.42002	-0.12674	0.290677
7	C	-1.64368	0.477963	1.370147
8	C	-0.44958	1.334811	0.931989
9	O	-0.40782	1.53534	-0.38139
10	C	0.692598	2.263716	-0.98577
11	C	2.06755	1.682576	-0.6185
12	C	2.483325	-0.30022	0.708677
13	H	2.715991	0.331734	1.558393
14	Cl	2.615534	-1.99858	1.096044
15	C	0.590661	3.749522	-0.68316
16	H	0.50473	2.096798	-2.05072
17	H	2.753949	1.993615	-1.41749
18	H	2.4159	2.141948	0.309903
19	H	2.202255	-0.25586	-2.57031
20	H	2.241126	-1.67733	-1.53337
21	H	-0.19345	0.041402	-2.23857
22	H	0.118058	-1.61252	-2.71575
23	C	-0.46392	-2.93808	-0.56301
24	H	0.012249	-1.00681	0.231998
25	O	-2.65218	-1.30685	-1.62933
26	C	-3.81178	0.304816	0.20742
27	H	-2.31264	1.222453	1.820755
28	C	-1.2585	-0.49515	2.489832
29	O	0.32867	1.804188	1.732606
30	H	-0.88223	-3.29149	0.385147
31	H	-1.05017	-3.36293	-1.38206
32	H	0.564589	-3.30264	-0.63433
33	H	-4.34047	-0.33937	-0.49089
34	H	-3.87607	1.341877	-0.14261
35	H	-4.27599	0.232328	1.19513
36	H	-0.56961	-1.26883	2.142133
37	H	-0.78129	0.050641	3.305506
38	H	-2.16342	-0.97979	2.863931
39	H	1.35831	4.285765	-1.24856
40	H	0.743937	3.937782	0.381569
41	H	-0.38763	4.133395	-0.98217

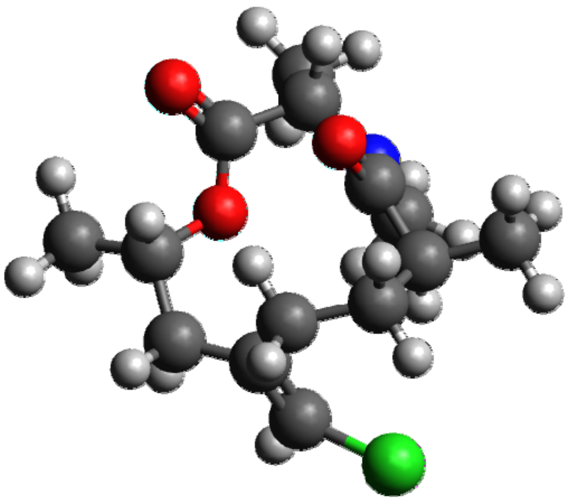

Table S10. Cartesian Coordinates For The Optimized Conformer 5 Of Compound 3*S*,6*S*,11*S*-**10t**

#	Atomic	Coordinates (Angstrom)		
		X	Y	Z
1	C	-1.58397	-1.1909	0.614494
2	C	-2.1604	-0.06857	1.440886
3	C	-1.1991	1.080812	1.807102
4	C	-0.9282	2.155694	0.722043
5	C	0.063973	1.646185	-0.31655
6	N	1.409485	1.675233	-0.02305
7	C	2.232889	0.898625	-0.94641
8	C	1.906032	-0.59355	-0.84569
9	O	1.261004	-0.88865	0.286001
10	C	0.754564	-2.22752	0.46202
11	C	-0.52314	-2.06201	1.276452
12	C	-2.00048	-1.52397	-0.60603
13	H	-1.60134	-2.36123	-1.16874
14	Cl	-3.25231	-0.69298	-1.49073
15	C	1.804078	-3.07583	1.157915
16	H	0.539509	-2.63441	-0.53072
17	H	-0.93301	-3.05822	1.481592
18	H	-0.25267	-1.63015	2.247724
19	H	-3.05621	0.332688	0.961584
20	H	-2.48938	-0.51604	2.388955
21	H	-1.64122	1.606372	2.661199
22	H	-0.24878	0.66229	2.160759
23	C	-2.20352	2.646039	0.040914
24	H	-0.48167	3.011547	1.238373
25	O	-0.3207	1.200269	-1.39388
26	C	1.947681	1.918649	1.308991
27	H	1.951004	1.179868	-1.96368
28	C	3.736135	1.112689	-0.77556
29	O	2.224417	-1.40561	-1.68461
30	H	-1.99334	3.521909	-0.57877
31	H	-2.62365	1.874932	-0.60654
32	H	-2.94596	2.927795	0.794673
33	H	2.12798	0.97615	1.841251
34	H	1.260466	2.523192	1.895124
35	H	2.886524	2.470096	1.232979
36	H	4.10143	0.756044	0.191104
37	H	4.254428	0.55974	-1.56125
38	H	3.980546	2.172806	-0.87852
39	H	1.427282	-4.0912	1.309068
40	H	2.053171	-2.64653	2.133281
41	H	2.710737	-3.12981	0.55069

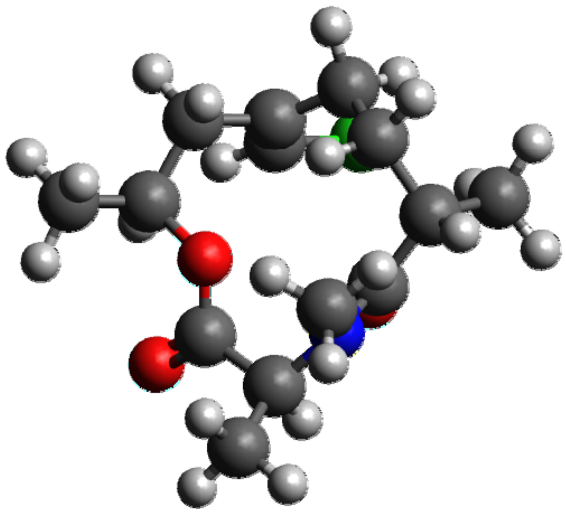

Table S11. Boltzmann Distribution of Conformers of (3R,6*S*,11*S*)-**10t**

Conformer	Energy (kcal/mol)	Relative Energy (kcal/mol)	Boltzmann Factor	Equil Mole Fraction	Imaginary Frequency
1	-807440.62	0.00	1.000	0.630	0
4	-807439.76	0.86	0.235	0.148	0
8	-807439.69	0.93	0.208	0.131	0
14	-807438.99	1.63	0.064	0.040	0
5	-807438.43	2.19	0.025	0.016	0
2	-807438.38	2.24	0.023	0.014	0
3	-807438.14	2.48	0.015	0.010	0
7	-807437.32	3.30	0.004	0.002	0
15	-807437.31	3.30	0.004	0.002	0
6	-807437.21	3.41	0.003	0.002	0
13	-807437.08	3.54	0.003	0.002	0
9	-807436.84	3.78	0.002	0.001	0
10	-807436.51	4.11	0.001	0.001	0

XYZ coordinates listed in following tables for any conformer with an equilibrium mole fraction greater than or equal to 0.001

Table S12. Cartesian Coordinates For The Optimized Conformer 1 Of Compound 3*R*,6*S*,11*S*-**10t**

#	Atomic	Coordinates (Angstrom)		
		X	Y	Z
1	C	2.146296	-0.02849	-0.73227
2	C	1.659595	-1.09649	-1.67668
3	C	0.135383	-1.03744	-1.85455
4	C	-0.63334	-1.40609	-0.5696
5	C	-2.03617	-0.83365	-0.72085
6	N	-2.42522	0.209463	0.07311
7	C	-1.7584	0.616524	1.30512
8	C	-0.35522	1.206889	1.147885
9	O	-0.09387	1.652471	-0.08317
10	C	1.181418	2.306442	-0.31614
11	C	2.030218	1.408498	-1.21773
12	C	2.606278	-0.25788	0.496657
13	H	2.932713	0.519684	1.178536
14	Cl	2.696318	-1.84432	1.224446
15	C	0.885297	3.652512	-0.95055
16	H	1.668659	2.436144	0.652873
17	H	1.595965	1.411271	-2.22429
18	H	3.022571	1.866841	-1.30373
19	H	2.132406	-0.93478	-2.65357
20	H	1.9667	-2.08672	-1.3304
21	H	-0.15026	-0.02712	-2.17078
22	H	-0.17504	-1.71634	-2.65574
23	C	-0.70401	-2.92128	-0.36379
24	H	-0.10858	-0.9649	0.281739
25	O	-2.76645	-1.27294	-1.60877
26	C	-3.74736	0.78904	-0.13981
27	H	-2.33573	1.475628	1.672129
28	C	-1.79232	-0.45222	2.400873
29	O	0.393072	1.30821	2.094197
30	H	-1.20782	-3.17505	0.574201
31	H	-1.25867	-3.38059	-1.18612
32	H	0.302392	-3.34773	-0.32911
33	H	-4.44212	0.477682	0.649563
34	H	-4.12896	0.450707	-1.10007
35	H	-3.67051	1.879808	-0.137
36	H	-1.1684	-1.31123	2.140797
37	H	-1.42747	-0.03921	3.342028
38	H	-2.82175	-0.79403	2.533498
39	H	0.292381	4.274526	-0.27597
40	H	0.331441	3.518954	-1.88444
41	H	1.821149	4.173404	-1.17093

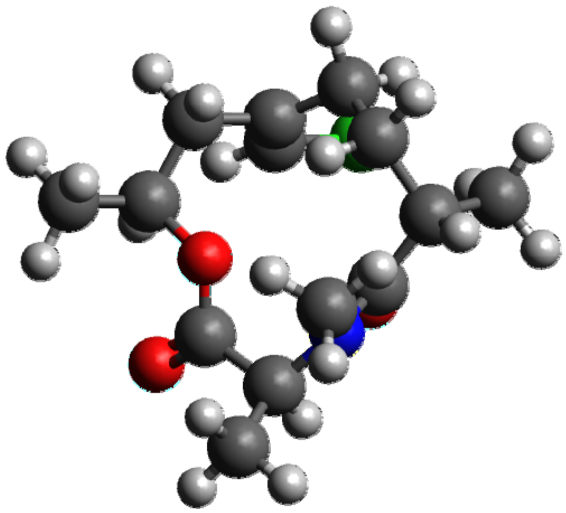

Table S13. Cartesian Coordinates For The Optimized Conformer 4 Of Compound 3*R*,6*S*,11*S*-**10t**

#	Atomic	Coordinates (Angstrom)		
		X	Y	Z
1	C	-2.22129	0.263737	-0.28718
2	C	-1.43508	-0.83072	0.388849
3	C	-0.65974	-1.74438	-0.59056
4	C	0.826408	-1.41138	-0.80239
5	C	1.534057	-1.4102	0.552828
6	N	2.391094	-0.39771	0.878678
7	C	2.736707	0.740863	0.032459
8	C	1.537405	1.562116	-0.43677
9	O	0.538492	1.565568	0.445706
10	C	-0.66656	2.278236	0.06155
11	C	-1.48393	1.423838	-0.91749
12	C	-3.55217	0.261813	-0.38933
13	H	-4.11562	1.045365	-0.88407
14	Cl	-4.59264	-0.99641	0.233984
15	C	-1.39606	2.618732	1.345071
16	H	-0.35304	3.188857	-0.45674
17	H	-2.20165	2.089512	-1.40932
18	H	-0.80986	1.06144	-1.70422
19	H	-0.73572	-0.38511	1.105662
20	H	-2.12291	-1.44681	0.970705
21	H	-0.69871	-2.76692	-0.20415
22	H	-1.15627	-1.7528	-1.56844
23	C	1.472804	-2.49603	-1.6786
24	H	0.923207	-0.45113	-1.31705
25	O	1.327532	-2.34712	1.323435
26	C	3.081209	-0.44661	2.163658
27	H	3.275259	1.433818	0.692992
28	C	3.661363	0.394354	-1.13301
29	O	1.527784	2.171885	-1.48286
30	H	2.541024	-2.31968	-1.83205
31	H	1.355489	-3.47116	-1.19771
32	H	0.985278	-2.5278	-2.65706
33	H	4.163829	-0.39148	2.008378
34	H	2.834028	-1.38096	2.661132
35	H	2.767395	0.395229	2.789855
36	H	3.136167	-0.17921	-1.89973
37	H	4.040673	1.30628	-1.59674
38	H	4.502548	-0.19617	-0.76183
39	H	-0.78192	3.2692	1.971719
40	H	-1.64453	1.715397	1.908579
41	H	-2.32859	3.138137	1.106846

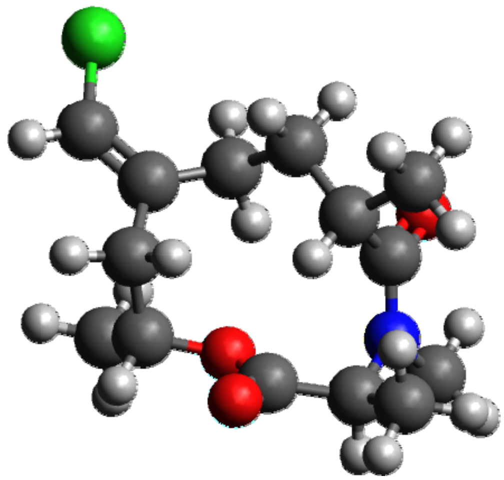

Table S14. Cartesian Coordinates For The Optimized Conformer 8 Of Compound 3*R*,6*S*,11*S*-**10t**

#	Atomic	Coordinates (Angstrom)		
		X	Y	Z
1	C	1.988937	-0.66096	-0.35966
2	C	1.428655	0.592146	0.263478
3	C	0.788576	1.516887	-0.79225
4	C	-0.24231	2.502159	-0.19448
5	C	-1.32944	1.673027	0.49331
6	N	-2.15698	0.90571	-0.29369
7	C	-2.84662	-0.22737	0.338406
8	C	-1.98622	-1.44991	0.008381
9	O	-0.86353	-1.45	0.737425
10	C	0.190841	-2.36825	0.358727
11	C	1.025414	-1.75379	-0.77673
12	C	3.287056	-0.85683	-0.59688
13	H	3.688644	-1.75587	-1.05145
14	Cl	4.535481	0.307549	-0.22339
15	C	0.984223	-2.66794	1.615055
16	H	-0.28581	-3.27609	-0.02122
17	H	1.583545	-2.56714	-1.25304
18	H	0.327431	-1.37366	-1.53541
19	H	0.674647	0.30697	1.004461
20	H	2.217013	1.128078	0.797436
21	H	1.57494	2.088397	-1.29874
22	H	0.306176	0.906751	-1.56341
23	C	0.397894	3.470979	0.795741
24	H	-0.6742	3.08707	-1.01369
25	O	-1.38868	1.616336	1.717414
26	C	-2.25409	0.985886	-1.74564
27	H	-2.80011	-0.05311	1.414111
28	C	-4.28485	-0.40324	-0.12044
29	O	-2.23952	-2.26046	-0.85518
30	H	-0.33898	4.179523	1.181515
31	H	0.81913	2.937976	1.650588
32	H	1.194794	4.031785	0.298637
33	H	-1.88046	0.070563	-2.22168
34	H	-1.68839	1.832378	-2.12433
35	H	-3.29778	1.124909	-2.03922
36	H	-4.34426	-0.71043	-1.16646
37	H	-4.76349	-1.18091	0.479398
38	H	-4.83411	0.531278	0.018374
39	H	0.349196	-3.15206	2.360102
40	H	1.398078	-1.75222	2.046066
41	H	1.815127	-3.3364	1.372045

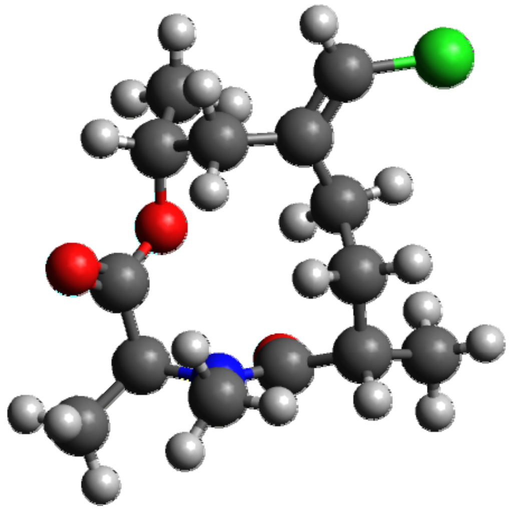

Table S15. Cartesian Coordinates For The Optimized Conformer 14 Of Compound 3*R*,6*S*,11*S*-**10t**

#	Atomic	Coordinates (Angstrom)		
		X	Y	Z
1	C	2.072258	0.897779	-0.38656
2	C	1.645861	-0.49842	-0.78356
3	C	1.16595	-1.36855	0.395552
4	C	0.207755	-2.50179	-0.04269
5	C	-1.07418	-1.835	-0.53274
6	N	-1.89645	-1.25072	0.403771
7	C	-2.77324	-0.16919	-0.05138
8	C	-2.03764	1.144096	0.241248
9	O	-0.89579	1.216741	-0.45597
10	C	-0.05311	2.377314	-0.25648
11	C	1.28848	2.076266	-0.92479
12	C	3.147428	1.159708	0.357945
13	H	3.473393	2.159281	0.623665
14	Cl	4.217404	-0.07381	0.978115
15	C	-0.69909	3.608249	-0.87685
16	H	0.077802	2.508628	0.824321
17	H	1.103776	1.916509	-1.99557
18	H	1.889891	2.987205	-0.83532
19	H	0.843961	-0.41504	-1.52352
20	H	2.489076	-0.99619	-1.2788
21	H	2.024899	-1.81707	0.902956
22	H	0.669711	-0.72542	1.131111
23	C	0.812605	-3.38989	-1.12575
24	H	-0.00072	-3.12763	0.830319
25	O	-1.31074	-1.7375	-1.7332
26	C	-1.71871	-1.33581	1.847248
27	H	-2.8495	-0.27306	-1.13562
28	C	-4.15491	-0.19027	0.583152
29	O	-2.40623	1.982934	1.031776
30	H	0.141249	-4.21673	-1.37089
31	H	0.989664	-2.82686	-2.04412
32	H	1.762184	-3.80542	-0.77505
33	H	-2.67769	-1.55879	2.320901
34	H	-1.33922	-0.39244	2.262516
35	H	-1.02722	-2.13088	2.110468
36	H	-4.12066	0.045786	1.648634
37	H	-4.78807	0.559068	0.102982
38	H	-4.60703	-1.17488	0.441422
39	H	-1.6577	3.828705	-0.4067
40	H	-0.85049	3.447685	-1.94883
41	H	-0.04282	4.472988	-0.74602

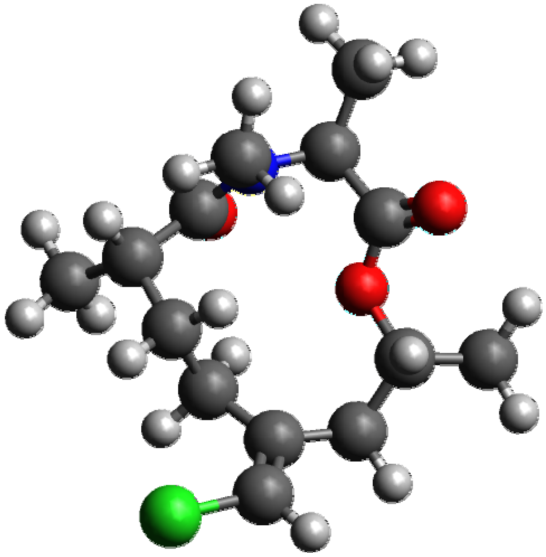

Table S16. Cartesian Coordinates For The Optimized Conformer 5 Of Compound 3*R*,6*S*,11*S*-**10t**

#	Atomic	Coordinates (Angstrom)		
		X	Y	Z
1	C	-2.16399	-0.59255	-0.16789
2	C	-1.36657	0.547481	0.410392
3	C	-1.06727	1.647435	-0.6264
4	C	0.153509	2.527678	-0.27534
5	C	1.385808	1.650942	-0.50544
6	N	1.959918	0.966409	0.537888
7	C	2.792318	-0.19353	0.200558
8	C	1.885309	-1.43143	0.254264
9	O	0.838075	-1.27818	-0.56366
10	C	-0.22708	-2.25895	-0.52204
11	C	-1.46376	-1.56302	-1.09885
12	C	-3.46197	-0.78863	0.069068
13	H	-4.03398	-1.60934	-0.34945
14	Cl	-4.44226	0.250447	1.075928
15	C	0.175866	-3.4735	-1.34258
16	H	-0.38339	-2.53794	0.526956
17	H	-1.14352	-1.04358	-2.01228
18	H	-2.1731	-2.33739	-1.40849
19	H	-1.89293	0.983272	1.26406
20	H	-0.42885	0.134809	0.787779
21	H	-0.87871	1.206701	-1.61255
22	H	-1.9502	2.289031	-0.72839
23	C	0.212758	3.757102	-1.18163
24	H	0.064938	2.863929	0.760198
25	O	1.788924	1.496444	-1.65562
26	C	1.62422	1.09843	1.94964
27	H	3.097993	-0.06054	-0.83967
28	C	4.011357	-0.35253	1.094967
29	O	2.060575	-2.39536	0.964672
30	H	1.099388	4.361962	-0.97215
31	H	0.250461	3.452389	-2.22989
32	H	-0.67358	4.377507	-1.0235
33	H	2.506108	1.400812	2.521264
34	H	1.263904	0.1427	2.352206
35	H	0.847751	1.84124	2.104007
36	H	3.737304	-0.59625	2.123719
37	H	4.6326	-1.16822	0.719556
38	H	4.598183	0.569186	1.084015
39	H	1.103934	-3.90181	-0.95921
40	H	0.317029	-3.18793	-2.38957
41	H	-0.60598	-4.23583	-1.29002

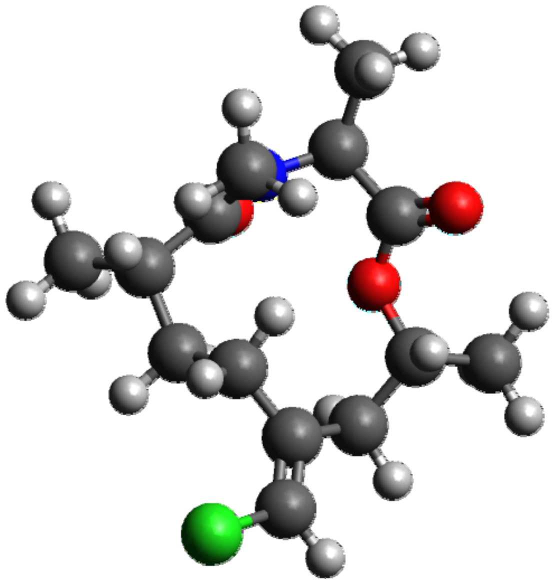

Table S17. Cartesian Coordinates For The Optimized Conformer 2 Of Compound 3*R*,6*S*,11*S*-**10t**

#	Atomic	Coordinates (Angstrom)		
		X	Y	Z
1	C	1.992641	0.053466	-0.77952
2	C	1.2061	-0.76903	-1.77925
3	C	0.406053	-1.98139	-1.29873
4	C	-0.65645	-1.64842	-0.24715
5	C	-1.74354	-0.76943	-0.86721
6	N	-2.3273	0.211817	-0.11604
7	C	-1.85374	0.682943	1.182119
8	C	-0.41236	1.193423	1.172261
9	O	-0.08596	1.747579	-0.00029
10	C	1.215661	2.374948	-0.12027
11	C	2.064879	1.544714	-1.08424
12	C	2.632474	-0.34674	0.319061
13	H	3.177691	0.345222	0.953048
14	Cl	2.716606	-1.973	0.949176
15	C	0.992533	3.796004	-0.60113
16	H	1.675793	2.367295	0.870808
17	H	1.72632	1.722508	-2.11098
18	H	3.094273	1.912849	-1.01641
19	H	0.504256	-0.08717	-2.27466
20	H	1.905999	-1.08895	-2.56455
21	H	-0.10842	-2.4002	-2.16933
22	H	1.058828	-2.76062	-0.90269
23	C	-1.32385	-2.94354	0.24392
24	H	-0.17193	-1.15152	0.600889
25	O	-2.12866	-1.00449	-2.01223
26	C	-3.38707	1.007806	-0.727
27	H	-2.4308	1.59712	1.374758
28	C	-2.11146	-0.26141	2.354887
29	O	0.300146	1.158066	2.148882
30	H	-2.14958	-2.74872	0.933416
31	H	-1.71998	-3.49827	-0.61206
32	H	-0.58645	-3.56999	0.754432
33	H	-3.78806	0.468769	-1.58182
34	H	-2.99604	1.975472	-1.06305
35	H	-4.18167	1.176075	0.005042
36	H	-1.44209	-1.1232	2.344073
37	H	-1.95352	0.269716	3.295037
38	H	-3.14574	-0.61139	2.311925
39	H	0.411245	4.36487	0.128528
40	H	0.457512	3.794442	-1.5552
41	H	1.955621	4.294602	-0.74237

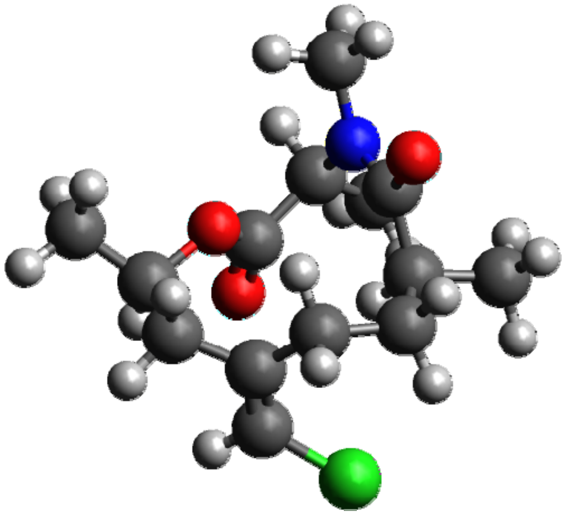

Table S18. Cartesian Coordinates For The Optimized Conformer 3 Of Compound 3*R*,6*S*,11*S*-**10t**

#	Atomic	Coordinates (Angstrom)		
		X	Y	Z
1	C	-0.53101	-1.8163	0.106514
2	C	-1.89374	-1.93971	-0.53035
3	C	-2.27355	-0.76487	-1.45601
4	C	-2.13717	0.619966	-0.79773
5	C	-0.71545	1.150881	-1.00991
6	N	-0.04934	1.777691	0.015347
7	C	1.403636	1.974292	-0.1523
8	C	2.006595	0.649009	0.318529
9	O	1.965706	-0.24132	-0.67328
10	C	2.017391	-1.65764	-0.40325
11	C	0.640393	-2.19902	-0.77961
12	C	-0.34378	-1.4646	1.380778
13	H	0.621717	-1.36372	1.861708
14	Cl	-1.64952	-1.14206	2.505538
15	C	3.120659	-2.24803	-1.26321
16	H	2.249332	-1.80627	0.655101
17	H	0.429372	-1.85992	-1.80247
18	H	0.68566	-3.29636	-0.81654
19	H	-1.91733	-2.86508	-1.1197
20	H	-2.6541	-2.03556	0.249057
21	H	-1.67786	-0.77624	-2.3737
22	H	-3.31819	-0.91069	-1.75296
23	C	-3.10847	1.633451	-1.41987
24	H	-2.36781	0.507808	0.263734
25	O	-0.20262	1.040519	-2.12096
26	C	-0.57434	1.991336	1.357833
27	H	1.58428	2.074841	-1.22272
28	C	1.956181	3.165908	0.609708
29	O	2.3658	0.413639	1.452261
30	H	-2.99505	2.625571	-0.971
31	H	-2.9186	1.720439	-2.49392
32	H	-4.14145	1.306558	-1.27363
33	H	-0.09205	1.325389	2.082896
34	H	-1.64666	1.820477	1.384778
35	H	-0.39715	3.026794	1.657567
36	H	1.913341	3.014097	1.690086
37	H	3.004018	3.308022	0.334262
38	H	1.404882	4.071901	0.34534
39	H	4.080402	-1.78846	-1.01626
40	H	2.910056	-2.07427	-2.32258
41	H	3.195232	-3.32505	-1.09151

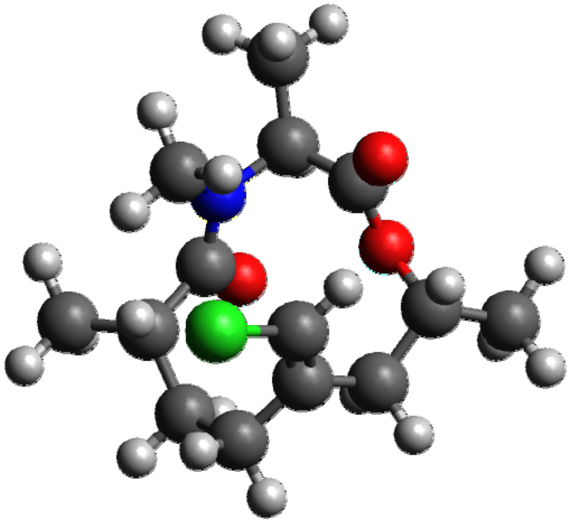

Table S19. Cartesian Coordinates For The Optimized Conformer 7 Of Compound 3*R*,6*S*,11*S*-**10t**

#	Atomic	Coordinates (Angstrom)		
		X	Y	Z
1	C	2.121243	-0.04568	-0.65084
2	C	1.63331	-1.11042	-1.6028
3	C	0.095796	-1.16675	-1.70575
4	C	-0.58393	-1.40853	-0.3473
5	C	-2.0776	-1.11692	-0.45365
6	N	-2.62999	-0.15282	0.345346
7	C	-1.85931	0.840898	1.090683
8	C	-0.82872	1.504728	0.168311
9	O	0.312908	1.785317	0.792936
10	C	1.425325	2.3467	0.041674
11	C	1.890725	1.400458	-1.06973
12	C	2.730033	-0.29415	0.508679
13	H	3.07402	0.475073	1.19057
14	Cl	3.070976	-1.89275	1.128435
15	C	1.116633	3.748168	-0.46246
16	H	2.196518	2.413893	0.813883
17	H	1.165991	1.428347	-1.88721
18	H	2.825747	1.814085	-1.46916
19	H	2.033767	-0.88955	-2.60065
20	H	2.029633	-2.08563	-1.3094
21	H	-0.27716	-0.23282	-2.13829
22	H	-0.1899	-1.96814	-2.39545
23	C	-0.41981	-2.85714	0.131548
24	H	-0.10861	-0.75608	0.388427
25	O	-2.77039	-1.78351	-1.22036
26	C	-4.06386	0.089457	0.23595
27	H	-2.56292	1.657368	1.300107
28	C	-1.33236	0.338094	2.433958
29	O	-1.06383	1.753777	-0.99276
30	H	-0.86257	-2.99552	1.123283
31	H	-0.91947	-3.5337	-0.56689
32	H	0.637407	-3.12439	0.195846
33	H	-4.55338	-0.82302	-0.09787
34	H	-4.27142	0.889351	-0.48471
35	H	-4.45429	0.375959	1.2158
36	H	-0.58099	-0.44623	2.316343
37	H	-0.88408	1.158462	2.997674
38	H	-2.16949	-0.06866	3.00688
39	H	0.726702	4.364201	0.351896
40	H	0.391613	3.728409	-1.27713
41	H	2.041778	4.206838	-0.82292

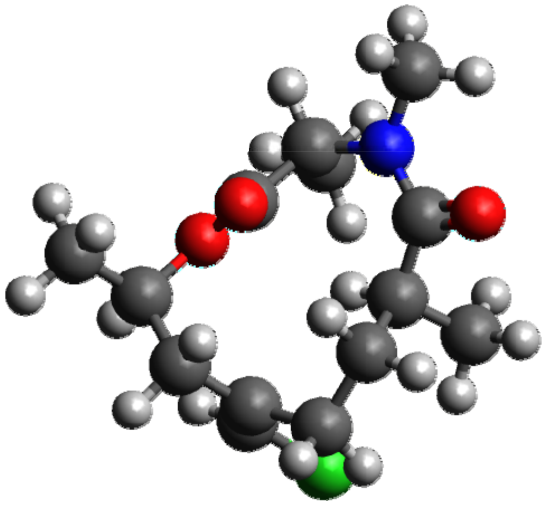

Table S20. Cartesian Coordinates For The Optimized Conformer 15 Of Compound 3*R*,6*S*,11*S*-**10t**

#	Atomic	Coordinates (Angstrom)		
		X	Y	Z
1	C	1.949068	-0.88156	-0.56791
2	C	1.219442	0.39333	-0.92768
3	C	1.094057	1.412734	0.220961
4	C	-0.08217	2.405051	0.04388
5	C	-1.33175	1.643596	0.485766
6	N	-2.0637	0.920787	-0.42711
7	C	-2.83348	-0.21422	0.090541
8	C	-1.92838	-1.45359	0.026683
9	O	-0.78435	-1.23514	0.687275
10	C	0.209962	-2.28531	0.720705
11	C	1.14644	-2.16329	-0.48851
12	C	3.26536	-0.94326	-0.36634
13	H	3.802976	-1.85299	-0.12239
14	Cl	4.320546	0.442743	-0.49927
15	C	0.919803	-2.16427	2.055078
16	H	-0.3149	-3.24262	0.649002
17	H	1.820943	-3.02619	-0.4578
18	H	0.542168	-2.26049	-1.39999
19	H	1.726338	0.875333	-1.77368
20	H	0.225169	0.108521	-1.27335
21	H	0.956832	0.895472	1.177378
22	H	2.023646	1.985061	0.29993
23	C	0.113286	3.643462	0.916728
24	H	-0.12764	2.717695	-1.00255
25	O	-1.59346	1.586069	1.683838
26	C	-1.99042	1.025924	-1.87755
27	H	-3.01164	-0.00815	1.148624
28	C	-4.15366	-0.44558	-0.62668
29	O	-2.20087	-2.48464	-0.54587
30	H	-0.74627	4.316177	0.850367
31	H	0.23468	3.353376	1.962857
32	H	1.005085	4.187268	0.593673
33	H	-1.69746	0.068004	-2.32672
34	H	-1.2674	1.778231	-2.17958
35	H	-2.96569	1.309504	-2.28355
36	H	-4.01113	-0.75767	-1.6636
37	H	-4.70603	-1.23864	-0.11892
38	H	-4.75154	0.468838	-0.60187
39	H	0.2099	-2.28896	2.875609
40	H	1.4045	-1.18952	2.153439
41	H	1.688083	-2.9389	2.134498

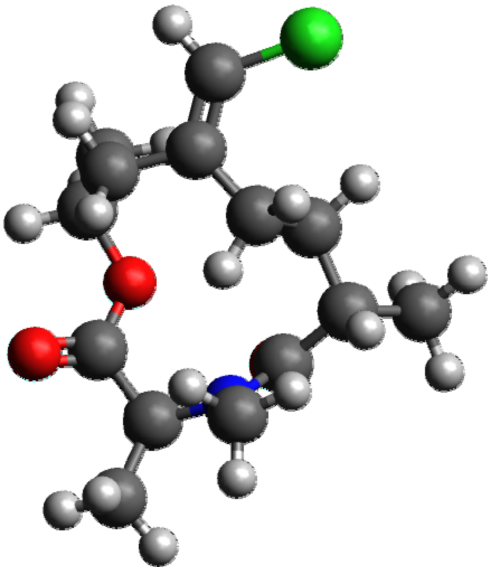

Table S21. Cartesian Coordinates For The Optimized Conformer 6 Of Compound *3R,6S,11S*-**10t**

#	Atomic	Coordinates (Angstrom)		
		X	Y	Z
1	C	2.459677	0.288308	-0.2821
2	C	1.563771	-0.6569	0.473881
3	C	0.495129	-1.3111	-0.42171
4	C	-0.80137	-1.64169	0.356926
5	C	-1.95642	-1.46858	-0.61946
6	N	-2.61217	-0.26463	-0.6431
7	C	-2.62889	0.694468	0.459767
8	C	-1.282	1.339409	0.800546
9	O	-0.48122	1.465359	-0.25745
10	C	0.76192	2.205962	-0.10004
11	C	1.816607	1.492738	-0.94797
12	C	3.780364	0.1476	-0.40058
13	H	4.413148	0.853727	-0.92712
14	Cl	4.693742	-1.18811	0.256992
15	C	0.507008	3.631473	-0.55884
16	H	1.033364	2.191124	0.959395
17	H	1.337675	1.198728	-1.8914
18	H	2.595357	2.216812	-1.20785
19	H	2.158368	-1.416	0.986868
20	H	1.063386	-0.07458	1.26038
21	H	0.250998	-0.63667	-1.24593
22	H	0.888627	-2.22781	-0.87426
23	C	-0.782	-3.05215	0.942659
24	H	-0.88956	-0.93397	1.185782
25	O	-2.20452	-2.35204	-1.43673
26	C	-3.64284	-0.0614	-1.6559
27	H	-3.21072	1.548361	0.086286
28	C	-3.33613	0.164679	1.707297
29	O	-1.01928	1.746905	1.910416
30	H	-1.66558	-3.24139	1.559852
31	H	-0.76291	-3.78836	0.135789
32	H	0.106627	-3.18614	1.566616
33	H	-4.64573	-0.19107	-1.23086
34	H	-3.50266	-0.78934	-2.45149
35	H	-3.55495	0.949654	-2.06193
36	H	-2.78568	-0.66767	2.15413
37	H	-3.43222	0.952144	2.45605
38	H	-4.3322	-0.18814	1.429258
39	H	-0.30615	4.077013	0.020074
40	H	0.238327	3.647555	-1.6194
41	H	1.40515	4.237469	-0.41412

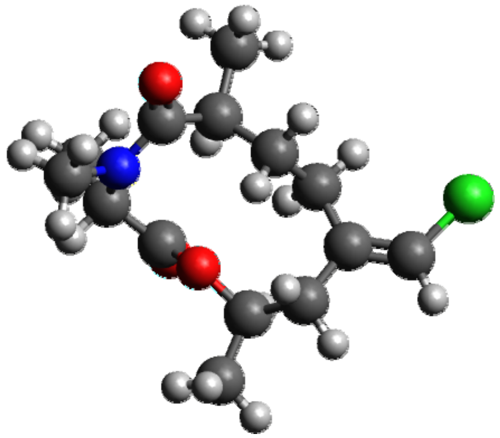

Table S22. Cartesian Coordinates For The Optimized Conformer 13 Of Compound 3*R*,6*S*,11*S*-**10t**

#	Atomic	Coordinates (Angstrom)		
		X	Y	Z
1	C	2.354559	-0.31202	-0.58368
2	C	1.653621	0.955864	-1.00312
3	C	0.581177	1.424518	0.01155
4	C	-0.83691	1.433762	-0.59419
5	C	-1.82719	1.421713	0.561863
6	N	-2.55467	0.289741	0.80344
7	C	-2.69957	-0.82564	-0.12537
8	C	-1.40909	-1.57206	-0.46944
9	O	-0.44001	-1.40227	0.42827
10	C	0.764282	-2.19621	0.266862
11	C	1.680174	-1.64682	-0.84094
12	C	3.555148	-0.32421	-0.00167
13	H	4.079054	-1.23132	0.280887
14	Cl	4.459218	1.112862	0.406455
15	C	1.40405	-2.25054	1.639624
16	H	0.445889	-3.19545	-0.04757
17	H	2.448294	-2.41518	-0.98743
18	H	1.103992	-1.59766	-1.77088
19	H	2.395306	1.742537	-1.1572
20	H	1.183453	0.774435	-1.97832
21	H	0.59716	0.774655	0.89149
22	H	0.807841	2.434033	0.368636
23	C	-1.0603	2.666905	-1.47163
24	H	-0.94298	0.545343	-1.2227
25	O	-1.89857	2.405264	1.297282
26	C	-3.44818	0.261867	1.9562
27	H	-3.27198	-1.58572	0.423825
28	C	-3.49869	-0.46976	-1.38057
29	O	-1.32342	-2.29669	-1.43664
30	H	-2.03598	2.638944	-1.96675
31	H	-1.01183	3.568835	-0.85649
32	H	-0.28814	2.725893	-2.24489
33	H	-4.49615	0.274098	1.634531
34	H	-3.25479	1.137695	2.570779
35	H	-3.26521	-0.64507	2.539772
36	H	-2.96852	0.26421	-1.99333
37	H	-3.6735	-1.36113	-1.98453
38	H	-4.45976	-0.04331	-1.08344
39	H	0.720401	-2.71327	2.354782
40	H	1.660315	-1.24819	1.992443
41	H	2.320729	-2.84579	1.598394

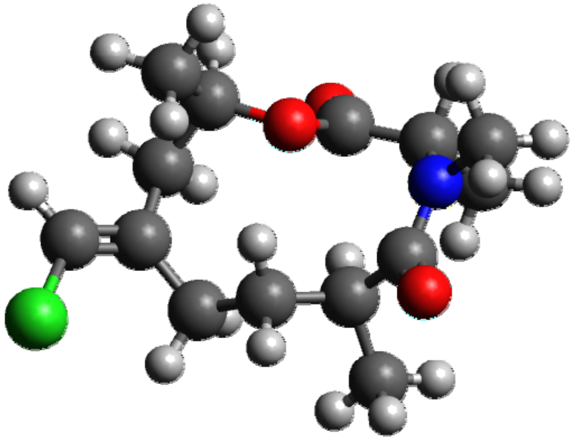

Table S23. Cartesian Coordinates For The Optimized Conformer 9 Of Compound 3*R*,6*S*,11*S*-**10t**

#	Atomic	Coordinates (Angstrom)		
		X	Y	Z
1	C	-1.82488	1.136109	-0.18854
2	C	-1.64782	0.50909	-1.55621
3	C	-1.22668	-0.9704	-1.63735
4	C	0.17032	-1.35854	-1.13459
5	C	0.210113	-1.51985	0.386535
6	N	1.371581	-1.24078	1.058548
7	C	2.48832	-0.46227	0.531484
8	C	2.054982	0.841311	-0.13278
9	O	0.938359	1.32688	0.407899
10	C	0.360943	2.496684	-0.21094
11	C	-1.12617	2.448846	0.109688
12	C	-2.68846	0.70086	0.729532
13	H	-2.81719	1.173997	1.696557
14	Cl	-3.79255	-0.62637	0.496224
15	C	1.032452	3.752091	0.318447
16	H	0.534214	2.411714	-1.2887
17	H	-1.27392	2.712748	1.163132
18	H	-1.60298	3.2402	-0.48618
19	H	-2.60807	0.594076	-2.08322
20	H	-0.93572	1.107568	-2.13549
21	H	-1.94947	-1.60295	-1.11787
22	H	-1.27096	-1.24143	-2.69897
23	C	0.533002	-2.74205	-1.7119
24	H	0.904704	-0.62987	-1.49467
25	O	-0.74945	-2.01985	0.966459
26	C	1.410755	-1.51836	2.489906
27	H	3.04942	-0.12589	1.41384
28	C	3.452311	-1.24586	-0.35753
29	O	2.670833	1.373784	-1.02889
30	H	1.46225	-3.13387	-1.28965
31	H	-0.26789	-3.45135	-1.48196
32	H	0.642913	-2.68147	-2.79824
33	H	0.704036	-2.31312	2.71799
34	H	1.141535	-0.62684	3.069689
35	H	2.41943	-1.83614	2.766273
36	H	3.001636	-1.48893	-1.32201
37	H	4.350243	-0.65474	-0.54578
38	H	3.732384	-2.17381	0.146903
39	H	2.092517	3.759777	0.056842
40	H	0.928985	3.804582	1.406275
41	H	0.561035	4.636925	-0.11861

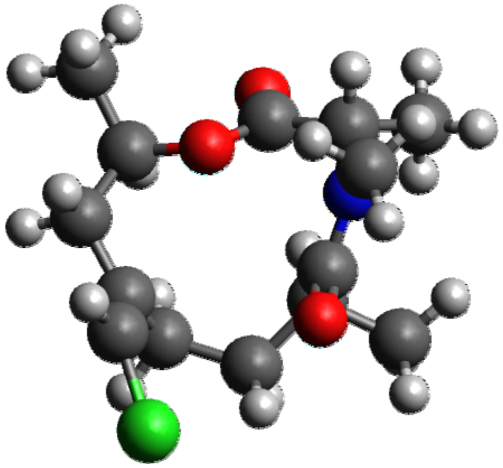

Table S24. Cartesian Coordinates For The Optimized Conformer 10 Of Compound 3*R*,6*S*,11*S*-**10t**

#	Atomic	Coordinates (Angstrom)		
		X	Y	Z
1	C	-2.25326	0.48933	0.52125
2	C	-1.28745	-0.66694	0.594867
3	C	-0.67236	-0.93275	-0.79661
4	C	0.701406	-1.66472	-0.82938
5	C	1.411496	-1.60531	0.524447
6	N	2.276957	-0.57965	0.794031
7	C	2.661646	0.492673	-0.12211
8	C	1.475717	1.279829	-0.67913
9	O	0.586792	1.554502	0.27886
10	C	-0.57891	2.345469	-0.06793
11	C	-1.72132	1.871693	0.83359
12	C	-3.53355	0.364541	0.171868
13	H	-4.22968	1.192694	0.098198
14	Cl	-4.27589	-1.16998	-0.21183
15	C	-0.24077	3.811841	0.14222
16	H	-0.81442	2.159663	-1.12066
17	H	-1.3594	1.896364	1.870759
18	H	-2.52968	2.606282	0.75357
19	H	-0.50904	-0.41013	1.317783
20	H	-1.78665	-1.56812	0.962786
21	H	-1.38675	-1.52412	-1.37961
22	H	-0.57982	0.013484	-1.33429
23	C	0.531523	-3.13426	-1.2251
24	H	1.312443	-1.1756	-1.59262
25	O	1.171699	-2.46657	1.368221
26	C	2.910559	-0.52539	2.107714
27	H	3.162534	1.235755	0.512977
28	C	3.657275	0.071963	-1.19932
29	O	1.387386	1.652388	-1.82658
30	H	1.491573	-3.65764	-1.24264
31	H	-0.1181	-3.64349	-0.50935
32	H	0.082919	-3.19599	-2.22015
33	H	2.687437	-1.4426	2.646657
34	H	2.531818	0.332391	2.674531
35	H	3.994462	-0.42502	1.991367
36	H	3.22558	-0.63979	-1.90515
37	H	3.993405	0.945472	-1.76138
38	H	4.520792	-0.39509	-0.71946
39	H	0.616235	4.095231	-0.47382
40	H	-0.00245	3.999666	1.193468
41	H	-1.09084	4.43734	-0.14235

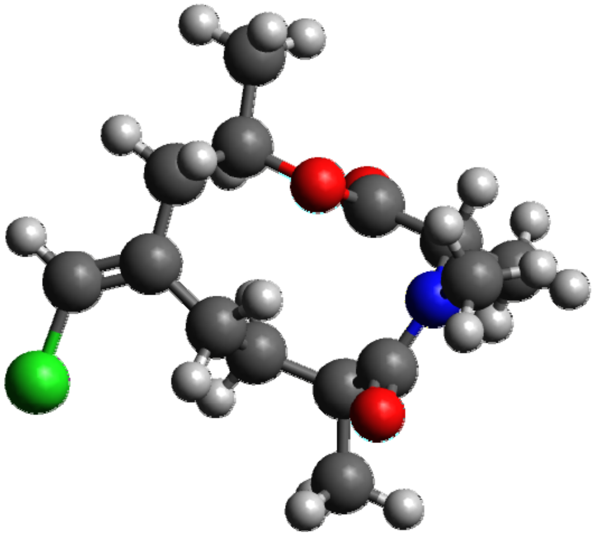

Table S25. Boltzmann Distribution of Conformers of (3R,6*S*,11*R*)-**10t**

Conformer	Energy (kcal/mol)	Relative Energy (kcal/mol)	Boltzmann Factor	Equil Mole Fraction	Imaginary Frequency
10	-807441	0.00	1.000	0.262	0
21	-807441	0.00	0.999	0.262	0
3	-807440	0.25	0.655	0.172	0
13	-807440	0.58	0.375	0.098	0
5	-807440	0.83	0.248	0.065	0
16	-807440	0.83	0.246	0.065	0
12	-807440	0.93	0.207	0.054	0
8	-807439	1.63	0.063	0.017	0
4	-807437	3.23	0.004	0.001	0
18	-807437	3.25	0.004	0.001	0
9	-807437	3.33	0.004	0.001	0
19	-807437	3.38	0.003	0.001	0
15	-807437	3.39	0.003	0.001	0
11	-807437	3.48	0.003	0.001	0
7	-807437	3.56	0.002	0.001	0
23	-807436	4.51	0.000	0.000	0
17	-807434	6.10	0.000	0.000	0

XYZ coordinates listed in following tables for any conformer with an equilibrium mole fraction greater than or equal to 0.001

Table S26. Cartesian Coordinates For The Optimized Conformer 10 Of Compound 3*R*,6*S*,11*R*-**10t**

#	Atomic	Coordinates (Angstrom)		
		X	Y	Z
1	C	2.105104	0.695055	-0.53983
2	C	1.405083	-0.64108	-0.64907
3	C	0.816574	-1.12341	0.691565
4	C	-0.23879	-2.24364	0.541577
5	C	-1.27158	-1.89262	-0.53802
6	N	-2.16082	-0.87521	-0.31507
7	C	-2.26136	-0.10493	0.909521
8	C	-1.52662	1.240634	0.833205
9	O	-0.73001	1.336355	-0.23164
10	C	0.227955	2.421723	-0.25906
11	C	1.411984	1.926119	-1.08799
12	C	3.318105	0.844182	-0.00625
13	H	3.838455	1.792616	0.069705
14	Cl	4.258917	-0.48561	0.624449
15	C	-0.42591	3.658417	-0.85004
16	H	0.542843	2.608396	0.773826
17	H	1.056246	1.713556	-2.10508
18	H	2.12452	2.755002	-1.1589
19	H	0.607441	-0.54963	-1.3925
20	H	2.111774	-1.38991	-1.02473
21	H	1.624324	-1.50514	1.325041
22	H	0.39884	-0.2687	1.22976
23	C	0.419945	-3.58087	0.203468
24	H	-0.75194	-2.35512	1.505339
25	O	-1.27317	-2.49591	-1.6089
26	C	-2.99476	-0.43278	-1.42746
27	H	-1.74425	-0.64644	1.707164
28	C	-3.70832	0.098721	1.365607
29	O	-1.61756	2.083479	1.697682
30	H	-0.32408	-4.37523	0.111515
31	H	0.956542	-3.52414	-0.74629
32	H	1.126102	-3.84999	0.993985
33	H	-4.00879	-0.83902	-1.35447
34	H	-2.54493	-0.77309	-2.3579
35	H	-3.04549	0.659923	-1.4298
36	H	-4.27653	0.700607	0.652432
37	H	-4.19599	-0.87254	1.475211
38	H	-3.71452	0.615151	2.326302
39	H	-1.27821	3.965261	-0.24015
40	H	-0.76853	3.453091	-1.86877
41	H	0.291765	4.482659	-0.88229

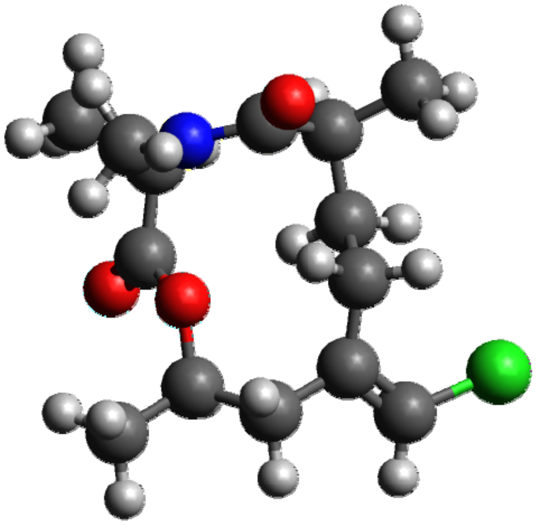

Table S27. Cartesian Coordinates For The Optimized Conformer 21 Of Compound 3*R*,6*S*,11*R*-**10t**

#	Atomic	Coordinates (Angstrom)		
		X	Y	Z
1	C	2.104997	0.695049	-0.53978
2	C	1.405023	-0.64111	-0.649
3	C	0.816501	-1.1235	0.69164
4	C	-0.23882	-2.24372	0.541522
5	C	-1.27168	-1.89266	-0.53803
6	N	-2.16084	-0.87518	-0.31505
7	C	-2.26128	-0.10494	0.909575
8	C	-1.52641	1.240572	0.833381
9	O	-0.73009	1.336478	-0.23167
10	C	0.227923	2.421782	-0.25916
11	C	1.411952	1.926087	-1.08803
12	C	3.318027	0.844144	-0.00623
13	H	3.838365	1.792596	0.069624
14	Cl	4.258867	-0.48561	0.624379
15	C	-0.42593	3.658448	-0.85026
16	H	0.542794	2.608572	0.77371
17	H	1.0562	1.713501	-2.10511
18	H	2.124504	2.754951	-1.15895
19	H	0.607338	-0.5497	-1.39238
20	H	2.111742	-1.38989	-1.02468
21	H	1.624221	-1.50528	1.325121
22	H	0.39878	-0.26879	1.229855
23	C	0.420014	-3.58091	0.20332
24	H	-0.752	-2.35532	1.505259
25	O	-1.27339	-2.49592	-1.60893
26	C	-2.99472	-0.43264	-1.42744
27	H	-1.74428	-0.64655	1.707216
28	C	-3.70822	0.098917	1.365617
29	O	-1.61712	2.083242	1.698058
30	H	-0.32395	-4.37531	0.111293
31	H	0.956589	-3.52404	-0.74644
32	H	1.126205	-3.85001	0.993808
33	H	-4.00871	-0.83903	-1.35462
34	H	-2.54477	-0.77275	-2.35791
35	H	-3.04562	0.66005	-1.42953
36	H	-4.27636	0.700807	0.652387
37	H	-4.19602	-0.87226	1.475354
38	H	-3.71433	0.615443	2.326265
39	H	-1.27818	3.965369	-0.24033
40	H	-0.76866	3.452963	-1.86892
41	H	0.291754	4.482666	-0.88268

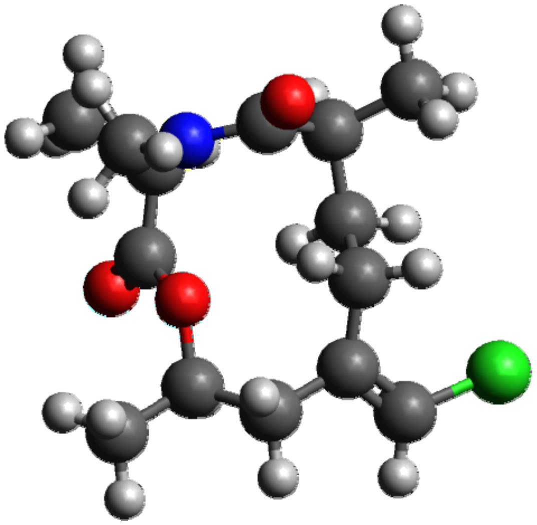

Table S28. Cartesian Coordinates For The Optimized Conformer 3 Of Compound 3*R*,6*S*,11*R*-**10t**

#	Atomic	Coordinates (Angstrom)		
		X	Y	Z
1	C	2.231234	0.403024	-0.58351
2	C	2.201269	-0.93587	-1.27526
3	C	0.767352	-1.42279	-1.534
4	C	0.008959	-1.7729	-0.23573
5	C	-1.4751	-1.65883	-0.56703
6	N	-2.23908	-0.69842	0.039741
7	C	-1.81053	0.101286	1.178534
8	C	-1.09496	1.397738	0.776713
9	O	-0.42744	1.269214	-0.37206
10	C	0.530948	2.302376	-0.70673
11	C	1.708401	1.593474	-1.37517
12	C	2.642119	0.597796	0.669378
13	H	2.66451	1.567183	1.156913
14	Cl	3.19506	-0.6797	1.727726
15	C	-0.13316	3.333381	-1.59985
16	H	0.850557	2.766637	0.230928
17	H	1.400882	1.255481	-2.37137
18	H	2.502383	2.334063	-1.5255
19	H	2.712521	-0.83027	-2.24071
20	H	2.757534	-1.67858	-0.69721
21	H	0.217346	-0.64816	-2.08013
22	H	0.786236	-2.30575	-2.18138
23	C	0.342349	-3.18229	0.257435
24	H	0.310327	-1.04818	0.522362
25	O	-1.94721	-2.38808	-1.43845
26	C	-3.53466	-0.37049	-0.54438
27	H	-1.05263	-0.46631	1.729043
28	C	-2.94534	0.388533	2.159732
29	O	-1.07379	2.389076	1.471212
30	H	-0.13867	-3.3931	1.217632
31	H	0.001062	-3.92262	-0.47114
32	H	1.422517	-3.29134	0.3931
33	H	-4.36115	-0.71198	0.085892
34	H	-3.60561	-0.85844	-1.51378
35	H	-3.61268	0.714525	-0.67454
36	H	-3.70225	1.047778	1.729316
37	H	-3.41687	-0.55017	2.459359
38	H	-2.53804	0.880681	3.043406
39	H	-0.96028	3.815989	-1.07399
40	H	-0.51648	2.856636	-2.50689
41	H	0.590584	4.101275	-1.88731

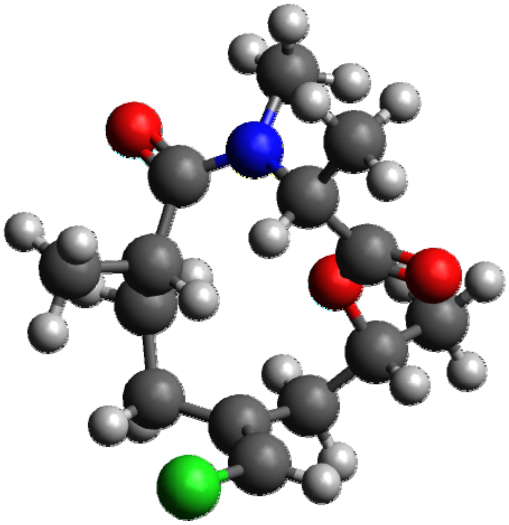

Table S29. Cartesian Coordinates For The Optimized Conformer 13 Of Compound 3*R*,6*S*,11*R*-**10t**

#	Atomic	Coordinates (Angstrom)		
		X	Y	Z
1	C	2.111911	-0.60163	-0.30658
2	C	1.29154	0.63343	-0.01887
3	C	0.384763	1.041028	-1.19156
4	C	-0.67763	2.104062	-0.82722
5	C	-1.39292	1.760047	0.487529
6	N	-2.26246	0.699859	0.526498
7	C	-2.53846	-0.18139	-0.59323
8	C	-1.62728	-1.41713	-0.63068
9	O	-0.73904	-1.43994	0.361436
10	C	0.352851	-2.39081	0.280422
11	C	1.40128	-1.87869	-0.7161
12	C	3.442821	-0.64854	-0.21798
13	H	4.023408	-1.54477	-0.40719
14	Cl	4.458494	0.708657	0.204614
15	C	0.878635	-2.55361	1.692287
16	H	-0.05405	-3.33385	-0.09703
17	H	2.137969	-2.67816	-0.8499
18	H	0.912755	-1.74659	-1.68891
19	H	0.672062	0.429155	0.862949
20	H	1.951679	1.46242	0.245376
21	H	1.00368	1.446811	-2.00067
22	H	-0.10105	0.157879	-1.61406
23	C	-0.05313	3.494953	-0.71734
24	H	-1.41807	2.13044	-1.63689
25	O	-1.16903	2.424348	1.496336
26	C	-2.8025	0.29542	1.821099
27	H	-2.31342	0.349612	-1.52235
28	C	-4.00745	-0.60877	-0.66869
29	O	-1.69405	-2.24554	-1.51222
30	H	-0.8058	4.246231	-0.46879
31	H	0.70781	3.522879	0.065794
32	H	0.409726	3.763146	-1.67118
33	H	-3.86682	0.538245	1.897396
34	H	-2.26072	0.825121	2.601174
35	H	-2.66696	-0.78168	1.955564
36	H	-4.29774	-1.22146	0.18789
37	H	-4.64518	0.277524	-0.70159
38	H	-4.16179	-1.19486	-1.57586
39	H	0.096345	-2.94045	2.349196
40	H	1.233077	-1.59843	2.089823
41	H	1.716245	-3.25656	1.69231

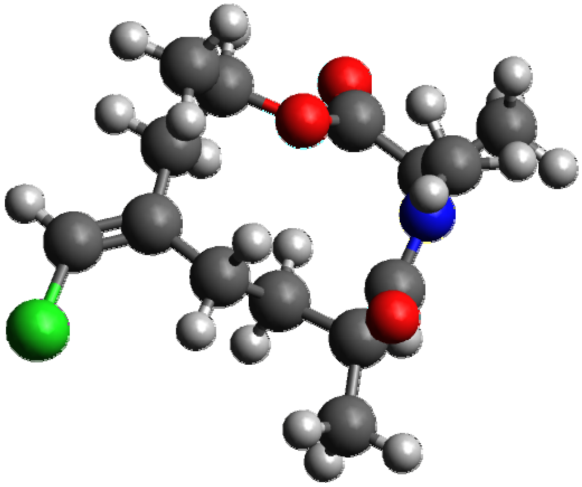

Table S30. Cartesian Coordinates For The Optimized Conformer 5 Of Compound 3*R*,6*S*,11*R*-**10t**

#	Atomic	Coordinates (Angstrom)		
		X	Y	Z
1	C	2.069782	0.663635	-0.63416
2	C	1.86245	-0.65185	-1.36293
3	C	1.455685	-1.89154	-0.54861
4	C	0.143012	-1.76681	0.225525
5	C	-1.03128	-1.58078	-0.73833
6	N	-2.17272	-0.97065	-0.27717
7	C	-2.19969	-0.2374	0.979621
8	C	-1.47928	1.114677	0.892094
9	O	-0.70858	1.207466	-0.19241
10	C	0.172703	2.349737	-0.2915
11	C	1.343823	1.89852	-1.15693
12	C	2.917102	0.861733	0.376574
13	H	3.077642	1.831858	0.836198
14	Cl	3.942752	-0.36468	1.07837
15	C	-0.58486	3.526312	-0.88081
16	H	0.519734	2.58397	0.720806
17	H	0.9771	1.702446	-2.17073
18	H	2.037199	2.743849	-1.2246
19	H	1.109323	-0.49906	-2.14143
20	H	2.800196	-0.89123	-1.88324
21	H	1.363473	-2.7247	-1.25282
22	H	2.239786	-2.15466	0.164855
23	C	-0.11205	-3.03577	1.055231
24	H	0.234345	-0.90879	0.898688
25	O	-0.96731	-2.03576	-1.87744
26	C	-3.20949	-0.64053	-1.24855
27	H	-1.63498	-0.80354	1.727171
28	C	-3.60957	-0.05422	1.539777
29	O	-1.56083	1.965838	1.749047
30	H	-1.04598	-2.97746	1.623231
31	H	-0.17393	-3.90656	0.394873
32	H	0.708503	-3.19558	1.760114
33	H	-4.19332	-0.93497	-0.87798
34	H	-2.99625	-1.17836	-2.16878
35	H	-3.21087	0.43752	-1.45596
36	H	-4.2186	0.598983	0.910942
37	H	-4.10085	-1.02618	1.626346
38	H	-3.53958	0.39941	2.528909
39	H	-1.41873	3.806206	-0.23349
40	H	-0.96989	3.268437	-1.87199
41	H	0.081593	4.387708	-0.97879

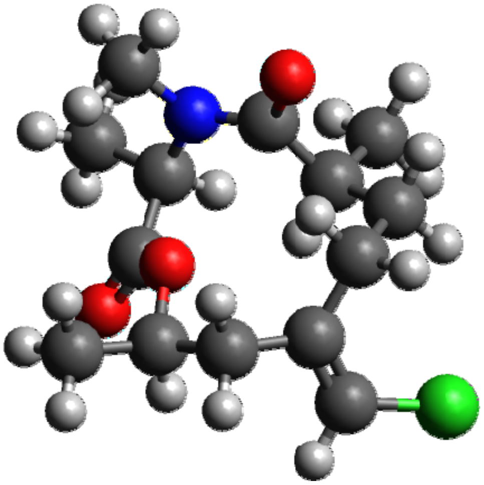

Table S31. Cartesian Coordinates For The Optimized Conformer 16 Of Compound 3*R*,6*S*,11*R*-**10t**

#	Atomic	Coordinates (Angstrom)		
		X	Y	Z
1	C	2.070041	0.663231	-0.63422
2	C	1.862551	-0.65239	-1.36272
3	C	1.455364	-1.89182	-0.54821
4	C	0.142625	-1.76666	0.225731
5	C	-1.03154	-1.58043	-0.73828
6	N	-2.17305	-0.97048	-0.27717
7	C	-2.20006	-0.2371	0.979554
8	C	-1.47923	1.114771	0.892065
9	O	-0.70873	1.207532	-0.19257
10	C	0.173007	2.349462	-0.29158
11	C	1.344005	1.89802	-1.15706
12	C	2.91753	0.861483	0.37636
13	H	3.078186	1.831655	0.835832
14	Cl	3.943305	-0.36485	1.078195
15	C	-0.58416	3.526356	-0.88078
16	H	0.520134	2.583539	0.720717
17	H	0.977245	1.70184	-2.17083
18	H	2.03741	2.74331	-1.22486
19	H	1.109568	-0.49961	-2.14135
20	H	2.800327	-0.89207	-1.88281
21	H	1.36311	-2.7251	-1.25227
22	H	2.23929	-2.15499	0.16543
23	C	-0.11289	-3.03548	1.055514
24	H	0.23416	-0.90863	0.898859
25	O	-0.96745	-2.03548	-1.87734
26	C	-3.20984	-0.64054	-1.2486
27	H	-1.63573	-0.80339	1.72728
28	C	-3.60999	-0.05335	1.53945
29	O	-1.56037	1.96581	1.749195
30	H	-1.04679	-2.9768	1.623513
31	H	-0.17509	-3.9063	0.395216
32	H	0.707591	-3.19559	1.760414
33	H	-4.19366	-0.93464	-0.87778
34	H	-2.99679	-1.17881	-2.16862
35	H	-3.21102	0.437421	-1.45649
36	H	-4.21857	0.600343	0.910691
37	H	-4.10182	-1.02507	1.625638
38	H	-3.53993	0.399933	2.528733
39	H	-1.41795	3.806497	-0.23345
40	H	-0.96929	3.268679	-1.87197
41	H	0.082572	4.387541	-0.9787

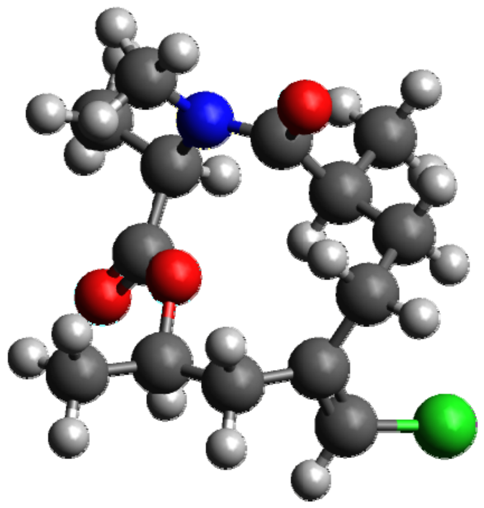

Table S32. Cartesian Coordinates For The Optimized Conformer 12 Of Compound 3*R*,6*S*,11*R*-**10t**

#	Atomic	Coordinates (Angstrom)		
		X	Y	Z
1	C	2.141678	-0.80399	-0.62895
2	C	1.47415	0.51611	-0.96304
3	C	1.235709	1.452756	0.247372
4	C	-0.07564	2.255744	0.148457
5	C	-1.21881	1.267456	0.362002
6	N	-2.06026	0.956848	-0.66351
7	C	-2.89042	-0.23687	-0.50286
8	C	-2.06059	-1.42378	-0.00494
9	O	-0.80787	-1.35982	-0.46065
10	C	0.157392	-2.33957	-0.02123
11	C	1.397721	-2.10026	-0.88773
12	C	3.388668	-0.8901	-0.16462
13	H	3.888249	-1.82301	0.071436
14	Cl	4.413611	0.499944	0.100065
15	C	0.428699	-2.20843	1.468151
16	H	-0.24915	-3.33206	-0.24911
17	H	2.073851	-2.94768	-0.73284
18	H	1.084241	-2.12349	-1.93944
19	H	2.094453	1.043407	-1.69896
20	H	0.521255	0.2971	-1.45074
21	H	1.209268	0.881082	1.181191
22	H	2.065487	2.159818	0.336355
23	C	-0.13429	3.346951	1.219714
24	H	-0.12083	2.715325	-0.84267
25	O	-1.3233	0.687989	1.444194
26	C	-2.09303	1.622904	-1.95902
27	H	-3.20589	-0.52973	-1.5133
28	C	-4.12553	-0.00609	0.360089
29	O	-2.51259	-2.34401	0.635622
30	H	-1.06818	3.914138	1.161793
31	H	-0.06978	2.895732	2.213426
32	H	0.699235	4.043325	1.094495
33	H	-1.40133	1.163188	-2.67619
34	H	-1.85891	2.68147	-1.85835
35	H	-3.10733	1.552323	-2.35844
36	H	-3.8239	0.277059	1.369596
37	H	-4.72923	0.794075	-0.07582
38	H	-4.72496	-0.91703	0.40977
39	H	-0.45143	-2.4841	2.04995
40	H	0.703065	-1.18023	1.715572
41	H	1.258078	-2.86801	1.742096

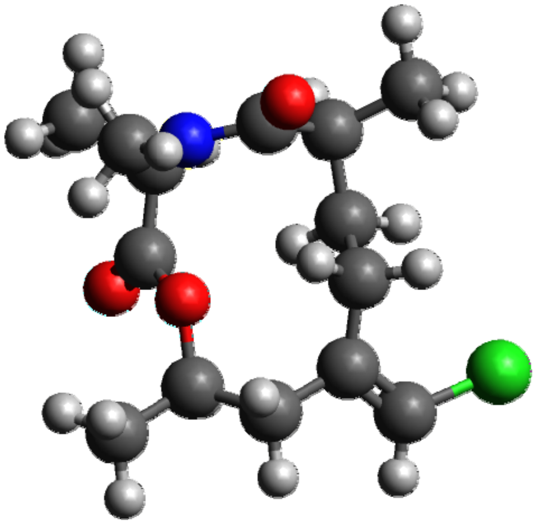

Table S33. Cartesian Coordinates For The Optimized Conformer 8 Of Compound 3*R*,6*S*,11*R*-**10t**

#	Atomic	Coordinates (Angstrom)		
		X	Y	Z
1	C	-2.20622	0.454741	-0.22459
2	C	-1.5723	-0.7103	0.497717
3	C	-0.98131	-1.80586	-0.41967
4	C	0.496768	-1.61599	-0.78389
5	C	1.323981	-1.5676	0.505873
6	N	2.400384	-0.7226	0.581735
7	C	2.720378	0.208195	-0.49048
8	C	1.711403	1.352557	-0.63354
9	O	0.775188	1.362548	0.317321
10	C	-0.36261	2.238826	0.100329
11	C	-1.35011	1.54065	-0.84508
12	C	-3.52708	0.578077	-0.37777
13	H	-3.99764	1.410296	-0.88969
14	Cl	-4.70058	-0.58431	0.19266
15	C	-0.93157	2.58911	1.460185
16	H	0.010824	3.137419	-0.3975
17	H	-2.00213	2.313134	-1.26735
18	H	-0.77761	1.132587	-1.68891
19	H	-0.78592	-0.34366	1.16508
20	H	-2.32966	-1.16736	1.138018
21	H	-1.0717	-2.76805	0.092645
22	H	-1.56478	-1.87363	-1.34574
23	C	1.003067	-2.7809	-1.64612
24	H	0.5952	-0.69109	-1.35805
25	O	1.020964	-2.30868	1.437815
26	C	3.015052	-0.50833	1.887851
27	H	2.661687	-0.32414	-1.44578
28	C	4.138203	0.776543	-0.39828
29	O	1.756357	2.129853	-1.56168
30	H	2.051134	-2.64882	-1.93359
31	H	0.917723	-3.71878	-1.08922
32	H	0.406272	-2.86326	-2.55866
33	H	4.099215	-0.62516	1.832994
34	H	2.610553	-1.24824	2.573894
35	H	2.777351	0.495245	2.25951
36	H	4.276987	1.39205	0.493284
37	H	4.866593	-0.03721	-0.38605
38	H	4.318517	1.397597	-1.2769
39	H	-0.18637	3.115949	2.06036
40	H	-1.25538	1.697249	2.002217
41	H	-1.79978	3.241284	1.329293

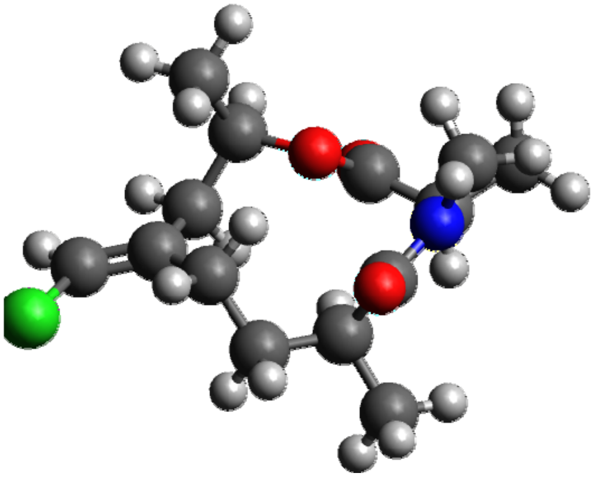

Table S34. Cartesian Coordinates For The Optimized Conformer 4 Of Compound 3*R*,6*S*,11*R*-**10t**

#	Atomic	Coordinates (Angstrom)		
		X	Y	Z
1	C	2.137028	0.056216	-0.55905
2	C	1.797926	-1.1027	-1.46394
3	C	0.278262	-1.33975	-1.56862
4	C	-0.37249	-1.73353	-0.22284
5	C	-1.87385	-1.47002	-0.33337
6	N	-2.43537	-0.41459	0.3465
7	C	-1.62752	0.553367	1.082638
8	C	-0.80823	1.406107	0.110651
9	O	0.276701	1.904026	0.707741
10	C	1.340407	2.474403	-0.10225
11	C	1.867021	1.448463	-1.11232
12	C	2.62364	-0.07982	0.674542
13	H	2.843162	0.747251	1.339578
14	Cl	2.962557	-1.61885	1.430876
15	C	0.929245	3.788285	-0.74785
16	H	2.107337	2.677391	0.650725
17	H	1.157926	1.369223	-1.94153
18	H	2.793749	1.856277	-1.53653
19	H	2.180445	-0.88323	-2.46848
20	H	2.299702	-2.01115	-1.12036
21	H	-0.2	-0.43843	-1.96569
22	H	0.086131	-2.13903	-2.29297
23	C	-0.13601	-3.21089	0.102578
24	H	0.082583	-1.13071	0.566073
25	O	-2.55124	-2.2017	-1.05225
26	C	-3.80626	-0.04377	0.003628
27	H	-0.9198	0.021589	1.722438
28	C	-2.45125	1.464294	1.997954
29	O	-1.10571	1.582322	-1.04897
30	H	-0.53098	-3.46661	1.090332
31	H	-0.63291	-3.8377	-0.64179
32	H	0.934417	-3.43274	0.10161
33	H	-4.39726	0.123002	0.90597
34	H	-4.24297	-0.86257	-0.5622
35	H	-3.81371	0.862844	-0.61126
36	H	-3.13085	2.107113	1.434059
37	H	-3.03027	0.863748	2.703171
38	H	-1.7664	2.098255	2.565004
39	H	0.50695	4.460712	0.003142
40	H	0.196096	3.628959	-1.53961
41	H	1.815185	4.267301	-1.17477

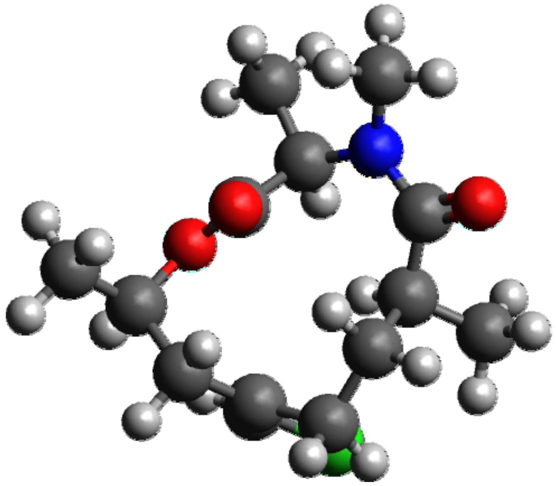

Table S35. Cartesian Coordinates For The Optimized Conformer 18 Of Compound 3*R*,6*S*,11*R*-**10t**

#	Atomic	Coordinates (Angstrom)		
		X	Y	Z
1	C	2.18469	-0.72874	-0.45161
2	C	1.323338	0.361683	-1.04847
3	C	0.726655	1.312515	0.008391
4	C	-0.54387	2.07255	-0.47542
5	C	-1.6889	1.712066	0.474628
6	N	-2.39525	0.55217	0.287067
7	C	-2.26903	-0.34961	-0.84313
8	C	-1.38064	-1.56649	-0.55192
9	O	-0.5579	-1.3642	0.475914
10	C	0.510554	-2.31877	0.693016
11	C	1.610512	-2.12715	-0.36225
12	C	3.429925	-0.52058	-0.02303
13	H	4.062859	-1.29004	0.405517
14	Cl	4.228595	1.029529	-0.11666
15	C	0.985769	-2.0938	2.114451
16	H	0.090663	-3.32307	0.576364
17	H	2.403272	-2.84592	-0.12766
18	H	1.203194	-2.40511	-1.34139
19	H	1.911672	0.946715	-1.76568
20	H	0.52246	-0.11379	-1.62525
21	H	0.492909	0.743077	0.911349
22	H	1.488474	2.044587	0.294662
23	C	-0.32991	3.585222	-0.48291
24	H	-0.77546	1.766042	-1.50023
25	O	-1.91426	2.425981	1.449548
26	C	-3.27974	0.107872	1.359427
27	H	-1.74514	0.172709	-1.64896
28	C	-3.62341	-0.79275	-1.40014
29	O	-1.38537	-2.56417	-1.23859
30	H	-1.22031	4.112986	-0.8355
31	H	-0.1032	3.940327	0.52454
32	H	0.506416	3.832441	-1.14353
33	H	-4.32762	0.332605	1.136266
34	H	-2.99785	0.617736	2.278119
35	H	-3.16773	-0.97246	1.495103
36	H	-4.17396	-1.40694	-0.68374
37	H	-4.22098	0.088269	-1.64473
38	H	-3.46888	-1.38515	-2.30258
39	H	0.160635	-2.22983	2.817175
40	H	1.390265	-1.08506	2.235667
41	H	1.773479	-2.8124	2.356979

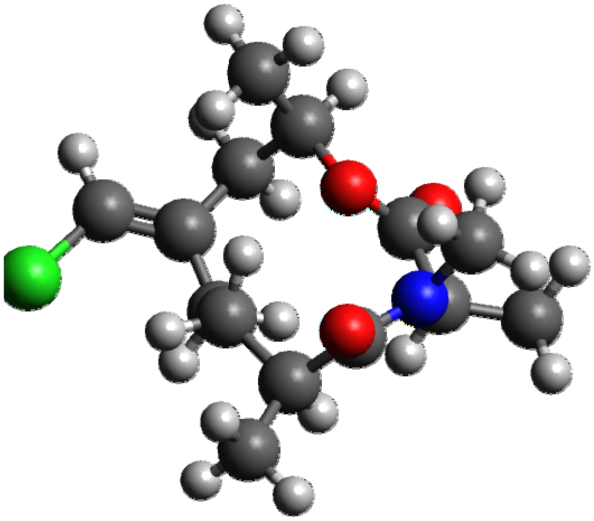

Table S36. Cartesian Coordinates For The Optimized Conformer 9 Of Compound 3*R*,6*S*,11*R*-**10t**

#	Atomic	Coordinates (Angstrom)		
		X	Y	Z
1	C	2.42194	-0.6922	-0.56482
2	C	1.765421	0.560026	-1.09828
3	C	0.842363	1.237994	-0.05969
4	C	-0.43065	1.845895	-0.69753
5	C	-1.5094	1.798429	0.377168
6	N	-2.34843	0.712165	0.440576
7	C	-2.56721	-0.26505	-0.63064
8	C	-1.75746	-1.52772	-0.31919
9	O	-0.45817	-1.31845	-0.5531
10	C	0.488248	-2.34412	-0.16145
11	C	1.794882	-2.03085	-0.89859
12	C	3.520073	-0.67523	0.191076
13	H	4.003696	-1.56243	0.585396
14	Cl	4.337111	0.796987	0.65398
15	C	0.632844	-2.37585	1.35137
16	H	0.099396	-3.30316	-0.52054
17	H	2.493899	-2.84073	-0.66351
18	H	1.598666	-2.0723	-1.97676
19	H	2.535004	1.262825	-1.43381
20	H	1.174616	0.281516	-1.97716
21	H	0.548816	0.500007	0.690209
22	H	1.384391	2.024412	0.47684
23	C	-0.19646	3.272128	-1.18931
24	H	-0.69871	1.218477	-1.54896
25	O	-1.54196	2.662742	1.25019
26	C	-3.12472	0.523922	1.664124
27	H	-2.1758	0.147484	-1.56013
28	C	-4.04256	-0.58879	-0.83284
29	O	-2.21832	-2.56148	0.107669
30	H	-1.07455	3.667643	-1.70799
31	H	0.026091	3.926468	-0.34323
32	H	0.649236	3.293533	-1.88362
33	H	-4.13577	0.933657	1.570991
34	H	-2.61588	1.035226	2.478865
35	H	-3.19149	-0.54443	1.88729
36	H	-4.46492	-1.1148	0.024674
37	H	-4.60175	0.333942	-1.00492
38	H	-4.15423	-1.23522	-1.7065
39	H	-0.32307	-2.60889	1.824335
40	H	1.002302	-1.41932	1.730071
41	H	1.351855	-3.15197	1.630268

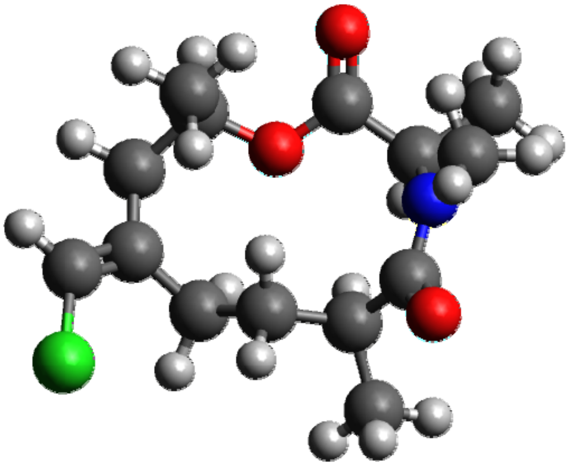

Table S37. Cartesian Coordinates For The Optimized Conformer 19 Of Compound 3*R*,6*S*,11*R*-**10t**

#	Atomic	Coordinates (Angstrom)		
		X	Y	Z
1	C	-2.35754	-0.33893	-0.27957
2	C	-1.47942	0.654824	0.441082
3	C	-0.60084	1.482974	-0.51055
4	C	0.612811	2.159978	0.191992
5	C	1.875189	1.669431	-0.51972
6	N	2.477076	0.501953	-0.12991
7	C	2.167554	-0.26802	1.061765
8	C	1.288165	-1.49152	0.774476
9	O	0.511588	-1.31066	-0.29442
10	C	-0.6084	-2.21327	-0.47942
11	C	-1.70483	-1.39212	-1.15787
12	C	-3.68944	-0.35212	-0.20006
13	H	-4.31247	-1.07746	-0.71165
14	Cl	-4.63901	0.786832	0.723252
15	C	-0.15733	-3.39577	-1.31838
16	H	-0.93204	-2.54798	0.512516
17	H	-1.26743	-0.92503	-2.05013
18	H	-2.47142	-2.08832	-1.51348
19	H	-2.09204	1.322044	1.05297
20	H	-0.84246	0.098353	1.139742
21	H	-0.23717	0.846909	-1.32122
22	H	-1.21833	2.257357	-0.97947
23	C	0.532072	3.684151	0.121732
24	H	0.613586	1.875393	1.247845
25	O	2.286297	2.280586	-1.50427
26	C	3.485698	-0.07433	-1.01344
27	H	1.546091	0.345913	1.720869
28	C	3.415982	-0.66467	1.849282
29	O	1.240369	-2.46038	1.498287
30	H	1.37752	4.152843	0.633131
31	H	0.539241	4.014634	-0.91901
32	H	-0.39286	4.024321	0.596873
33	H	4.500494	0.158863	-0.67533
34	H	3.347107	0.330338	-2.01376
35	H	3.361071	-1.16159	-1.0402
36	H	4.040673	-1.36534	1.29033
37	H	4.001261	0.229019	2.077207
38	H	3.12108	-1.1489	2.780909
39	H	0.667351	-3.91535	-0.82531
40	H	0.172831	-3.05516	-2.30449
41	H	-0.98257	-4.10077	-1.44896

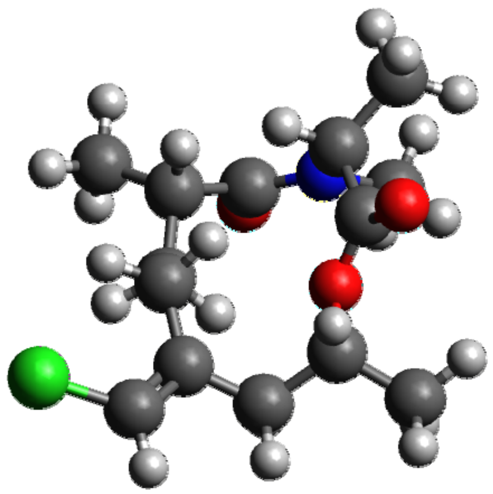

Table S38. Cartesian Coordinates For The Optimized Conformer 15 Of Compound 3*R*,6*S*,11*R*-**10t**

#	Atomic	Coordinates (Angstrom)		
		X	Y	Z
1	C	-2.3574	-0.33894	-0.27948
2	C	-1.47928	0.654705	0.441322
3	C	-0.60127	1.483462	-0.51032
4	C	0.612781	2.160231	0.191772
5	C	1.874861	1.669006	-0.52004
6	N	2.477201	0.502062	-0.12939
7	C	2.167227	-0.26787	1.062192
8	C	1.288155	-1.49147	0.774415
9	O	0.51183	-1.31051	-0.29467
10	C	-0.60816	-2.21311	-0.47992
11	C	-1.70462	-1.39183	-1.15808
12	C	-3.6893	-0.35221	-0.19992
13	H	-4.31226	-1.07749	-0.71167
14	Cl	-4.63889	0.786436	0.723694
15	C	-0.15704	-3.39536	-1.31921
16	H	-0.93174	-2.54814	0.511927
17	H	-1.26731	-0.92444	-2.05023
18	H	-2.47118	-2.08798	-1.51391
19	H	-2.09183	1.321529	1.053698
20	H	-0.84196	0.098119	1.139546
21	H	-0.23805	0.847943	-1.32163
22	H	-1.21915	2.258048	-0.9784
23	C	0.532663	3.684416	0.120807
24	H	0.613641	1.876154	1.247773
25	O	2.285455	2.279435	-1.50526
26	C	3.486003	-0.07457	-1.01252
27	H	1.545275	0.345965	1.720934
28	C	3.415357	-0.66429	1.850273
29	O	1.240213	-2.46042	1.498095
30	H	1.378444	4.153004	0.631755
31	H	0.539679	4.014397	-0.9201
32	H	-0.392	4.025191	0.596045
33	H	4.500717	0.158477	-0.67406
34	H	3.347827	0.329972	-2.01293
35	H	3.361191	-1.16182	-1.03915
36	H	4.040244	-1.36508	1.291677
37	H	4.000513	0.229483	2.078162
38	H	3.120173	-1.14834	2.781898
39	H	0.667666	-3.91502	-0.82628
40	H	0.173088	-3.05447	-2.30524
41	H	-0.98226	-4.10036	-1.44998

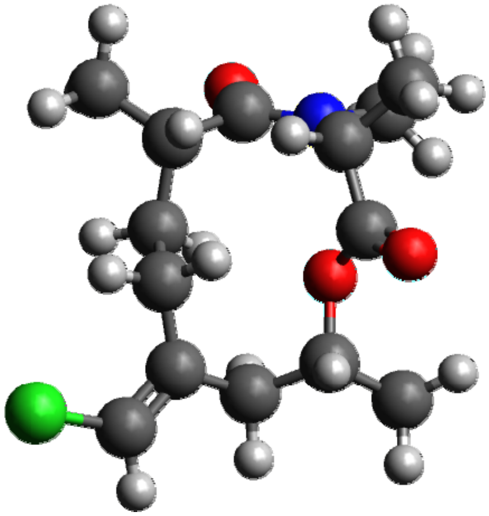

Table S39. Cartesian Coordinates For The Optimized Conformer 11 Of Compound 3*R*,6*S*,11*R*-**10t**

#	Atomic	Coordinates (Angstrom)		
		X	Y	Z
1	C	-1.23344	-1.49016	-0.39179
2	C	-2.23786	-0.97178	-1.39093
3	C	-1.90909	0.440882	-1.92512
4	C	-1.49806	1.441972	-0.83576
5	C	-0.00966	1.281023	-0.52377
6	N	0.457377	1.502642	0.73293
7	C	1.88524	1.263892	0.963633
8	C	2.313468	-0.07907	0.356834
9	O	1.443764	-1.03088	0.732238
10	C	1.205482	-2.26759	0.015458
11	C	0.064427	-2.0225	-0.96955
12	C	-1.44978	-1.48885	0.925054
13	H	-0.73252	-1.81186	1.668317
14	Cl	-2.93751	-0.94161	1.676169
15	C	2.420676	-2.89264	-0.65446
16	H	0.86977	-2.94175	0.810723
17	H	0.414726	-1.32399	-1.73993
18	H	-0.15622	-2.97049	-1.48021
19	H	-2.28507	-1.6643	-2.24008
20	H	-3.22969	-0.95619	-0.93234
21	H	-1.10936	0.397474	-2.67018
22	H	-2.80241	0.819692	-2.43347
23	C	-1.71989	2.891329	-1.29457
24	H	-2.09991	1.249634	0.057076
25	O	0.783463	0.997615	-1.42628
26	C	-0.36338	1.761254	1.905087
27	H	2.003306	1.142391	2.047986
28	C	2.760112	2.411104	0.477086
29	O	3.330672	-0.25237	-0.26576
30	H	-1.39449	3.609266	-0.5347
31	H	-1.15225	3.084866	-2.21016
32	H	-2.77932	3.065873	-1.50036
33	H	-0.55765	0.836221	2.461961
34	H	-1.31313	2.21463	1.626579
35	H	0.161802	2.465459	2.556046
36	H	2.652386	2.52707	-0.60263
37	H	2.459344	3.336456	0.975146
38	H	3.808943	2.209524	0.704658
39	H	3.259352	-2.95982	0.040722
40	H	2.741577	-2.3252	-1.52865
41	H	2.144194	-3.90432	-0.96684

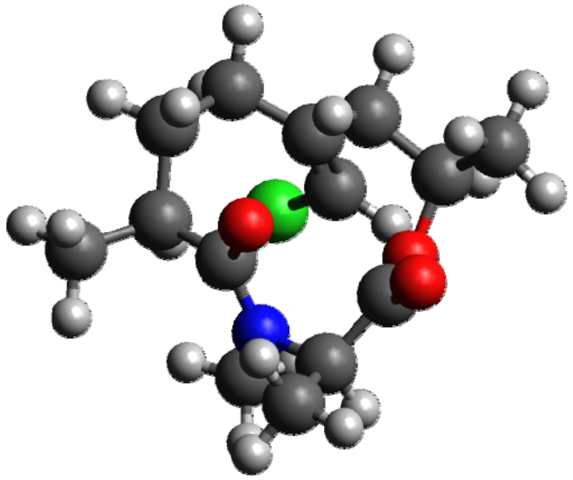

Table S40. Cartesian Coordinates For The Optimized Conformer 7 Of Compound 3*R*,6*S*,11*R*-**10t**

#	Atomic	Coordinates (Angstrom)		
		X	Y	Z
1	C	2.106273	0.582667	-0.08403
2	C	1.555934	-0.604	0.669189
3	C	1.239	-1.81658	-0.24599
4	C	-0.15021	-2.40549	0.038315
5	C	-1.1606	-1.31363	-0.30263
6	N	-2.12464	-0.96403	0.588634
7	C	-2.87148	0.266403	0.311947
8	C	-1.90718	1.392204	-0.08517
9	O	-0.88147	1.439379	0.777315
10	C	0.286147	2.263528	0.519914
11	C	1.13993	1.631316	-0.595
12	C	3.405198	0.740643	-0.34013
13	H	3.822145	1.577878	-0.8883
14	Cl	4.628269	-0.40844	0.155515
15	C	-0.04602	3.729804	0.276761
16	H	0.821948	2.18471	1.471828
17	H	0.464333	1.183856	-1.33501
18	H	1.699097	2.42486	-1.10159
19	H	2.267189	-0.90595	1.443231
20	H	0.64288	-0.28928	1.186908
21	H	1.27076	-1.51657	-1.29808
22	H	1.99459	-2.59655	-0.10726
23	C	-0.42848	-3.65168	-0.8069
24	H	-0.20051	-2.66258	1.100948
25	O	-1.05881	-0.70584	-1.37101
26	C	-2.37432	-1.63854	1.855047
27	H	-3.30669	0.581901	1.268767
28	C	-3.97541	0.075237	-0.7188
29	O	-2.09123	2.161265	-0.99625
30	H	-1.41527	-4.07326	-0.5908
31	H	-0.39018	-3.39644	-1.86993
32	H	0.323056	-4.42018	-0.60639
33	H	-1.77847	-1.20794	2.669077
34	H	-2.163	-2.70422	1.773629
35	H	-3.43347	-1.53446	2.101184
36	H	-3.54215	-0.22122	-1.67514
37	H	-4.6653	-0.6997	-0.37468
38	H	-4.52867	1.006535	-0.85707
39	H	-0.79066	4.075643	0.998929
40	H	-0.42715	3.899202	-0.72982
41	H	0.866749	4.315679	0.41984

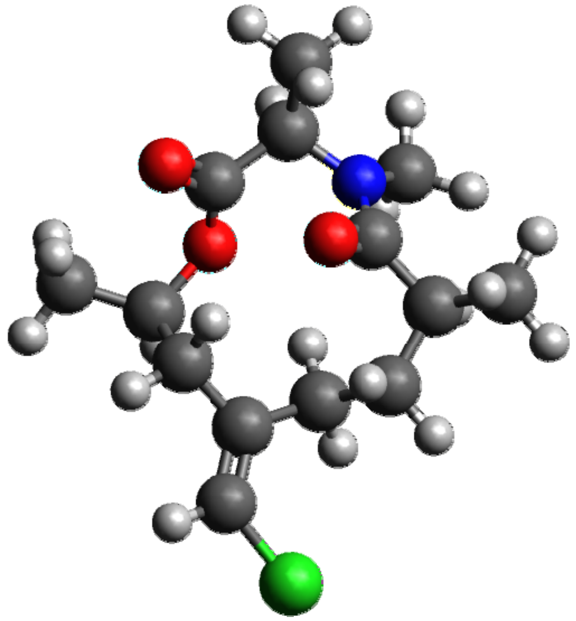

Table S41. Boltzmann Distribution of Conformers of (3*R*,6*R*,11*S*)-**10t**

Conformer	Energy (kcal/mol)	Relative Energy (kcal/mol)	Boltzmann Factor	Equil Mole Fraction	Imaginary Frequency
1	-807442	0	1	0.818	0
3	-807440	1.50	0.080	0.065	0
13	-807440	1.83	0.045	0.037	0
17	-807439	2.09	0.029	0.024	0
10	-807439	2.51	0.015	0.012	0
19	-807439	2.62	0.012	0.010	0
7	-807439	2.68	0.011	0.009	0
14	-807439	2.90	0.008	0.006	0
5	-807439	2.91	0.007	0.006	0
9	-807438	3.14	0.005	0.004	0
16	-807438	3.26	0.004	0.003	0
2	-807438	3.57	0.002	0.002	0
6	-807438	3.61	0.002	0.002	0
8	-807437	4.15	0.001	0.001	0
15	-807437	4.25	0.001	0.001	0
4	-807437	4.46	0.001	0.000	0
11	-807437	4.76	0.000	0.000	0
18	-807437	4.85	0.000	0.000	0
12	-807437	4.92	0.000	0.000	0

XYZ coordinates listed in following tables for any conformer with an equilibrium mole fraction greater than or equal to 0.001

Table S42. Cartesian Coordinates For The Optimized Conformer 1 Of Compound 3*R*,6*R*,11*S*-**10t**

#	Atomic	Coordinates (Angstrom)		
		X	Y	Z
1	C	2.126493	-0.383	-0.30626
2	C	1.456603	0.705973	0.49236
3	C	0.961503	1.87171	-0.40056
4	C	-0.4381	2.361077	0.003234
5	C	-1.39271	1.200326	-0.26602
6	N	-2.15782	0.693727	0.736594
7	C	-2.84018	-0.57634	0.479997
8	C	-1.88435	-1.58362	-0.16644
9	O	-0.68275	-1.54471	0.427206
10	C	0.407772	-2.29552	-0.15626
11	C	1.242513	-1.36223	-1.05006
12	C	3.448565	-0.50817	-0.42767
13	H	3.931978	-1.27844	-1.01838
14	Cl	4.603439	0.563119	0.334163
15	C	1.187386	-2.90984	0.990361
16	H	-0.02788	-3.07509	-0.78648
17	H	1.861021	-1.99516	-1.69595
18	H	0.546191	-0.81119	-1.69568
19	H	0.604542	0.261655	1.018085
20	H	2.139202	1.0874	1.255775
21	H	1.666661	2.707543	-0.34396
22	H	0.915738	1.559186	-1.4492
23	C	-0.86919	3.585838	-0.80723
24	H	-0.41664	2.613028	1.067481
25	O	-1.41283	0.677927	-1.38335
26	C	-2.27057	1.264602	2.072847
27	H	-3.07667	-1.00223	1.463829
28	C	-4.12036	-0.41223	-0.32914
29	O	-2.20138	-2.36459	-1.03078
30	H	-0.15951	4.404612	-0.66069
31	H	-0.89955	3.335637	-1.87148
32	H	-1.86225	3.935715	-0.50839
33	H	-3.2497	1.002643	2.479508
34	H	-2.21538	2.352034	2.036383
35	H	-1.49515	0.880816	2.746925
36	H	-3.88574	-0.00237	-1.31284
37	H	-4.61288	-1.37804	-0.45771
38	H	-4.79854	0.265903	0.195308
39	H	0.561609	-3.61265	1.545184
40	H	1.544735	-2.1356	1.675649
41	H	2.057954	-3.44513	0.600187

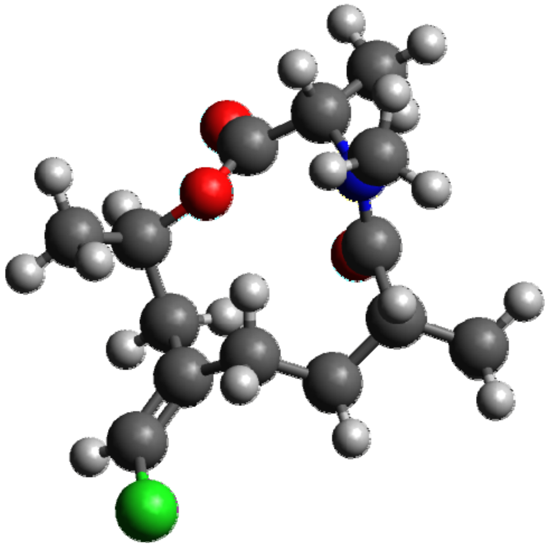

Table S43. Cartesian Coordinates For The Optimized Conformer 3 Of Compound 3*R*,6*R*,11*S*-**10t**

#	Atomic	Coordinates (Angstrom)		
		X	Y	Z
1	C	-2.21696	0.672293	0.173895
2	C	-1.50686	-0.56829	-0.29605
3	C	-0.90036	-1.3854	0.860407
4	C	0.181546	-2.39183	0.393998
5	C	1.323161	-1.55793	-0.17388
6	N	2.220892	-0.97205	0.676819
7	C	2.95884	0.172424	0.135183
8	C	1.976849	1.185256	-0.46477
9	O	0.870815	1.263211	0.293032
10	C	-0.15654	2.218466	-0.06672
11	C	-1.39465	1.814636	0.738897
12	C	-3.54091	0.833732	0.139284
13	H	-4.04519	1.73871	0.459773
14	Cl	-4.6678	-0.38385	-0.41126
15	C	0.304522	3.622814	0.293335
16	H	-0.33047	2.133541	-1.14602
17	H	-1.04438	1.559745	1.748898
18	H	-2.03594	2.695244	0.84682
19	H	-2.19739	-1.19391	-0.8659
20	H	-0.71108	-0.26873	-0.98712
21	H	-0.46153	-0.70617	1.600061
22	H	-1.69834	-1.93878	1.369376
23	C	-0.34682	-3.37376	-0.64845
24	H	0.51325	-2.95956	1.267795
25	O	1.370145	-1.30614	-1.37809
26	C	2.169193	-1.05547	2.129775
27	H	3.393959	0.688896	1.00079
28	C	4.081124	-0.19245	-0.82694
29	O	2.190094	1.872032	-1.43414
30	H	-1.22764	-3.89179	-0.25725
31	H	-0.6194	-2.85671	-1.57026
32	H	0.410827	-4.12116	-0.89856
33	H	3.189395	-1.03647	2.521685
34	H	1.707553	-1.98566	2.453338
35	H	1.611347	-0.21021	2.552835
36	H	3.678427	-0.67051	-1.71921
37	H	4.622858	0.709405	-1.12021
38	H	4.77383	-0.87485	-0.32845
39	H	1.236832	3.867582	-0.21787
40	H	0.453531	3.701878	1.375102
41	H	-0.45533	4.349565	-0.00623

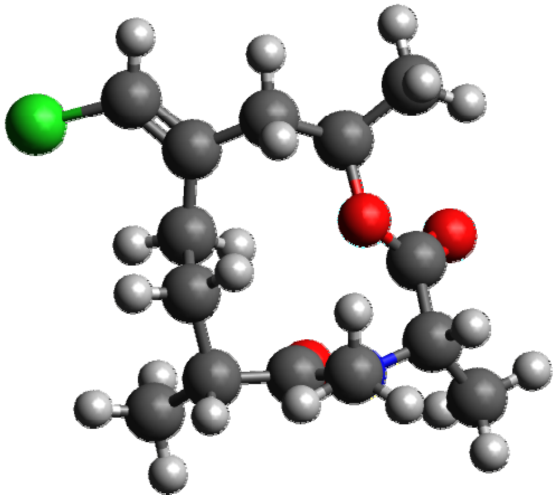

Table S44. Cartesian Coordinates For The Optimized Conformer 13 Of Compound 3*R*,6*R*,11*S*-**10t**

#	Atomic	Coordinates (Angstrom)		
		X	Y	Z
1	C	2.061674	-0.60478	-0.66563
2	C	1.441962	0.761948	-0.83536
3	C	0.956786	1.414055	0.478626
4	C	-0.25758	2.354041	0.262977
5	C	-1.41717	1.453908	-0.14047
6	N	-2.14382	0.815158	0.828807
7	C	-2.88579	-0.36834	0.390399
8	C	-1.94278	-1.36611	-0.29116
9	O	-0.71995	-1.30951	0.261696
10	C	0.249053	-2.33417	-0.05471
11	C	1.255014	-1.81601	-1.09003
12	C	3.2939	-0.79637	-0.19401
13	H	3.767595	-1.76684	-0.09228
14	Cl	4.333271	0.512404	0.318453
15	C	0.882048	-2.7355	1.265423
16	H	-0.28872	-3.17753	-0.49759
17	H	1.925924	-2.65262	-1.31619
18	H	0.709096	-1.57911	-2.01075
19	H	2.170151	1.426136	-1.31511
20	H	0.597647	0.663549	-1.52258
21	H	0.684832	0.632627	1.197127
22	H	1.770541	1.991511	0.929305
23	C	0.010058	3.419026	-0.79757
24	H	-0.46825	2.85711	1.209933
25	O	-1.62874	1.205029	-1.32779
26	C	-1.8471	0.870762	2.254807
27	H	-3.19604	-0.88599	1.307906
28	C	-4.13306	-0.07802	-0.43488
29	O	-2.27858	-2.16378	-1.13322
30	H	0.898766	3.996879	-0.52627
31	H	0.168576	2.966653	-1.77832
32	H	-0.83605	4.106441	-0.88146
33	H	-2.7791	0.750796	2.812499
34	H	-1.41912	1.831515	2.530894
35	H	-1.15399	0.070238	2.544338
36	H	-3.86809	0.38778	-1.38285
37	H	-4.66619	-1.0107	-0.63095
38	H	-4.78734	0.591066	0.129158
39	H	0.124955	-3.14432	1.938894
40	H	1.354586	-1.87385	1.745535
41	H	1.647403	-3.49889	1.097326

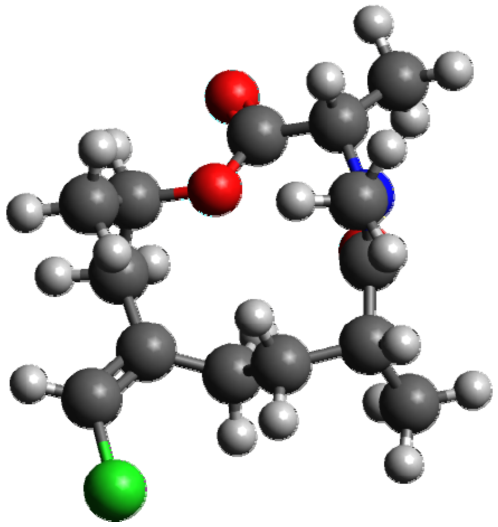

Table S45. Cartesian Coordinates For The Optimized Conformer 17 Of Compound 3*R*,6*R*,11*S*-**10t**

#	Atomic	Coordinates (Angstrom)		
		X	Y	Z
1	C	-1.40615	1.515945	-0.1933
2	C	-1.48486	0.797414	-1.52436
3	C	-1.87468	-0.69397	-1.56134
4	C	-1.2555	-1.58397	-0.47814
5	C	0.252751	-1.40902	-0.44265
6	N	0.901775	-1.5647	0.748202
7	C	2.289634	-1.10062	0.798498
8	C	2.3946	0.272493	0.130116
9	O	1.346691	1.038524	0.471434
10	C	1.100125	2.220669	-0.31364
11	C	-0.35776	2.607701	-0.06361
12	C	-2.25289	1.402005	0.832503
13	H	-2.14914	1.973621	1.747928
14	Cl	-3.6912	0.407991	0.860016
15	C	2.037595	3.350871	0.084352
16	H	1.266079	1.945807	-1.36212
17	H	-0.4282	3.061314	0.931566
18	H	-0.6009	3.398648	-0.7875
19	H	-2.18949	1.354091	-2.15831
20	H	-0.5079	0.892121	-2.00525
21	H	-2.95964	-0.81149	-1.49555
22	H	-1.56922	-1.07555	-2.5417
23	C	-1.5963	-3.05928	-0.73234
24	H	-1.67172	-1.28741	0.487185
25	O	0.882337	-1.12444	-1.46348
26	C	0.205642	-1.74331	2.016475
27	H	2.524176	-0.93224	1.857003
28	C	3.283162	-2.09339	0.210013
29	O	3.30783	0.633996	-0.57125
30	H	-2.68157	-3.19141	-0.77043
31	H	-1.17509	-3.38377	-1.68899
32	H	-1.1999	-3.71175	0.051748
33	H	0.911104	-2.14612	2.745425
34	H	-0.60583	-2.46369	1.915365
35	H	-0.19565	-0.79283	2.392521
36	H	3.062347	-2.25893	-0.84535
37	H	4.300049	-1.7057	0.300351
38	H	3.212911	-3.04054	0.750156
39	H	3.073116	3.089856	-0.13539
40	H	1.937336	3.559235	1.154114
41	H	1.777925	4.257915	-0.46958

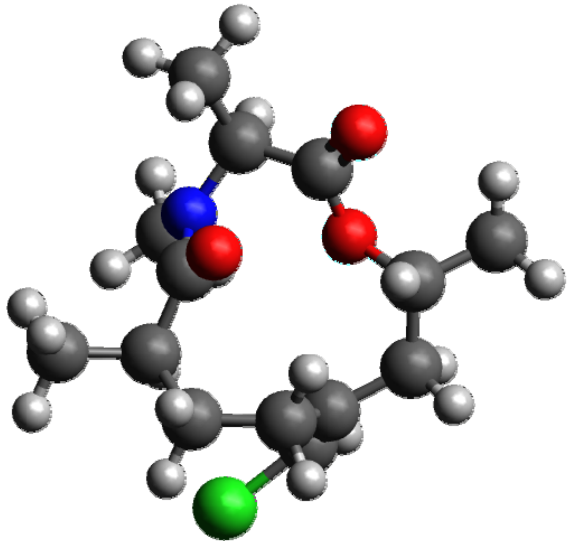

Table S46. Cartesian Coordinates For The Optimized Conformer 10 Of Compound 3*R*,6*R*,11*S*-**10t**

#	Atomic	Coordinates (Angstrom)		
		X	Y	Z
1	C	-2.08361	0.837824	0.467184
2	C	-1.54424	-0.4483	1.058874
3	C	-1.43675	-1.65923	0.099607
4	C	-0.08939	-2.38416	0.235882
5	C	0.978238	-1.41042	-0.2486
6	N	2.000334	-1.0521	0.573532
7	C	2.837675	0.06653	0.141708
8	C	1.973828	1.215685	-0.3822
9	O	0.90214	1.40914	0.399623
10	C	-0.0614	2.399978	-0.02111
11	C	-1.34891	2.130858	0.759972
12	C	-3.23017	0.929194	-0.20803
13	H	-3.62531	1.859229	-0.60101
14	Cl	-4.29653	-0.4203	-0.51124
15	C	0.465932	3.797816	0.270422
16	H	-0.22653	2.260388	-1.0959
17	H	-1.10353	2.161743	1.830891
18	H	-2.01581	2.977067	0.564172
19	H	-0.55609	-0.2249	1.474323
20	H	-2.17887	-0.71727	1.914338
21	H	-2.24031	-2.37508	0.294435
22	H	-1.53926	-1.33419	-0.94001
23	C	-0.04551	-3.65872	-0.61098
24	H	0.058173	-2.63867	1.289615
25	O	0.873758	-0.89848	-1.36497
26	C	2.28012	-1.64606	1.874244
27	H	3.318741	0.460888	1.046556
28	C	3.900485	-0.34408	-0.86959
29	O	2.263977	1.908627	-1.32769
30	H	-0.83945	-4.34452	-0.30287
31	H	-0.18924	-3.40873	-1.66589
32	H	0.91354	-4.17618	-0.50833
33	H	3.351478	-1.56245	2.069601
34	H	2.031745	-2.70687	1.878658
35	H	1.735503	-1.13993	2.680622
36	H	3.421142	-0.72043	-1.77491
37	H	4.525893	0.5115	-1.13177
38	H	4.530586	-1.12684	-0.43921
39	H	1.403476	3.974927	-0.25753
40	H	0.628645	3.919176	1.346233
41	H	-0.26266	4.545143	-0.05581

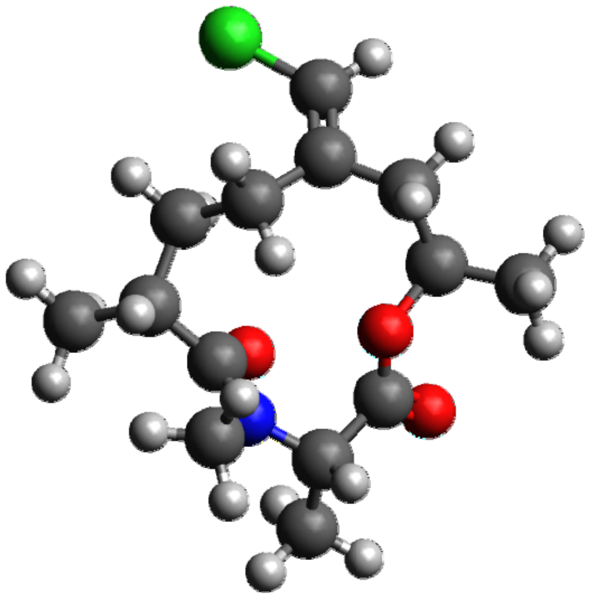

Table S47. Cartesian Coordinates For The Optimized Conformer 19 Of Compound 3*R*,6*R*,11*S*-**10t**

#	Atomic	Coordinates (Angstrom)		
		X	Y	Z
1	C	-2.11262	-0.5353	0.718226
2	C	-1.32567	0.755564	0.626193
3	C	-0.74403	1.014706	-0.77961
4	C	0.394638	2.063862	-0.80941
5	C	1.390409	1.843559	0.336494
6	N	2.231385	0.7645	0.31787
7	C	2.355138	-0.21597	-0.74819
8	C	1.728887	-1.55692	-0.33023
9	O	0.599494	-1.34249	0.339963
10	C	-0.36098	-2.40105	0.552665
11	C	-1.46823	-1.73969	1.381705
12	C	-3.36183	-0.66027	0.268368
13	H	-3.94639	-1.57141	0.335242
14	Cl	-4.24638	0.647246	-0.48044
15	C	-0.86217	-2.95746	-0.77104
16	H	0.123317	-3.18845	1.141451
17	H	-2.22359	-2.50288	1.596089
18	H	-1.03843	-1.42376	2.339896
19	H	-1.97219	1.592983	0.912142
20	H	-0.51508	0.710531	1.359326
21	H	-0.3933	0.070594	-1.19898
22	H	-1.54268	1.368171	-1.4411
23	C	-0.16349	3.484958	-0.72903
24	H	0.922821	1.960754	-1.76611
25	O	1.407593	2.614729	1.294305
26	C	3.028092	0.512351	1.514961
27	H	1.752093	0.120161	-1.59777
28	C	3.794407	-0.39671	-1.21948
29	O	2.172012	-2.6454	-0.61504
30	H	-0.82783	3.663999	-1.579
31	H	-0.72626	3.630663	0.196006
32	H	0.637238	4.227742	-0.74594
33	H	3.198831	-0.5634	1.613965
34	H	2.484921	0.87338	2.387212
35	H	3.996348	1.021526	1.469883
36	H	4.429523	-0.7943	-0.42423
37	H	3.826043	-1.09942	-2.05372
38	H	4.193708	0.56667	-1.54448
39	H	-0.05869	-3.44687	-1.32259
40	H	-1.29394	-2.1609	-1.384
41	H	-1.64587	-3.69507	-0.57449

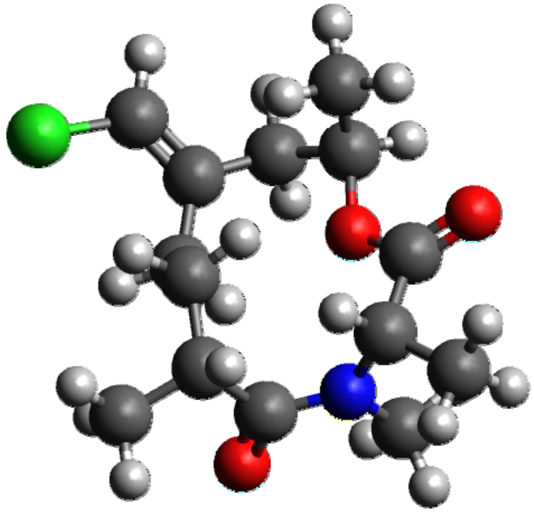

Table S48. Cartesian Coordinates For The Optimized Conformer 7 Of Compound 3*R*,6*R*,11*S*-**10t**

#	Atomic	Coordinates (Angstrom)		
		X	Y	Z
1	C	-1.47246	1.153457	-0.56548
2	C	-2.42051	0.310183	-1.38307
3	C	-1.83483	-1.07061	-1.76035
4	C	-1.16699	-1.81642	-0.59479
5	C	0.274954	-1.3364	-0.42097
6	N	0.842344	-1.3092	0.814665
7	C	2.199807	-0.76456	0.915876
8	C	2.316947	0.533896	0.112717
9	O	1.250524	1.313583	0.346664
10	C	0.883476	2.320119	-0.61873
11	C	-0.3586	1.810912	-1.36289
12	C	-1.58154	1.28116	0.758127
13	H	-0.88401	1.812638	1.388783
14	Cl	-2.87446	0.569714	1.710209
15	C	0.723545	3.657381	0.088153
16	H	1.703534	2.40202	-1.33807
17	H	-0.79203	2.648087	-1.92799
18	H	-0.02549	1.076216	-2.10399
19	H	-3.35175	0.163956	-0.83071
20	H	-2.67232	0.845375	-2.30666
21	H	-2.65713	-1.68459	-2.14367
22	H	-1.10416	-0.97417	-2.56902
23	C	-1.11965	-3.3298	-0.85799
24	H	-1.74374	-1.62616	0.314909
25	O	0.947348	-1.0202	-1.40657
26	C	0.151337	-1.58468	2.064756
27	H	2.335282	-0.47158	1.965154
28	C	3.270758	-1.77225	0.521071
29	O	3.261882	0.844406	-0.56985
30	H	-2.13262	-3.73041	-0.95031
31	H	-0.58134	-3.53034	-1.78971
32	H	-0.61039	-3.86493	-0.04993
33	H	0.847143	-2.07349	2.751965
34	H	-0.68649	-2.26198	1.907702
35	H	-0.21711	-0.66007	2.526021
36	H	3.140648	-2.05902	-0.52396
37	H	4.264433	-1.33654	0.644156
38	H	3.189734	-2.65923	1.15449
39	H	1.647171	3.913412	0.61313
40	H	-0.09937	3.658916	0.805743
41	H	0.523853	4.434564	-0.65479

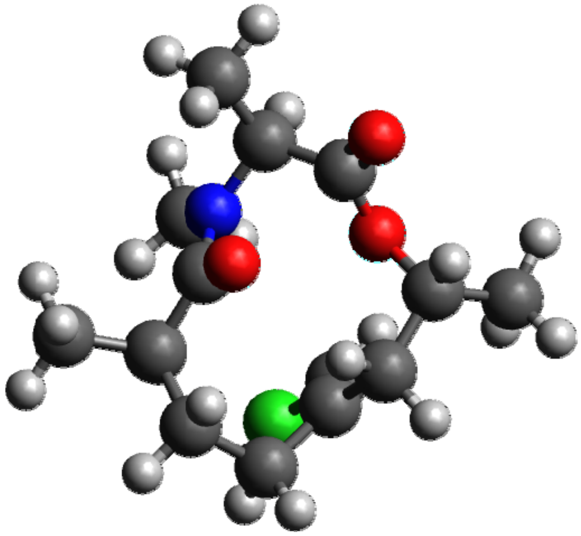

Table S49. Cartesian Coordinates For The Optimized Conformer 14 Of Compound 3*R*,6*R*,11*S*-**10t**

#	Atomic	Coordinates (Angstrom)		
		X	Y	Z
1	C	-2.46213	0.46858	0.342011
2	C	-1.55312	-0.54414	0.999538
3	C	-0.54604	-1.16795	0.015885
4	C	0.611046	-1.90149	0.736175
5	C	1.866757	-1.84405	-0.13591
6	N	2.560983	-0.65944	-0.21241
7	C	2.240538	0.533033	0.570494
8	C	1.292828	1.445702	-0.21098
9	O	0.214359	1.757702	0.5162
10	C	-0.88159	2.430085	-0.15224
11	C	-2.16329	1.942581	0.523813
12	C	-3.52415	0.126338	-0.38829
13	H	-4.18768	0.836371	-0.86954
14	Cl	-4.00792	-1.52979	-0.65628
15	C	-0.68823	3.931819	-0.03826
16	H	-0.86652	2.119973	-1.20287
17	H	-2.09016	2.167522	1.596632
18	H	-2.98956	2.53924	0.12258
19	H	-1.00394	-0.04573	1.804639
20	H	-2.15609	-1.33851	1.454293
21	H	-1.06167	-1.8811	-0.63743
22	H	-0.16021	-0.39153	-0.652
23	C	0.241325	-3.34853	1.05214
24	H	0.807625	-1.38981	1.6847
25	O	2.21563	-2.82314	-0.78977
26	C	3.632552	-0.55034	-1.20166
27	H	1.720766	0.231249	1.479018
28	C	3.488536	1.317876	0.981182
29	O	1.481276	1.815305	-1.34758
30	H	-0.67892	-3.37686	1.643269
31	H	0.08587	-3.90993	0.128379
32	H	1.031909	-3.84607	1.619617
33	H	3.522628	0.383994	-1.75529
34	H	3.548972	-1.39135	-1.8859
35	H	4.616627	-0.58222	-0.72328
36	H	4.00006	1.742238	0.114903
37	H	3.196421	2.138033	1.642212
38	H	4.177609	0.664278	1.52137
39	H	0.258948	4.226333	-0.49603
40	H	-0.68684	4.235049	1.013074
41	H	-1.49839	4.455191	-0.55292

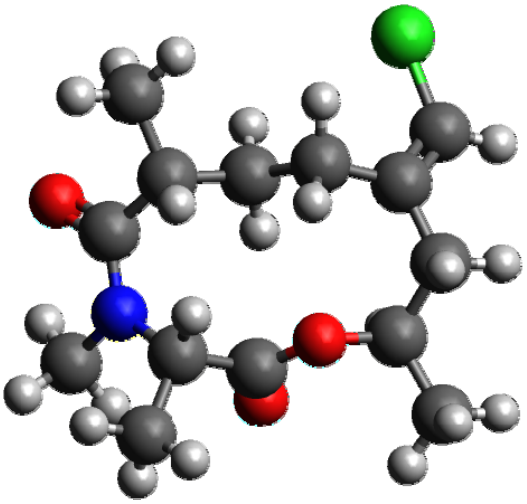

Table S50. Cartesian Coordinates For The Optimized Conformer 5 Of Compound 3*R*,6*R*,11*S*-**10t**

#	Atomic	Coordinates (Angstrom)		
		X	Y	Z
1	C	-2.46216	0.468234	0.34228
2	C	-1.55308	-0.54448	0.999804
3	C	-0.54594	-1.16824	0.016229
4	C	0.611388	-1.90138	0.73648
5	C	1.866917	-1.84389	-0.13586
6	N	2.560853	-0.65909	-0.21271
7	C	2.240404	0.533362	0.570188
8	C	1.292425	1.445914	-0.2111
9	O	0.214016	1.757627	0.516319
10	C	-0.88217	2.429937	-0.1519
11	C	-2.16358	1.942251	0.524498
12	C	-3.52395	0.12603	-0.38836
13	H	-4.18756	0.836021	-0.86955
14	Cl	-4.00724	-1.53018	-0.65706
15	C	-0.68879	3.931667	-0.03807
16	H	-0.86738	2.119674	-1.2025
17	H	-2.09029	2.166937	1.597347
18	H	-2.99003	2.538856	0.123551
19	H	-1.00393	-0.04604	1.80491
20	H	-2.1561	-1.33879	1.454585
21	H	-1.06143	-1.88155	-0.63701
22	H	-0.16038	-0.39176	-0.65173
23	C	0.242082	-3.34844	1.052848
24	H	0.80814	-1.38951	1.684853
25	O	2.215898	-2.82303	-0.78956
26	C	3.632393	-0.54992	-1.20193
27	H	1.720854	0.231624	1.47885
28	C	3.488372	1.318475	0.980523
29	O	1.480618	1.815695	-1.34769
30	H	-0.67804	-3.37686	1.64417
31	H	0.086557	-3.91012	0.129261
32	H	1.032901	-3.84567	1.620276
33	H	3.52207	0.384103	-1.75601
34	H	3.549323	-1.39129	-1.8858
35	H	4.61646	-0.58108	-0.72348
36	H	3.99959	1.742878	0.114086
37	H	3.196204	2.138611	1.641554
38	H	4.17771	0.665077	1.520616
39	H	0.258195	4.226106	-0.4963
40	H	-0.68687	4.234881	1.013265
41	H	-1.49916	4.45509	-0.55233

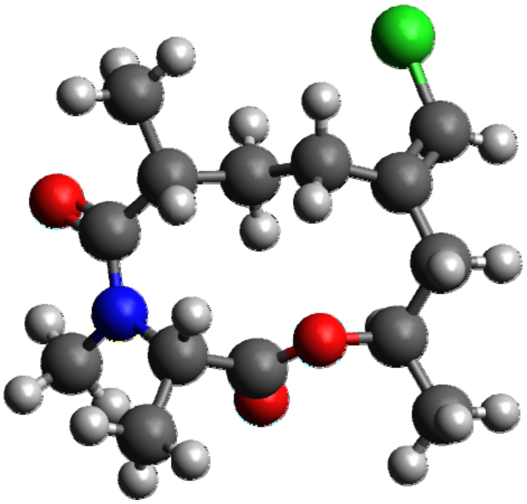

Table S51. Cartesian Coordinates For The Optimized Conformer 9 Of Compound 3*R*,6*R*,11*S*-**10t**

#	Atomic	Coordinates (Angstrom)		
		X	Y	Z
1	C	2.324569	-0.19574	-0.41364
2	C	1.471934	0.745269	0.403704
3	C	0.232797	1.25563	-0.34829
4	C	-0.85462	1.823877	0.594684
5	C	-2.20734	1.653619	-0.09772
6	N	-2.79386	0.409548	-0.09396
7	C	-2.29052	-0.72716	0.675839
8	C	-1.26418	-1.52541	-0.13162
9	O	-0.14495	-1.7198	0.571738
10	C	0.976064	-2.36957	-0.08342
11	C	1.667548	-1.40327	-1.05572
12	C	3.637003	-0.04232	-0.6001
13	H	4.244717	-0.73515	-1.17175
14	Cl	4.581248	1.28373	0.033747
15	C	1.881976	-2.86021	1.028068
16	H	0.575509	-3.20841	-0.66165
17	H	2.424902	-1.98504	-1.59236
18	H	0.936511	-1.08649	-1.80741
19	H	1.136084	0.200727	1.295681
20	H	2.074882	1.587309	0.751544
21	H	0.52322	2.035774	-1.06251
22	H	-0.19235	0.451791	-0.95558
23	C	-0.59913	3.288374	0.938939
24	H	-0.83479	1.24611	1.524238
25	O	-2.71325	2.591292	-0.70815
26	C	-3.92181	0.174479	-0.99392
27	H	-1.7875	-0.35678	1.568226
28	C	-3.4088	-1.66761	1.133466
29	O	-1.44231	-1.92344	-1.26106
30	H	0.391319	3.398892	1.390182
31	H	-0.64872	3.902651	0.037326
32	H	-1.34305	3.665411	1.645594
33	H	-3.73793	-0.72873	-1.58014
34	H	-4.00606	1.030398	-1.6593
35	H	-4.85805	0.067501	-0.43774
36	H	-3.89225	-2.16137	0.288207
37	H	-2.98439	-2.43709	1.783457
38	H	-4.15674	-1.10843	1.700529
39	H	1.350925	-3.56378	1.672969
40	H	2.240196	-2.02407	1.635496
41	H	2.75052	-3.3657	0.597136

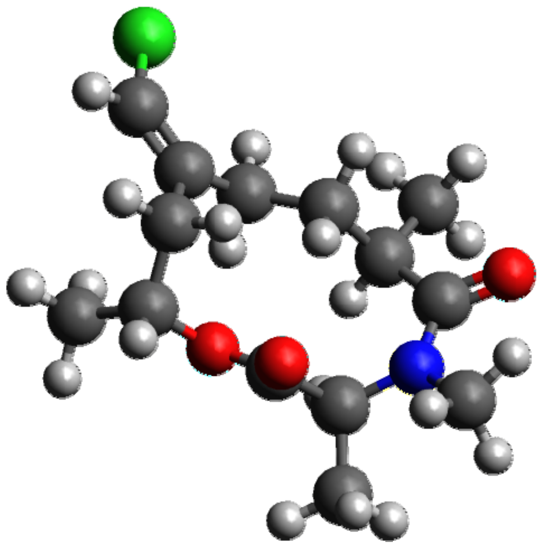

Table S52. Cartesian Coordinates For The Optimized Conformer 16 Of Compound 3*R*,6*R*,11*S*-**10t**

#	Atomic	Coordinates (Angstrom)		
		X	Y	Z
1	C	-2.15979	0.512629	-0.73566
2	C	-1.64162	-0.87554	-1.06524
3	C	-1.23753	-1.80436	0.098643
4	C	0.144715	-1.55879	0.714909
5	C	1.214678	-1.67933	-0.37272
6	N	2.324522	-0.87615	-0.3299
7	C	2.527327	0.179121	0.655074
8	C	1.822424	1.479074	0.243814
9	O	0.619068	1.215234	-0.26881
10	C	-0.31962	2.299591	-0.44135
11	C	-1.44682	1.730551	-1.30279
12	C	-3.30764	0.730037	-0.08976
13	H	-3.72138	1.716343	0.091375
14	Cl	-4.35336	-0.53811	0.501074
15	C	-0.78565	2.796822	0.917725
16	H	0.186925	3.102262	-0.98832
17	H	-2.16433	2.542367	-1.46227
18	H	-1.03491	1.467842	-2.28376
19	H	-2.44021	-1.38486	-1.62023
20	H	-0.7989	-0.787	-1.75741
21	H	-1.97795	-1.74762	0.901758
22	H	-1.25273	-2.83072	-0.28195
23	C	0.440291	-2.6037	1.801032
24	H	0.144025	-0.56502	1.163223
25	O	1.080599	-2.52485	-1.25585
26	C	3.220757	-0.86476	-1.48107
27	H	2.037767	-0.12489	1.586481
28	C	3.998348	0.424615	0.970563
29	O	2.266044	2.590569	0.417959
30	H	-0.3297	-2.56935	2.577033
31	H	0.447786	-3.60737	1.365118
32	H	1.411701	-2.43156	2.276524
33	H	3.269084	0.144473	-1.90777
34	H	2.828487	-1.55292	-2.22569
35	H	4.229682	-1.17738	-1.19965
36	H	4.533749	0.848124	0.118379
37	H	4.075047	1.134121	1.795631
38	H	4.473143	-0.51474	1.263442
39	H	0.046192	3.226047	1.478975
40	H	-1.22748	1.976753	1.491005
41	H	-1.54548	3.572496	0.783697

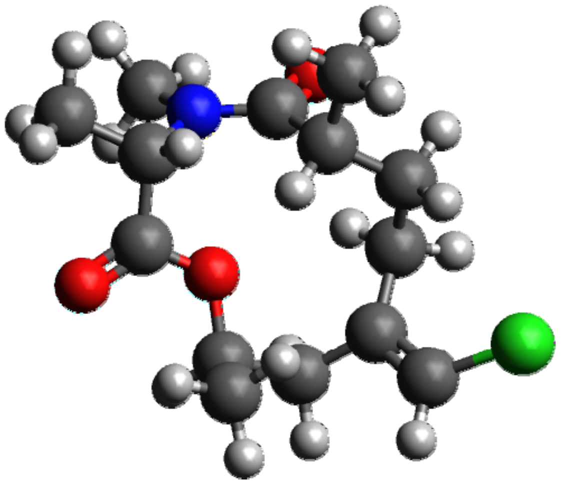

Table S53. Cartesian Coordinates For The Optimized Conformer 2 Of Compound 3*R*,6*R*,11*S*-**10t**

#	Atomic	Coordinates (Angstrom)		
		X	Y	Z
1	C	1.706668	1.061596	-0.53191
2	C	2.189042	-0.08069	-1.39063
3	C	1.133967	-1.10866	-1.84794
4	C	0.671536	-2.1646	-0.80928
5	C	-0.37364	-1.56424	0.121027
6	N	-1.67625	-1.49882	-0.30584
7	C	-2.56858	-0.6523	0.48662
8	C	-1.95929	0.739366	0.664135
9	O	-1.23455	1.074573	-0.41088
10	C	-0.52266	2.327406	-0.39655
11	C	0.762572	2.07061	-1.17518
12	C	2.132042	1.310731	0.705497
13	H	1.808551	2.166469	1.288402
14	Cl	3.276483	0.331959	1.586023
15	C	-1.39452	3.40302	-1.02111
16	H	-0.30692	2.576883	0.646524
17	H	0.486257	1.72667	-2.17932
18	H	1.281478	3.02782	-1.30566
19	H	2.601378	0.369241	-2.30456
20	H	3.018268	-0.59556	-0.8997
21	H	0.260289	-0.58249	-2.25348
22	H	1.575503	-1.66212	-2.6845
23	C	1.822716	-2.76278	-0.00562
24	H	0.209576	-2.97767	-1.37734
25	O	-0.0643	-1.10966	1.219352
26	C	-2.12191	-1.76203	-1.66773
27	H	-3.46281	-0.48796	-0.1288
28	C	-3.00044	-1.26614	1.812895
29	O	-2.16594	1.468075	1.605702
30	H	2.613568	-3.10639	-0.68038
31	H	2.237525	-2.03062	0.688384
32	H	1.475315	-3.61857	0.579593
33	H	-3.12349	-2.19906	-1.63655
34	H	-1.46627	-2.4686	-2.17102
35	H	-2.15828	-0.83358	-2.25228
36	H	-2.13608	-1.42077	2.458522
37	H	-3.70505	-0.60054	2.315624
38	H	-3.49002	-2.22436	1.623457
39	H	-2.32341	3.511584	-0.45598
40	H	-1.63677	3.143074	-2.05623
41	H	-0.87177	4.363428	-1.01495

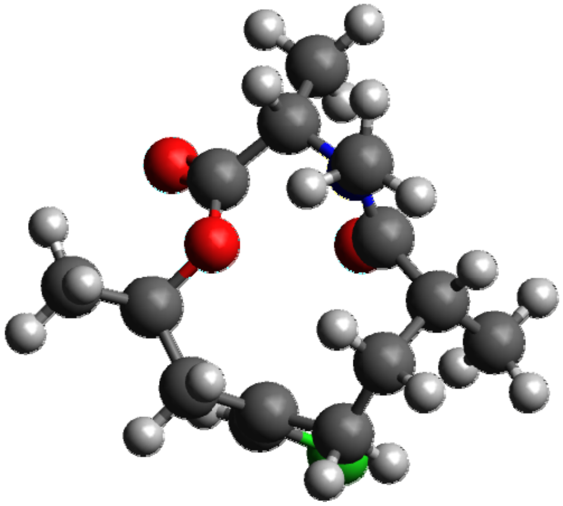

Table S54. Cartesian Coordinates For The Optimized Conformer 6 Of Compound 3*R*,6*R*,11*S*-**10t**

#	Atomic	Coordinates (Angstrom)		
		X	Y	Z
1	C	-2.32637	0.534279	-0.30846
2	C	-1.94728	-0.40983	-1.42642
3	C	-0.46479	-0.83696	-1.49673
4	C	0.046642	-1.63452	-0.27623
5	C	1.572701	-1.72534	-0.39651
6	N	2.37759	-0.87931	0.331015
7	C	1.841278	0.242324	1.107616
8	C	1.350928	1.318083	0.139123
9	O	0.082332	1.651509	0.38472
10	C	-0.61962	2.414789	-0.62305
11	C	-2.09004	2.021463	-0.47983
12	C	-2.92111	0.157804	0.825041
13	H	-3.20505	0.853114	1.607072
14	Cl	-3.35309	-1.48465	1.228694
15	C	-0.38521	3.900576	-0.42097
16	H	-0.22647	2.10725	-1.59799
17	H	-2.51358	2.557256	0.377145
18	H	-2.61669	2.375988	-1.3755
19	H	-2.57324	-1.30536	-1.36406
20	H	-2.19314	0.078386	-2.37794
21	H	-0.33835	-1.46949	-2.38344
22	H	0.167629	0.039869	-1.67099
23	C	-0.52492	-3.05511	-0.25683
24	H	-0.25844	-1.11114	0.634766
25	O	2.055875	-2.54638	-1.17265
26	C	3.804426	-0.86277	0.008731
27	H	0.9861	-0.10344	1.689353
28	C	2.856004	0.842579	2.079324
29	O	2.011845	1.764809	-0.77159
30	H	-1.61569	-3.03282	-0.26293
31	H	-0.17075	-3.60679	-1.1302
32	H	-0.20537	-3.59255	0.640858
33	H	4.054653	0.032742	-0.56919
34	H	4.024418	-1.74711	-0.58312
35	H	4.40027	-0.88592	0.922656
36	H	3.694142	1.305694	1.554739
37	H	2.357316	1.610903	2.675722
38	H	3.232503	0.072952	2.757118
39	H	0.671588	4.1428	-0.55065
40	H	-0.7033	4.198953	0.582325
41	H	-0.96544	4.469214	-1.1534

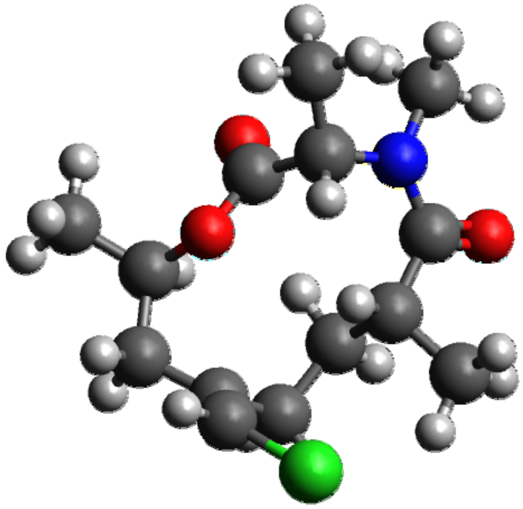

Table S55. Cartesian Coordinates For The Optimized Conformer 8 Of Compound 3*R*,6*R*,11*S*-**10t**

#	Atomic	Coordinates (Angstrom)		
		X	Y	Z
1	C	-0.89713	-1.68179	-0.2267
2	C	0.062375	-2.50004	0.603321
3	C	0.549828	-1.84115	1.911077
4	C	0.873863	-0.34127	1.829321
5	C	1.741698	-0.0434	0.609702
6	N	1.496002	1.088124	-0.12857
7	C	0.465546	2.080582	0.21215
8	C	-0.84811	1.632856	-0.43634
9	O	-1.64771	1.052877	0.467636
10	C	-2.69355	0.147111	0.045142
11	C	-2.20854	-1.25558	0.419117
12	C	-0.67722	-1.36496	-1.5047
13	H	-1.36485	-0.79143	-2.11446
14	Cl	0.742378	-1.81607	-2.41296
15	C	-3.96494	0.538214	0.77377
16	H	-2.82726	0.259622	-1.03398
17	H	-2.09483	-1.28463	1.510064
18	H	-2.99527	-1.97964	0.170618
19	H	-0.43103	-3.44655	0.863523
20	H	0.932916	-2.74787	-0.00553
21	H	-0.19755	-1.96171	2.704794
22	H	1.442933	-2.38502	2.236703
23	C	1.618762	0.117167	3.090378
24	H	-0.07337	0.191911	1.764719
25	O	2.659134	-0.80924	0.322575
26	C	2.201455	1.26016	-1.39652
27	H	0.330767	2.083433	1.294216
28	C	0.842906	3.48846	-0.23341
29	O	-1.0988	1.745694	-1.61416
30	H	1.039763	-0.13652	3.983342
31	H	2.590344	-0.38173	3.153935
32	H	1.78845	1.198967	3.090882
33	H	1.477314	1.422175	-2.20044
34	H	2.761047	0.349224	-1.5943
35	H	2.893182	2.106475	-1.35199
36	H	0.87386	3.569653	-1.32128
37	H	0.094155	4.192679	0.138016
38	H	1.815422	3.765403	0.180471
39	H	-4.25957	1.556239	0.509399
40	H	-3.81576	0.484528	1.856249
41	H	-4.77575	-0.14174	0.499189

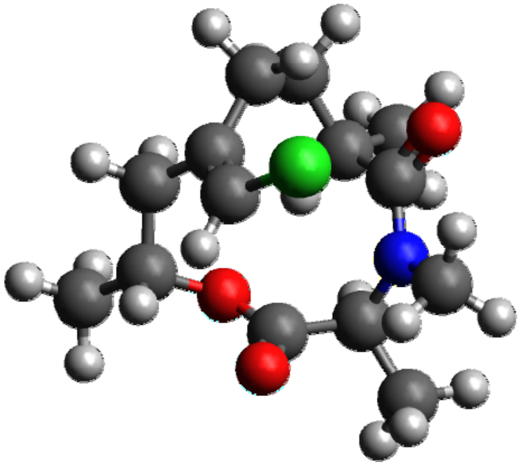

Table S56. Cartesian Coordinates For The Optimized Conformer 15 Of Compound 3*R*,6*R*,11*S*-**10t**

#	Atomic	Coordinates (Angstrom)		
		X	Y	Z
1	C	-2.01466	0.519482	-0.69478
2	C	-1.19097	-0.7283	-0.94484
3	C	-0.98017	-1.68561	0.256285
4	C	0.387871	-1.57422	0.954355
5	C	1.465465	-1.73532	-0.11652
6	N	2.359689	-0.72211	-0.34528
7	C	2.499539	0.458136	0.51748
8	C	1.552384	1.540281	-0.00333
9	O	0.419908	1.565815	0.704601
10	C	-0.63583	2.465455	0.293016
11	C	-1.39086	1.884694	-0.90785
12	C	-3.31589	0.47026	-0.40317
13	H	-3.94335	1.344978	-0.27052
14	Cl	-4.20942	-1.02148	-0.24552
15	C	-1.50044	2.669653	1.521072
16	H	-0.16844	3.405999	-0.0167
17	H	-2.16665	2.613544	-1.16736
18	H	-0.70329	1.834378	-1.75958
19	H	-1.69556	-1.2895	-1.74047
20	H	-0.22295	-0.42968	-1.3644
21	H	-1.75741	-1.52297	1.00968
22	H	-1.086	-2.71669	-0.09606
23	C	0.545439	-2.672	2.010398
24	H	0.436331	-0.60007	1.436797
25	O	1.47329	-2.75256	-0.8073
26	C	3.177344	-0.77634	-1.5553
27	H	2.190626	0.186247	1.526706
28	C	3.933479	0.972704	0.575994
29	O	1.780782	2.24641	-0.95994
30	H	-0.27014	-2.6147	2.737022
31	H	0.521103	-3.65602	1.534175
32	H	1.491831	-2.57448	2.551628
33	H	3.149008	0.195099	-2.05566
34	H	2.76124	-1.53847	-2.21057
35	H	4.215282	-1.0362	-1.32656
36	H	4.259044	1.369985	-0.38671
37	H	3.994063	1.778302	1.31201
38	H	4.60594	0.169216	0.885508
39	H	-0.91338	3.11059	2.329856
40	H	-1.91417	1.716569	1.862078
41	H	-2.32884	3.343158	1.284132

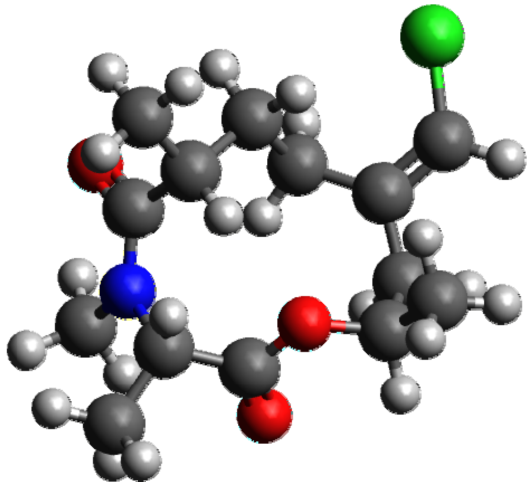

Supplement: Supplementary file 1 [file marinedrugs-17-00423-s001.pdf]
